# Digital health literacy in medical education: a scoping review of current challenges and development strategies

**DOI:** 10.1186/s12909-026-08903-7

**Published:** 2026-03-02

**Authors:** Guanli Xie, Jianglong Liao, Xiaoxia Tang, Yanfang Yang, Fu Han, Duo Liu, Deng Li, Yaju Jin, Tao Wang

**Affiliations:** 1 Yunnan University of Chinese Medicine, Kunming, Yunnan China; 2 Kunming Municipal Hospital of Traditional Chinese Medicine, Kunming, Yunnan China

**Keywords:** Digital health literacy, Medical education, Digital healthcare capability

## Abstract

**Objective:**

The purpose of this research is to answer the following questions: (1) Identify the key components of digital health literacy (DHL) in medical education. (2) Assess the current state and challenges of DHL in this field. (3) Explore ways to improve DHL among medical education participants.

**Methods:**

This research adheres to the Arksey and O’Malley framework and PRISMA-ScR guidelines of the EQUATOR network. A thorough search was conducted across Medline, Embase, CINAHL, Education Research Complete, Teacher Reference Center, and ERIC, covering studies from January 1, 2015, to June 12, 2025. Two authors independently screened and selected eligible studies based on the PCC (Population/Participants, Concepts, and Contexts) framework. A coding framework was developed, and the included literature was coded. Based on these codes, qualitative analysis was conducted to synthesize the findings according to the three key research questions. During the processes of literature retrieval, screening, and data extraction, all tasks were independently carried out by two authors. In cases where discrepancies emerged, the final decisions were made through discussions involving a third author.

**Results:**

A total of 123 articles were included in the study. The findings indicated that major challenges in digital literacy within medical education include the lack of standardized frameworks, varying literacy levels, core competency gaps, and a disconnect between attitudes and behaviors. Other issues are low motivation for practical use, uneven scenario coverage, and regional and specialty disparities. Barriers to improvement include resource limitations, environmental adaptation, equity issues, cognitive and acceptance challenges, curriculum problems, and inadequate teacher skills. While some intervention strategies show initial promise, their generalizability is limited due to mostly small-scale, short-term studies lacking comprehensive, long-term research. To address these limitations, we have proposed recommendations for enhancing digital literacy from multiple perspectives, grounded in a thorough review of the literature.

**Conclusion:**

This study reviewed a decade of literature on digital literacy in medical education, focusing on framework and definition, research status, challenges, and solutions. It provides guidance for future research and provides practical strategies for medical education institutions to enhance digital literacy training, optimize programs, and address developmental gaps, thereby supporting the digital transformation of medical education and the cultivation of interdisciplinary talent.

**Supplementary Information:**

The online version contains supplementary material available at 10.1186/s12909-026-08903-7.

## Introduction

In the context of global healthcare undergoing an unprecedented digital transformation, digital medical education faces new challenges. Digital technologies have deeply penetrated every link of medical services, ranging from electronic health records (EHR)-assisted diagnosis and remote medical treatment to artificial intelligence (AI) applications [1, 2]. The COVID-19 pandemic further accelerated this trend [3–7]. However, in sharp contrast to the rapid digitalization of clinical practice, the medical education system lags significantly in cultivating the digital health literacy (DHL) of medical students [8, 9].

DHL is defined as “an individual’s ability to search for, understand, evaluate health information from electronic resources and apply this knowledge to solve health problems” [10, 11]. In medical practice, this ability is manifested in multiple skills such as the effective use of EHR, the rational application of telemedicine tools, and the critical evaluation of online health information. The lack of sufficient DHL may prevent medical staff from making full use of digital tools to optimize the diagnosis and treatment process, and may even lead to improper clinical decisions due to misjudging online health information [12–14]. Moreover, within the contemporary landscape of medical digitalization, patients’ dependence on digital health tools, encompassing online consultations, report interpretation, and health management, necessitates that healthcare professionals possess the requisite digital literacy [15]. These professionals must not only demonstrate proficiency in utilizing digital tools but also be capable of instructing patients in their proper use and addressing inquiries related to digital health. Consequently, the imperative for “digital literacy as demanded by patients” serves as the primary impetus for prioritizing the development of digital literacy within medical education.

Medical education, as a crucial stage for cultivating future doctors, urgently needs to systematically integrate digital health content to bridge the “digital divide” [16–18]. The international consensus statement points out that the digital health capabilities framework should include four major areas, 19 core capabilities, and 178 learning outcomes [19]. However, medical curriculum reform worldwide faces many challenges, including limited curriculum space, insufficient teaching staff, and a lack of infrastructure [20]. Against this background, a comprehensive analysis of the current situation of cultivating DHL in medical education and the exploration of effective educational strategies are of great significance for promoting the digital transformation of medical care.

This study employed the method of literature review, systematically retrieved relevant literature, and screened the included literature according to the PCC principles, according to the scoping review methodology framework as proposed by Arksey and O’Malley [21, 22]. The included literature was then subjected to qualitative analysis to answer the following questions: (1) What constitutes the fundamental components of DHL within the context of medical education? (2) What is the present state of DHL in medical education, and what challenges are currently encountered? (3) What strategies or interventions can be implemented to improve the DHL of individuals engaged in medical education? Additionally, this paper integrated existing research, summarized effective educational strategies, and innovative practical methods. It also proposed future development paths and suggestions. The main purpose is to provide evidence-based references for medical educators and policy makers, thereby contributing to the development of this crucial field of medical education.

## Methods

This study employs the scoping review methodology framework as proposed by Arksey and O’Malley [21, 22] and reported according to RISMA-ScR guidelines of the EQUATOR network [23]. Following the inclusion of pertinent literature, the analysis was conducted using a “two-step coding-theme extraction” process.

### Data source

The process of screening literature was conducted in strict accordance with the methodological guidelines of the Preferred Reporting Items for Systematic reviews and Meta-Analyses (PRISMA). A comprehensive and systematic search was performed across multiple online databases, including Medline (via PubMed), Embase (via Embase.com), CINAHL (via EBSCOhost), Education Research Complete (via EBSCOhost), Teacher Reference Center (via EBSCOhost), and ERIC (via EBSCOhost) from Jan 1, 2015 to Jul 12, 2025.​ The search strategy of Medline (via PubMed) is reported in Table 1. The search strategies for other data are provided in Supplementary Material 1.

**Table 1:** Search strategy of medline via PubMed

NO	Search strategy
#1	“Health Literacy” [MeSH Terms]
#2	“Health Literacy” [Title/Abstract]
#3	“Digital Technology” [MeSH Terms]
#4	“Digital Technology”[Title/Abstract] OR “Digital Technologies”[Title/Abstract] OR “Technologies, Digital”[Title/Abstract] OR “Technology, Digital”[Title/Abstract] OR “Digital Electronics”[Title/Abstract] OR “Electronics, Digital”[Title/Abstract] OR “Information Technology”[MeSH Terms] OR “Information Technology”[Title/Abstract] OR “Digital Health Literacy”[Title/Abstract] OR “eHealth Literacy”[Title/Abstract] OR “Digital Health Competence”[Title/Abstract] OR “Health Information Technology Literacy”[Title/Abstract] OR “Digital Health Skills”[Title/Abstract] OR “Digital Health Literacy Skills”[Title/Abstract]
#5	#1 OR #2 OR #3 OR #4
#6	“education, medical” [MeSH Terms] OR “education, medical, undergraduate” [MeSH Terms] OR “education, medical, graduate” [MeSH Terms] OR “clinical clerkship” [MeSH Terms] OR “internship and residency” [MeSH Terms] OR “curriculum” [MeSH Terms] OR “competency - based education” [MeSH Terms] OR “problem - based learning” [MeSH Terms] OR “students, medical” [MeSH Terms] OR “faculty, medical” [MeSH Terms] OR “teaching” [MeSH Terms] OR “clinical competence” [MeSH Terms]
#7	#7 “education, medical” [Title/Abstract] OR “education, medical, undergraduate” [Title/Abstract] OR “education, medical, graduate” [Title/Abstract] OR “clinical clerkship” [Title/Abstract] OR “internship and residency” [Title/Abstract] OR “curriculum” [Title/Abstract] OR “competency - based education” [Title/Abstract] OR “problem - based learning” [Title/Abstract] OR “students, medical” [Title/Abstract] OR “faculty, medical” [Title/Abstract] OR “teaching” [Title/Abstract] OR “clinical competence”[Title/Abstract]
#8	#6 OR #7
#9	#5 AND #8
#10	#9 AND (English[Language]) AND (2005:2025[pdat])

### Literature screening

All the screened literature was managed using the EndNote software (EndNote X 7.4). Duplicate records were removed, followed by screening according to predefined inclusion and exclusion criteria. Specifically, two authors (XXT and YFY) independently screened titles and abstracts to exclude clearly irrelevant studies. Remaining studies underwent full-text reviews to confirm eligibility. Disagreements between the two authors were resolved through discussion with a third experienced author (TW).

### Inclusion criteria and exclusion criteria

The inclusion and exclusion criteria of the literature were carried out according to the PCC (Population/Participants, Concepts, and Contexts) framework according to the guideline [21, 22].

### Inclusion criteria

#### Population/participants

Research subjects included medical education stakeholders (implementers and learners), specifically undergraduate/postgraduate medical students, nursing/pharmacy students, clinical trainees (interns, residents, standardized training physicians), educators (medical school faculty, clinical preceptors, nursing training instructors), registered nurses, and other healthcare professionals.

#### Concepts

Literature focused on one of four themes: (1) connotation and core competencies of DHL; (2) current DHL levels among stakeholders (students, educators, healthcare professionals, and policymakers); (3) challenges in improving DHL; (4) strategies for enhancing medical DHL. Both theoretical and empirical studies were included.

#### Contexts

##### Scope

Medical education scenarios (classroom teaching, clinical practice, online education, etc.).

##### Discipline/field

Clinical medicine, preventive medicine, medical education studies, etc.

##### Time/method

To ensure the timeliness, most articles were published between Jan 1st 2015 and June 12th 2025.

### Study design

The included studies had clear research objectives/methods and underwent peer review. Eligible study types included cross-sectional, qualitative, descriptive, reviews, and case reports studies; letters to the editor and personal opinions were excluded. Only English-language articles published between 2015 and 2025 were included.

### Exclusion criteria

Studies were excluded if they: (1) Targeted non-medical education populations (general public, non-medical professionals, animals/cells) or healthcare providers’ clinical practices (e.g., electronic medical record usage) without explicit links to educational training/curriculum design; or focused on stakeholders not directly engaged in medical education (e.g., policymakers or technical developers) unless their roles were directly related to DHL training for learners/educators. ​(2) Lacked explicit reference to DHL as a defined competency (e.g., vague allusions to “digital health” without establishing links to core skills: information evaluation, digital tool operation, or data literacy) or was rooted in non-educational/non-digital frameworks (e.g., sociological analyses of health disparities without addressing DHL educational interventions). (3) Were conducted exclusively in non-educational settings (e.g., clinical/administrative/technical contexts such as 5G remote surgery, AI algorithms optimization) without exploring educational implications for medical learners; described traditional medical education methodologies (e.g., in-person lectures, non-digital ward rounds) or generic curriculum reforms without DHL integration; or were non-peer-reviewed materials (news articles, editorials, blog posts) or failed to report clear DHL-related educational outcomes (e.g., focusing solely on patient outcomes/technological functionality) due to inadequate methodological rigor.

### Data extraction

A standardized extraction form was crafted utilizing Microsoft Word, encompassing the following fields: (1) basic literature information (identifier, publication year, region, study design); (2) coding details (primary/secondary codes, subdimensions, mention frequency); (3) raw data (quantitative: percentages, sample sizes, effect sizes; qualitative: participant quotes, thematic statements); (4) evidence source (page number, table/figure number, section title). Two authors (FH and DL) extracted data from included studies, with verification by a third author (YJJ).

### Data coding process

A two-level coding framework was established based on the research aims and a comprehensive review of existing literature (Table 2). Expert review (5 experts in digital health education and qualitative research, I-CVI = 0.9) confirmed its content validity. The final coding system includes four primary codes, eight secondary codes, and predefined subdimensions for each secondary code to guide consistent data extraction.

**Table 2:** The code system of the study

Primary Code	Secondary Code	Scope of Core Content
1Theoretical Research	1.1 Definition and core domain of Digital Health Literacy	Definition of core concepts/essential characteristics of digital literacy in medical education; medicalized interpretation of general digital literacy and its adaptation basis
	1.2 Framework System and Adaptive Adjustment of Digital Health Literacy	Construction/validation of digital literacy frameworks for medical education; adaptive transformation of general frameworks (e.g., DigComp, ISTE) to medical scenarios
2 Status Description	2.1 Digital Health Literacy Levels of Different Stakeholders	Objective evaluation of digital literacy levels among medical students, educators, and healthcare professionals
	2.2 Current Status of Application Scenarios	Objective description of digital literacy application in medical teaching, clinical practice, and research training
3 Challenges	3.1 External Challenges	External obstacles (inadequate policies, technical resource shortage, medical data security regulations)
	3.2 Internal Challenges	Internal barriers (insufficient educator literacy, curriculum integration difficulties, uneven student acceptance)
4 Solutions and Practices	4.1 Theoretical Recommendations	Unimplemented strategies (curriculum reform directions, educator training ideas)
	4.2 Practical Interventions	Implemented measures with effectiveness verification (curriculum design, training programs, digital tool application)

Two researchers with ≥ 3 years of experience in digital literacy research performed coding after 3 days of training. Training covered in-depth analysis of the secondary coding framework, clarification of dimension definitions, coding logic for representative literature, and pre-coding exercises (10 articles, with discrepancy refinement via feedback). Post-training, inter-coder consistency was good (kappa = 0.90). Discrepancies were resolved through discussion with a third researcher to reach a consensus.

All 123 included studies were systematically coded via full-text review, with relevant content mapped to the predefined framework. Coding guidelines were as follows: (1) For studies referencing the same secondary code across multiple subdimensions, each unique mention per subdimension was counted and extracted separately; (2) Detailed notes were recorded for ambiguous content (e.g., unclear region, overlapping subdimensions) to facilitate subsequent verification; (3) All extracted data were cross-referenced with original literature to ensure accuracy.

After coding all included studies, descriptive statistical analysis of coding results was performed first, quantifying total coded documents and mention frequencies for all coding tiers (primary, secondary, subdimensions). Research topics were then synthesized based on coding patterns and mention frequencies.

## Results

**Fig. 1:**
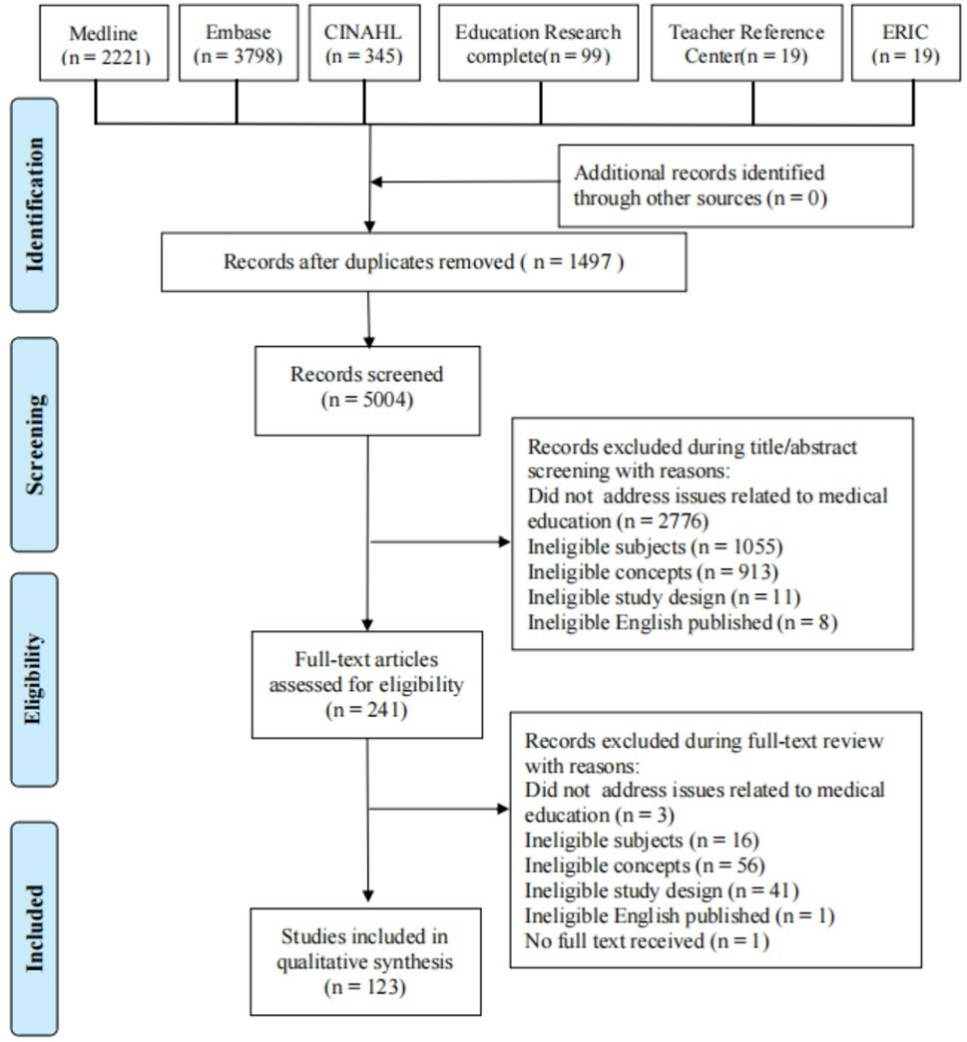
Flow diagram of the study selection process

**Fig. 2:**
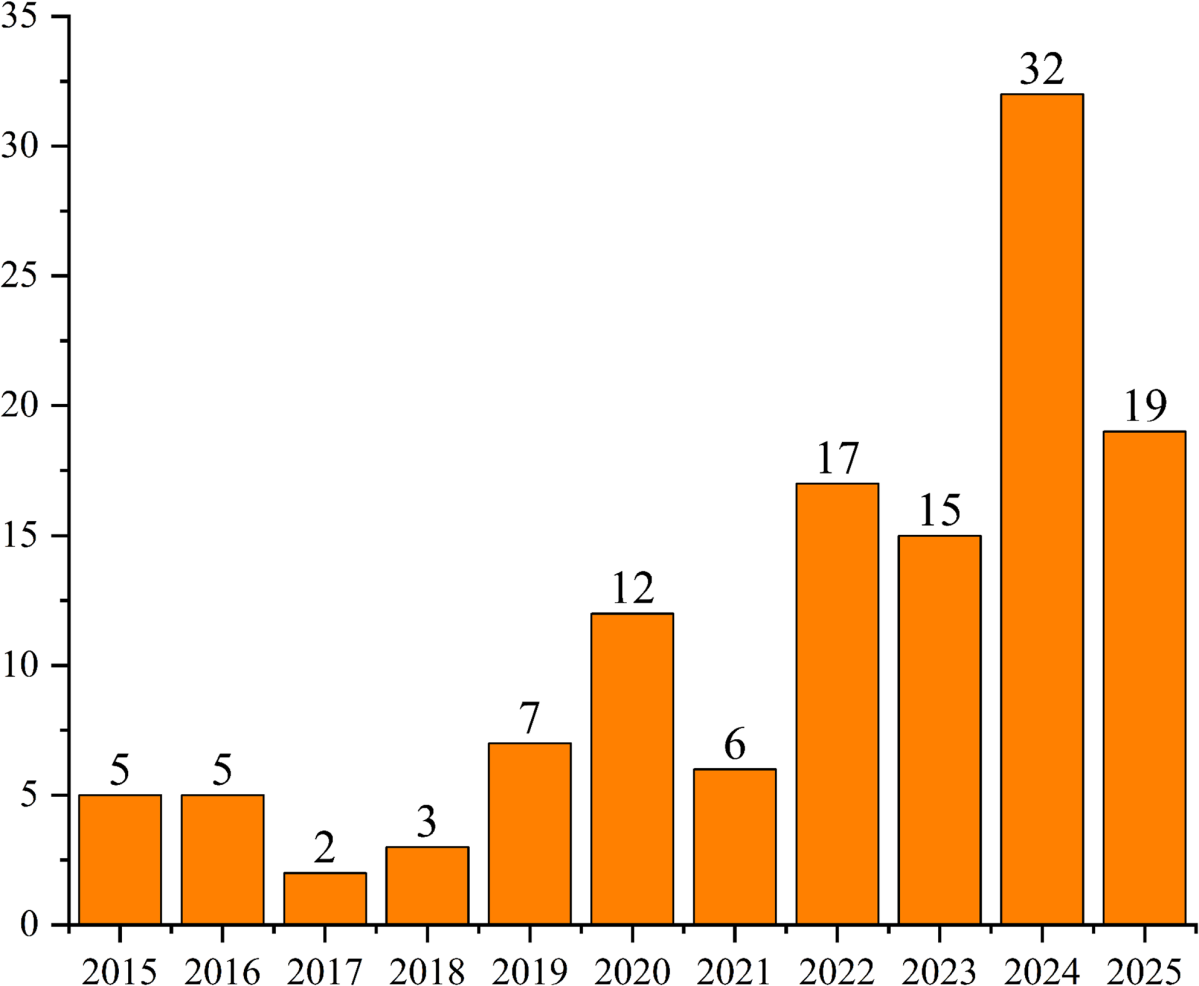
Distribution of included studies by year of publication

A total of 5004 documents were retrieved, and 123 of them were included after screening (Fig. 1). Annual publication volume increased steadily from 2015 (Fig. 2). According to the Center for Evidence-Based Medicine, included study types were cross-sectional (*n* = 96), review (*n* = 17), experimental (*n* = 6), and other (*n* = 4). Leading countries by publication volume (top 8) were the United States (*n* = 11), the United Kingdom (*n* = 11), Australia (*n* = 10), Iran (*n* = 9), Germany (*n* = 9), China (*n* = 9), South Africa (*n* = 6), and Singapore (*n* = 5). Study subjects mainly included medical undergraduates, postgraduates, surgeons, internal medicine doctors, nurses, pharmacy students, healthcare workers, medical teachers, and medical instructors. Basic characteristics and the included study list are provided in Supplementary Material 2; per-study coding information is available in Supplementary Material 3.

**Fig. 3:**
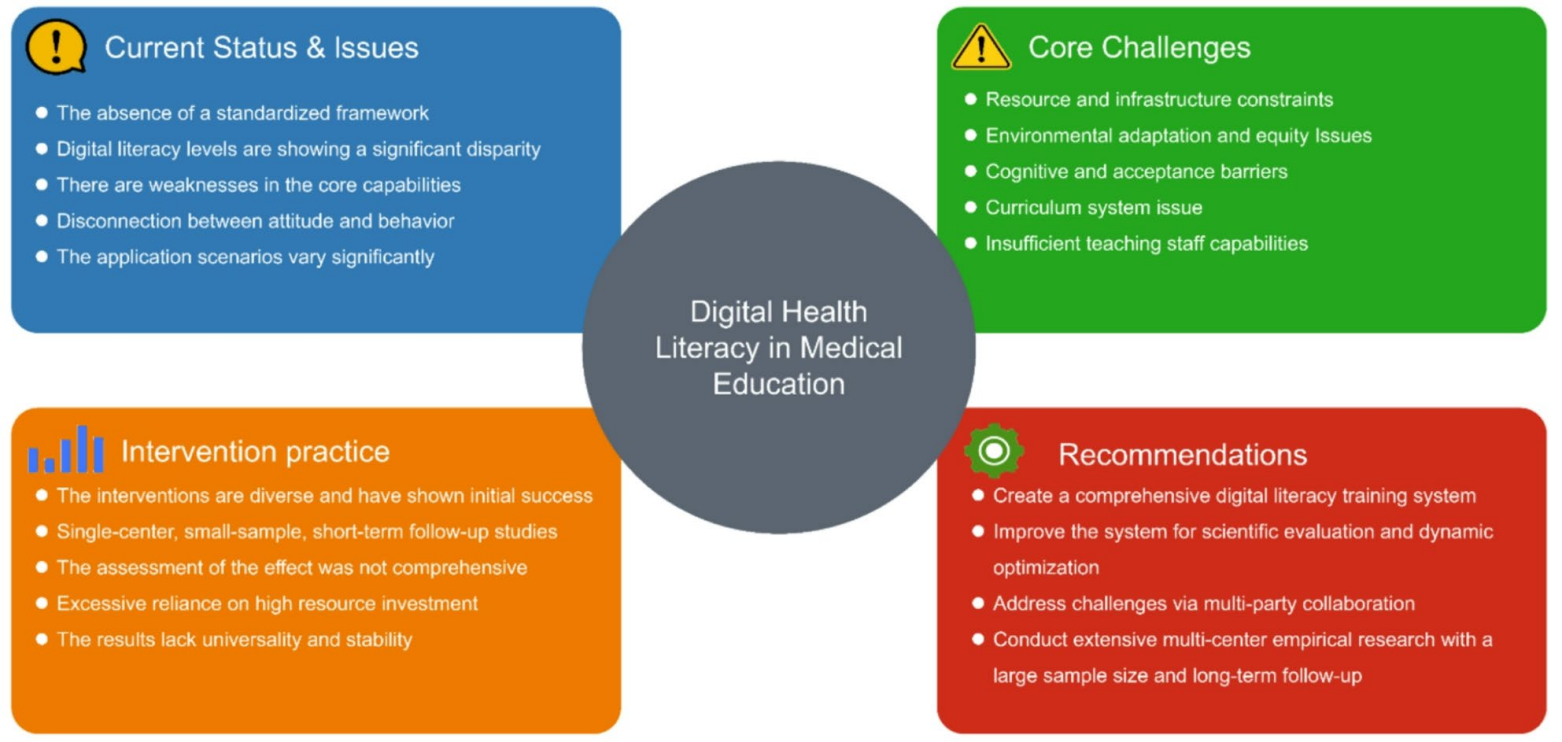
The core finding of this study

Coding summaries and mention frequencies are presented in Table 3; detailed secondary code frequencies are shown in Supplementary Material 3. Coding results and the mentioned data were analyzed to summarize the study’s four themes; core findings are illustrated in Fig. 3.

**Table 3:** The summary of the coding and the frequency of mention

Primary Code	Secondary Code	The number of records	The frequency of mention
1Theoretical Research	1.1 Definition and core domain of Digital Health Literacy	12	48
	1.2 Framework System and Adaptive Adjustment of Digital Health Literacy	12	62
2 Status Description	2.1 Digital Health Literacy Levels of Different Stakeholders	58	243
	2.2 Current Status of Application Scenarios	22	92
3 Challenges	3.1 External Challenges	7	31
	3.2 Internal Challenges	31	140
4 Solutions and Practices	4.1 Theoretical Recommendations	64	277
	4.2 Practical Interventions	19	90

## The definition and framework system of digital literacy

### The core dimension of digital health capabilities

Twelve of 123 studies focused on digital literacy core dimensions (48 mentions) [1, 24–34]. Literature analysis revealed the core categories of digital literacy are multi-dimensional, cross-scenario, and practical characteristics, encompassing five interrelated core modules frequently cited in the literature.

#### Basic digital technology and tool application

The basic digital technology and tool application is the core foundation of digital literacy. All 12 articles(100% coverage) [1, 24–34] directly or indirectly referenced its sub-dimensions, which included three directions associated with this concept: basic IT operations (such as computer literacy and the use of digital devices), professional digital tool applications (such as electronic medical records (EMR), clinical information systems, and educational digital tools), and digital technology comprehension (including cognition of IT systems and infrastructure, as well as mastery of digital technology principles). Notably, studies identify this as the primary sub-dimension, confirming that “technology implementation” is a prerequisite for digital literacy [28, 32, 33].

#### Health information and data management

Health information and data management is the core module of digital literacy in health. Eleven articles (91.7% coverage) addressed this domain [1, 24–28, 30–34], which covers the entire health data lifecycle: acquisition (e.g., health information navigation and online medical information retrieval), processing (e.g., data recording, classification, and integration), management (e.g., electronic health record (HER) management, data storage, and retrieval), and evaluation (e.g., assessment of data relevance, reliability assessment, and adaptability to digital technologies). Studies emphasize precise health information processing, underscoring the “domain specificity” of digital literacy [1, 25, 30].

#### Ethics, law, and security regulation

Ethics, law, and security regulation constitute the fundamental components of risk management within the realm of digital literacy. Ten articles (83.3%) explicitly address these sub-dimensions [1, 24, 26–30, 32, 33], focusing on data privacy protection (including patient information confidentiality, data security measures), legal and regulatory compliance (such as adherence to medical data-related regulations, and ethical guidelines), and information governance (including data usage rights, and compliance with operational norms). Emerging topics (e.g., AI application ethics, algorithmic bias mitigation) were also explored in some studies. Studies identify this as a crucial sub-dimension, emphasizing the health sector’s need for effective risk control [27, 30, 32].

#### Collaboration and communication

Collaboration and communication extend digital literacy to social interactions. Nine studies (75%) [1, 26–28, 30–34]., addressed this dimension, which includes cross-professional digital collaboration (e.g., collaboration among medical staff, and educators), digital communication (e.g., virtual consultations, online information transmission, and synchronous/asynchronous digital communication), and digital service advocacy (e.g., promoting the adoption of digital technology within teams and facilitating cross-institutional information sharing). Notably, studies underscored digital literacy’s “interactive nature”, emphasizing it as a collective rather than an isolated individual skill [1, 30, 32].

#### High-level capability (leadership, innovation, and future adaptation)

High-level capability is an advanced of digital literacy addressed in seven studies (58.3%) [1, 26, 27, 30, 31, 33, 34], and reflecting digital literacy’s developmental nature. Core themes include digital leadership and management, such as digital health project management, and team digital capability building, the application of innovative technologies, including the use of novel digital teaching tools, and the practice of emerging technologies like AI, and future-oriented adaptation, which involves keeping pace with technological iterations, developing framework alignment capabilities, and engaging in long-term digital strategic planning. Notably, researches underscore the essential requirements of high-level digital literacy for “leading change” and “adapting to the future” [26, 33, 34].

### Research status of the DHL framework system and adaptive adjustment

Twelve included studies addressed the DHL framework system and adaptive adjustment. Based on our study’s framework and 30 others reviewed by Nazeha [32], most frameworks targeted nurses in high-income countries. Literature analysis identified three core characteristics of this dimension: diversified framework construction, scenario-specific adaptation, and practical adjustment mechanisms. It encompasses four core modules: framework definition/composition, evaluation system, practical adaptation, and optimization adjustment. All frameworks demonstrated clear specificity in applicable populations and scenarios. A detailed list of framework-specific applicable scenarios is provided in Table 4.

**Table 4:** Summary of target audiences and practical scenarios for digital health literacy frameworks mentioned in studies included

Study ID	Region	Target Population	Applicable Scenarios
Bleijenbergh 2023 [24]	Belgium, The Netherlands, and UK	Learners related to digital health literacy (no specific occupation specified, including diverse groups)	General digital health application scenarios (covering skill practice, competency assessment, and group difference analysis, etc.)
Car 2025 [25]	Multiple countries and regions	Educators (teacher group)	Teaching scenarios (utilization of electronic teaching aids, assessment of teachers’ informatics competency, and improvement of teaching-related digital literacy)
Davies 2022 [26]	UK	Professionals requiring Motivational Interviewing (MI) training (e.g., healthcare providers, educators)	Training and intervention scenarios (MI training design, training effect evaluation, and training promotion and implementation)
Hübner 2016 [27]	USA	Nursing and Inter-Professional Informatics	Scenarios integrating teaching and digital health (AI-related teaching applications, and utilization of digital health tools in teaching)
Jidkov 2019 [28]	UK	School teachers and students	School education scenarios (implementation of Digital Didactic Tools (DDT) courses, cultivation of teachers’ and students’ digital skills, and allocation of campus digital resources)
Jimenez 2020 [29]	Singapore	Populations in need of online medical information (e.g., general public, patients, healthcare-related learners)	Medical information acquisition and application scenarios (retrieval of online medical information, information evaluation, and practical application of medical information)
Lawrence 2024 [1]	USA	Healthcare practitioners and relevant institutional personnel	Healthcare service scenarios (promotion of health equity, learning of Digital Health/Health Informatics (DH/HI) courses, and alignment with the 2030 health vision)
Lee 2024 [30]	Korea	Digital health-related practitioners (including professionals with diverse educational backgrounds and occupations)	General digital health practice scenarios (data processing, privacy protection, and multi-dimensional digital skill application)
Lokmic-Tomkins 2024 [31]	Australia	Interprofessional digital health participants (e.g., healthcare providers, educators, and management practitioners)	Interprofessional collaboration scenarios (joint cultivation of digital health competencies, multi-stakeholder collaborative implementation, and integration of teaching and practice)
Nazeha 2020 [32]	Singapore, Netherlands, and UK	Reviewed 30 frameworks. Eight were for healthcare professional, and 14 were for nursing.	Clinical practice scenarios (clinical simulation training, integration of drug information, collaborative medical learning, and self-directed learning improvement)
Saaiq 2024 [33]	Pakistan	Educators (teacher group, including those from diverse institutions and teaching units)	Digital teaching scenarios (cultivation of digital teaching competencies, integration of subject teaching with digital technology, and assessment of teaching competencies)
Scott 2023 [34]	Australia	Digital health practitioners, policymakers, and relevant researchers	Digital health system construction scenarios (definition of competency frameworks, sorting out core concepts, and optimization of system gaps)

#### Core components of the framework system: definition, dimensions and structure

Eight studies (66.7%) explicitly addressed DHL framework core components [1, 26, 27, 30–34], following a logical sequence of “concept definition - dimension division - structural design”. For instance, Scott 2023 directly defined “digital health capabilities”, noting the framework’s goal of covering requirements from basic to advanced skills [34]. In contrast, Davies 2022 and Lee 2024 indirectly clarified the framework via dimension decomposition, highlighting its “domain adaptability” [26, 30]. The framework dimensions featured a “general + specialized” duality. General dimensions (technical application, ethical compliance, and information management) align with 1.1 coding core categories. Specialized dimensions are tailored to specific contexts, as exemplified by Saaiq with the “digital teaching capability dimension” [33], and Lawrence 2024 with the “DDoH oriented fairness dimension” [30]. Most frameworks use a hierarchical structure [34], with other adopt a “modular structure” [32].

#### Evaluation system: tools, indicators, and difference analysis

Eleven articles (91.7%) deal with relevant content on DHL assessment [1, 24–33], forming a complete framework of “assessment tools-core indicators-difference analysis.” The assessment tools are categorized into three types: specialized measurement scales [26], ability classification standards [33], and practical operation assessments [30]. Tool design was tailored to research contexts and target populations. Core assessment indicators focused on four areas: basic characteristics (demographics, educational background, occupational category), ability level (total score, sub-dimension score, skill proficiency), behavioral attitude (such as information acquisition behavior, application confidence, training participation), and adaptability (including course coverage gap, and resource allocation level). Additionally, group differences (demographics, institution type, teaching unit capability), and dimension correlations sub-dimensions and overall ability). This provides data support for framework adaptation and refinement [25, 29].

#### Practice adaptation: scenarios, resources, and implementation strategies

Ten articles (83.3%) focused on the practical implementation and adaptation of the framework [1, 25–33]. The central theme revolves around “Scenario Adaptation-Resource Adaptation-Implementation Adaptation”. For scenario adaptation, frameworks were closely aligned with clinical practice [32], teaching implementation [33], and school education [28]. Notably, a generalized adaptation description is absent. Resource adaptation focused on hardware infrastructure [31], course resources [1], and tool resources [25]. The key focus was aligning resource supply with framework requirements. Implementation strategies included curriculum integration [31], teaching method optimization [31], and training intervention design [26], ensuring framework feasibility.

#### Adaptive adjustment: gap identification and optimization direction

Nine articles (75%) address the adaptive adjustment of the framework [1, 26, 27, 29–33], forming a “gap identification-optimization suggestions” cycle. Key identified gaps included: content gaps (e.g., insufficient course coverage, inadequate inclusion of emerging technologies), resource gaps (e.g., insufficient allocation of DDT resource, incomplete infrastructure), group adaptation gaps (e.g., insufficient consideration of specific groups’ capabilities), and conceptual gaps [34]. Optimization adjustments focused four aspects: (1) framework content (incorporating emerging technologies, refining ethical compliance requirements); (2) implementation strategies (enhancing MI training, optimizing course structure); (3) resource supply (improving resource allocation, perfecting infrastructure); (4) evaluation tools iteration (refining indicators for difference assessment, optimizing the accuracy of measurement tools).

## The current state of digital literacy research

### DHL levels of different stakeholders and influence factors

Fifty-eight records focused on DHL levels of different stakeholders, with a sample that encompasses three primary stakeholder groups: students, educators, and healthcare practitioners. The student group included nursing students (11 articles [31, 35–44]), medical students (19 articles [30, 45–62]), and dental/pharmacy/health science students (8 articles [63–70]), covering undergraduate to graduate levels. The educator group (nursing instructors, medical university faculty, and health science educators) was represented in 10 articles [56, 71–79], with teaching experience of 1–30 years. The healthcare practitioner group (doctors, nurses, public health professionals, and medical interns, mostly hospital-based) was documented in 9 articles [80–84]. The remaining studies included mixed stakeholder groups. The research covered 27 countries/regions, with balanced representation of developed and middle/low-income nations.

Studies suggested that healthcare professionals generally hold positive attitudes towards digital transformation services (AI, telemedicine, and other digital technologies) [35, 50, 62, 80, 85]. Most students and professionals were willing to integrate digital health knowledge into curricula and desired training in medical care and digital technologies [40, 44, 46, 55, 61, 69, 79, 80]. In terms of DHL levels, participants’ digital literacy, nursing informatics competency, and e-health literacy were mostly intermediate [41, 49, 56, 63, 74, 76, 80, 86], with some groups displaying lower proficiency [40, 55, 72]. Participants were proficient in basic tasks (e.g., internet access, device usage, information retrieval [36, 38, 39, 44, 48, 49, 57, 66, 70, 71, 77, 86], but struggle with information evaluation, data reliability assessment, information management, and comprehensive digital technology knowledge [36, 38–40, 44, 49, 55, 66, 67, 70, 83, 86]. DHL influencing factors varied across multiple dimensions, including gender, educational background, occupation, and prior experience. For instance, men generally outperform women in certain digital competencies and hold more positive attitudes towards AI [48, 50, 60, 62, 68], though these gender gaps narrowed with higher educational attainment [47]. Students and professionals with higher education and academic achievement tend to have superior digital skills [68, 71, 73, 75, 85, 87]. Additional factors included age [56, 62, 74, 75, 77, 79, 88], work experience [73, 75, 77, 87], formal training [52, 56, 85], access to necessary equipment [52], and computer skills [36, 52, 57, 75, 86]. Most respondents lacked systematic training or had limited training in digital health, information communication technology, and AI [39, 45, 56, 63, 64, 66, 80, 89]. This training gap reduced confidence in digital technology use among some groups [49, 61, 66, 80], impeding the full exploitation of digital technologies in healthcare and education sectors.

### Current status of application scenarios

#### The current status of implementing core application scenarios in the digitalization of medical education

A comprehensive “course teaching, clinical practice, and ability development” has been established for medical education, with each scenario featuring extensive application and differentiated promotion. In course teaching, online and blended learning were the predominant models [90–92]. Most medical professionals recognized their value; learning management systems and virtual lectures were widely adopted in universities (e.g., Switzerland, the United Kingdom), with integration accelerated by the COVID-19 [91, 93, 94]. Digital technology is deeply integrated into professional education, with augmented reality/virtual reality enhancing anatomy instruction in surgery, digital dental technology (DDT) supporting dental case management and training, and academic electronic health record systems improving nursing documentation skills [94–96]. Self-study tools (smartphones, tablets, mobile apps) are widely used, with YouTube, Facebook, PubMed, and Google as core academic information retrieval channels [51, 65, 69, 97–99].

In clinical practice and internship, digital systems support clinical decision-making, with EHR and clinical decision support tools widely used for medication management and treatment planning. Most physicians integrate personal experience with digital tools in patient care [99–102]. Simulation training and telemedicine are increasingly promoted: clinical simulation training improves interdisciplinary communication and patient safety identification abilities, and respondents were willing to integrate telemedicine into future clinical practice [90, 93, 100]. Digital technology enables interactive and collaborative practice. Within a structured educational framework, methods such as telemedicine, group discussions, and interprofessional interactions significantly improve students’ learning outcomes and self-confidence [91, 92, 100, 102, 103].

In capability assessment and development, an initial diversified assessment system has been established using methodologies such as patient care plan formulation, digital tools critical evaluation, and remote medical skill on-site assessment to comprehensively evaluate DHL and practical competencies [90, 104, 105]. The lifelong learning support mechanism is continuously refined. Mobile learning tools offer “real-time” learning assurances for practitioners but need adaptation to meet competency demands at different professional stages [93, 98].

#### Regional and professional differences in application scenarios

In terms of regional differences, developed nations (e.g., Switzerland, the United Kingdom) have advanced systems and established standardized frameworks like PROFILES [106], though institutional varies. In contrast, developing countries (e.g., China) have accelerated transformation through policy-driven IT-medical education integration and established national remote education platforms [64, 107]. However, rural regions still face equipment and network accessibility challenges [91, 98]. Professional disparities also exist: practical disciplines (surgery, dentistry, and nursing) emphasize the implementation of clinical techniques (virtual anatomy, digital medical records, and AI simulators) [94, 95, 101, 107]. In contrast, pharmacy prioritizes health informatics and cross-professional digital collaboration [90].

## Challenges

### External challenges

A total of 6 studies coded as 3.1(External Challenges). The findings of the studies indicate that external challenges predominantly manifest in several key areas. First, there are significant constraints related to fundamental resources and infrastructure, encompassing both hardware and network capabilities. In rural and remote regions, issues such as limited internet access and unstable network connections are prevalent. Additionally, certain mobile websites and applications exhibit inadequate compatibility, thereby hindering the effective utilization of digital tools [65, 98]. At the level of human resources, a notable shortage of information technology (IT) professionals and the overall deficit in healthcare personnel adversely impact the implementation of digital technologies [56, 108]. Second, deficiencies exist concerning institutional and policy support. Certain nations lack a cohesive framework for digital skills training, as evidenced by reports highlighting issues such as inconsistent policy strategies and suboptimal certification systems [109]. In some countries and regions, government support is limited, and regulatory measures are excessively stringent [108]. Moreover, challenges related to environmental adaptation and equity persist. For example, practitioners operating in rural and remote locations encounter significant technical difficulties. The deployment of digital technologies is obstructed by inadequate coordination, and there is an absence of mechanisms for cross-regional and cross-institutional integration [98, 108]. Furthermore, limited digital literacy contributes to a disparity in usage among various groups, and pronounced health inequalities in certain regions adversely impact the inclusivity of digital technologies [56, 108]. Finally, cognitive factors, acceptance, and their related limitations present significant challenges in the realm of digital literacy. For example, there is a notable lack of acceptance of digital health technologies among both patients and healthcare providers, compounded by negative biases concerning professional ethics in the context of social media usage [98, 108]. Finally, digital technology training often remains incomplete and insufficient, particularly in areas such as cybersecurity and data protection. This is evidenced by a report from Nguyen [56], which indicates that 42.6% of participants had not received training on electronic health applications, and there was a marked deficiency in emphasis on network security and data protection [108].

### Internal challenges

A total of 32 records contained the code 3.2 (Internal Challenges), and it was mentioned 138 times. Literature findings reveal that the internal challenges of digital literacy currently mainly manifest in four core dimensions, as elaborated below.

Firstly, the curriculum system lacks integration and standardized benchmarks. On a global scale, there is a discernible delay in the incorporation of courses related to digital content. A majority of educational institutions fail to include essential subjects such as digital health, AI, and IT security within their formal curricula. Only a limited number of institutions provide specialized courses, and the extent and comprehensiveness of course coverage exhibit significant variability [39, 95, 106, 110, 111]. Additional curricular flaws include insufficient flexibility in course design, outdated teaching methodologies, and a clear disconnection from clinical practice [111–115]. For instance, Alhur noted that the majority of medical schools in Saudi Arabia have not systematically integrated digital health courses, with only three institutions offering specialized courses in medical informatics [110]. Gillissen et al. further reported that approximately 38% of medical students in Germany are unable to answer questions related to digitalization and AI due to insufficient coverage of these topics in their coursework [50]. This curricular deficiency directly leads to students having significant deficiencies in core skills such as information evaluation and data management. Tanaka reported that 69.4% of undergraduate nursing students in Japan are unable to find high-quality health resources, and 80.2% lack the skills for resource assessment [43].

Secondly, teaching staff exhibit insufficient professional competence and poor adaptability to digital teaching, which has become a key bottleneck in educational digital transformation. The teaching staff is a key bottleneck in the digital transformation process, and research results show that the teaching staff generally have limited knowledge and skills in digital technologies, lack formal professional training, and hold skeptical attitudes towards digital education [116, 117]. Some teachers are unwilling or unable to master the emerging digital technologies due to factors such as age, heavy workloads, and insufficient time, and lack the methodological support for digital teaching, resulting in the ineffective integration of digital tools into the teaching process [104, 116]. Lilly et al. identified “lack of qualified teachers” and “teachers’ limited knowledge of IT” as the main obstacles to integrating IT into the curriculum [116]. Alowais et al. analyzed data from 14 pharmacy colleges in the UK, and found that 13 institutions mentioned “lack of digital professional knowledge”, and 10 mentioned “time constraints” [104]. Zainal also found that the absence of accessible, qualified mentorship is a key factor affecting the improvement of digital literacy [113]. Beyond general digital skills, educators also show inadequate understanding and low confidence in applying AI: Moreover, the research also shows that some teachers have insufficient understanding and confidence in using new technologies such as AI, and 60.9% of assistant physician teachers believe that their understanding of AI technology is at a medium to good level, but their confidence in using it is low [80].

Furthermore, resource guarantees are inadequate, characterized by deficiencies in hardware, software, and supporting systems, with resource constraints permeating the entire digital education process. Resource constraints run through the entire process of digital education. In terms of hardware, there are problems such as insufficient computer equipment, unstable network connections, and limited access to EHR [91, 113]. A study on the challenges faced by Indian medical professionals in online teaching and their acceptance levels indicates that 73% of the respondents reported encountering technical problems, and 56% of the respondents had issues with unstable network connections [91]. In terms of software and technical support, there are issues such as the lack of dedicated digital teaching resources, insufficient technical support, limited data resources, and inconsistent standards [112, 118]. The study explored the challenges perceived by teachers in southern Iranian medical colleges regarding the development and institutionalization of e-learning content, and the results showed that participants believed that “lack of standard equipment and physical space” and “insufficient technical support” were the main obstacles to the development of e-learning content [112]. In terms of funding, some institutions have difficulty introducing digital technologies due to cost issues. For example, the results of a survey of 27 dental colleges that implemented digital dental technology showed that 33% of the respondents mentioned cost limitations as an obstacle to implementing digital technology education [95].

Finally, cognitive and cultural lag exists, with a disconnection between digital awareness and practical behavior. Studies have shown that some educators and learners have biases in their understanding of digital education, and educators have doubts about the practicality of digital education and are worried about its impact on traditional teaching models. A study from Norway indicated that physical therapy teachers are skeptical of digital education, mainly viewing it as a threat to existing teaching practices [117]. A retrospective analysis of survey data on IT usage and knowledge from first-year students at an American dental school in 2009, 2012, and 2017 shows that although students frequently use IT, their level of IT security knowledge is low, and the data from the three-year investigation indicates a downward trend in this knowledge level [70]. At the same time, there are cultural factors such as bureaucratic inertia and resistance to change in the academic environment, and a lack of an ecosystem that promotes digital innovation. For example, a study conducted in Singapore from the perspective of physicians holding leadership positions in medical institutions identified and analyzed the obstacles to implementing digital health capabilities into the undergraduate curriculum of the Singapore Medical School. The results showed that “bureaucratic inertia” and “lack of an innovation promotion ecosystem” were the main obstacles to the implementation of digital health capabilities [119]. Some medical professionals’ excessive reliance on non-evidence-based sources, such as personal experience, hinders the implementation of digital literacy education. For example, a study from Iran indicates that 81.3% of doctors, 67.1% of resident physicians, and students stated that they use their own experience for medical practices, and only 26.5% of doctors and 16.9% of resident physicians and students frequently use databases such as PubMed and MEDLINE for patient care [99].

## Solutions and practices

### Theoretical recommendations

Sixty-four studies contained the code 4.1(Theoretical Recommendations), with a total of 277 mentions. These documents primarily offer constructive recommendations for digital literacy cultivation across four key areas, as follows.

#### Clear goals, core competencies, curriculum refinement, and standardization

Enhancing digital literacy requires clear goals, comprehensive core competency coverage, curriculum optimization, and standardized benchmarks, achievable through three key strategies. Several studies have highlighted that the absence of a well-defined competency framework results in unclear training objectives [38, 43, 110]. Therefore, we must establish a comprehensive three-dimensional competency framework for digital literacy and to develop standardized training objectives. This framework should encompass basic digital skills (e.g., electronic health record management, data retrieval), advanced application capabilities (e.g., utilization of AI tools for diagnostic support, remote medical practices), and ethical security literacy (e.g., protection of patient data privacy, assessment of information quality), refined with reference to existing standards [1, 32, 33, 38, 83, 115, 116, 118–120]. Secondly, curriculum optimization must move away from a single elective course model and adopt an integrated course model. This approach involves embedding digital literacy content across various courses, including foundational medicine, clinical medicine, and professional skills. For instance, the integration of anatomy with 3D digital models [94], the incorporation of AI-assisted diagnostic case analysis in diagnosticology [121, 122], and the embedding of electronic prescription system operations in nursing courses [38], ensure the tight integration of theoretical knowledge and practical application. Research indicated that integrated teaching methodologies yield significantly better outcomes compared to traditional, independent teaching approaches [116]. Additionally, interdisciplinary/cross-professional collaboration and the integration of cutting-edge technologies (e.g., cross-professional courses [32, 90, 92, 122, 123], AI medical application modules [68, 105, 121, 122, 124, 125]), further strengthen digital literacy.

#### Innovative teaching methods, emphasis on practical application, and promotion of active learning are critical for enhancing digital literacy

First, immersive and simulation-based pedagogies should be implemented, including remote medical communication simulations [46, 66, 77, 88, 94, 100, 105], digital practical assessments [83], 3D anatomical models, and VR clinical scenarios [117]. In nursing and medical education, electronic medical record simulation systems enable safe, repeated practice of operational procedures to improve skill proficiency [126]. Second, blended and personalized learning models, combining offline instruction, online resources (e.g., digital teaching platforms, professional applications [1, 90, 92, 105, 123]), and offline practical operations, support student self-directed learning [94, 107].

#### Strengthening resource and support systems is essential to overcoming digital literacy challenges and solidifying its implementation

First, hardware and technical support should be enhanced by ensuring stable campus networks, improving digital device accessibility, supporting remote-area students with equipment, and developing shared digital teaching platforms [30, 33, 34, 40, 41, 43, 51, 57, 59, 67, 69, 70, 85, 87, 118, 123, 124, 127], measures that effectively reduce existing barriers. Second, specialized educator training (e.g., AI tools, digital teaching methodologies [1, 30, 38, 51, 116, 124], addresses core challenges such as “lack of qualified teachers” and “insufficient technical capabilities”. Third, integrating clinical and industry resources, via medical institution partnerships to develop internship platforms (providing access to authentic EHR and telemedicine systems) and involving industry experts in curriculum development [30, 86, 87, 90, 126], ensures educational content aligns with real-world practice.

#### Enhancement of the evaluation mechanism

Adopting diverse evaluation indicators and methodologies for digital literacy enhances the effectiveness of training programs. Empirical evidence shows that varied assessment techniques significantly improve the feedback precision on digital literacy training quality [42, 61, 92]. Second, developing appropriate assessment tools enables comprehensive digital literacy evaluation [39, 68]. Additionally, the assessment focus should be tailored to the diverse group’s needs. Finally, a feedback and iteration mechanism, regularly collecting input from students, educators, and clinical institutions [42, 43, 61, 85, 92, 119], to dynamically optimize course content and teaching methods, establishes an “evaluation-feedback-improvement” closed loop, supporting continuous training enhancement.

### Practical interventions

A total of 19 documents contained the code 4.2(Practical Interventions), with 90 total mentions. Current research on medical education practical interventions exhibits two key characteristics, detailed below.

#### Diversification of intervention forms and carriers

##### Course-based interventions are the primary approach for enhancing digital literacy

These interventions primarily encompass graded elective courses, integrated curriculum systems, and massive open online courses (MOOCs). Graded elective courses address varying proficiency levels (foundational, advanced, and cutting-edge) to meet diverse learning needs [123, 127–130]. Some institutions have developed vertically integrated curriculum incorporate practice-oriented courses, electives, and domain-specific courses, to fully integrate digital literacy with professional education [128]. Additionally, digital competence MOOCs, freely accessible to a broad audience, have attracted over 4,000 registrations and provide general digital literacy training [26].

##### Specialized training programs and workshops enhance digital literacy

Blended training is developed for professionals(e.g., physicians and head nurses) to improve digital competencies [131, 132], while short-term context-specific interventions [37, 133, 134], such as intensive care unit nursing informatics training [133], and digital hand hygiene self-training modules [134], rapidly enhance essential practical skills.

##### Digital tools augment existing intervention strategies to improve digital literacy

Digital tools complement intervention strategies: academic EHR systems [96], evidence-based practice digital tools(e.g., S4BE [135]), and immersive platforms(e.g., Jupyter Notebooks [26]), strengthen learning-application integration, incorporating assessments(electronic health literacy (eHL), health evidence-based medicine (FEBM-IH)) into curricula enables “assessment-driven learning” [136], further improving digital literacy.

#### Current interventions focus on the “Skills-Thinking-Ethics” as three core objectives in digital literacy training

Basic digital skills, EHR operation, digital tool use (e.g., wearable device data analysis [137]), and health information retrieval/evaluation [120] are the intervention’s foundation. Advanced application thinking involves critical digital health reasoning [120], innovative application skills [127], and translating digital research into clinical practice [123]. Ethical and security literacy development focuses on [130]and patient privacy protection [138].

## Discussion

The research findings reveal a lack of standardized frameworks for digital literacy within medical education. Existing frameworks typically encompass multiple dimensions, including “basic technology, information management, ethical security, collaborative communication, and higher-order abilities.” However, significant discrepancies exist among different studies regarding the categorization, nomenclature, and hierarchical relationships of these dimensions. Furthermore, there is no consensus on the essence of these frameworks. General digital literacy frameworks often overlook medical-specific requirements, such as health information management and the application of clinical digital tools. In contrast, specialized frameworks tend to overemphasize technical operations, inadequately addressing dimensions such as ethical security, collaborative communication, and higher-order innovation, or failing to incorporate emerging technologies, such as AI. Secondly, existing frameworks exhibit limited adaptability and fail to adequately address the diverse needs of various stakeholders, such as students, educators, and medical practitioners, as well as different professions, including nursing, surgery, and pharmacy, and distinct regions, such as developing and developed countries. Furthermore, targeted adjustment mechanisms are absent. Several factors may contribute to this issue. Firstly, digital literacy is an interdisciplinary domain that encompasses medicine, IT, education, and ethics. The varying disciplinary perspectives of researchers lead to divergent emphases in framework development [32]. Researchers with a medical background tend to prioritize clinical application compatibility, those with an IT background focus on technical operational aspects, and those with an educational background emphasize teaching implementation and skill development. Consequently, there is a lack of cross-disciplinary consensus. Secondly, the rapid evolution of digital health technologies outpaces the updates to existing frameworks, thereby hindering the timely integration of relevant dimensions of these emerging technologies. Current research predominantly consists of regional and small-scale studies, which lack the scope of global and multi-center collaboration, making it challenging to establish a unified framework standard [24, 32]. Furthermore, some studies excessively depend on literature reviews and expert consultations without sufficient empirical data verification and optimization, resulting in a disconnect between theoretical frameworks and practical application [24]. Additionally, most countries have not yet developed a unified DHL framework guideline, which leads to a lack of policy guidance and industry standards [32]. Consequently, research institutions often operate independently, resulting in fragmented frameworks. As recommended in the literature, coded as 4.1, there is a clear call for the establishment of a comprehensive digital literacy framework that integrates “basic digital skills, advanced application capabilities, and ethical and security literacy.” This suggestion precisely addresses the core issues of the current framework. Its rationality lies in the fact that a unified framework can solve the problem of fragmented research and provide a unified benchmark for teaching, assessment, and practice.

The findings of Topic 2 reveal three fundamental issues concerning digital literacy. Firstly, there is a “dual polarization” phenomenon in the digital literacy levels among various stakeholders, characterized by a “weakness in core skills.” Specifically, while the overall digital literacy levels across different stakeholder groups are moderate, there exist substantial disparities between these groups. Concurrently, all groups exhibit common deficiencies in advanced skill areas, particularly in core competencies such as information evaluation (e.g., discerning between high-quality and low-quality health resources), data management (e.g., integration and analysis of EHR), and ethical security (e.g., data privacy protection and cybersecurity knowledge) [40, 43, 66]. These deficiencies represent significant barriers to the enhancement of digital literacy. Secondly, a notable disjunction exists between the attitudes and behaviors of stakeholders and the inadequate motivation for practical application. Specifically, while the majority of students and medical practitioners demonstrate a high level of acceptance towards digital technology and express willingness to engage in related training, there remains a substantial gap between knowledge and action in practical application [80, 83]. On one hand, the absence of systematic training and practical opportunities results in certain groups lacking the confidence necessary to effectively apply digital tools, thereby hindering the integration of digital skills into routine teaching and clinical practices. On the other hand, some practitioners exhibit an over-reliance on non-evidence-based sources, such as personal experience, which leads to a diminished trust in digital tools. Consequently, the application scenarios for digital technology are constrained, preventing its full potential from being realized in terms of aiding decision-making and enhancing efficiency. Thirdly, there exists an “uneven coverage” of application scenarios and “significant differences” across regions and specialties. From a regional perspective, the implementation of digital technologies, including blended learning and simulation training, is more prevalent and advanced in developed countries, where standardized frameworks have been established [55, 104]. Conversely, in developing nations, particularly in rural areas, limitations in infrastructure and resource availability hinder the accessibility and depth of digital technology applications [91, 139]. From a disciplinary perspective, practical fields such as surgery, nursing, and dentistry exhibit a more extensive and common integration of digital technologies, including AR/VR anatomy and digital medical records. In contrast, disciplines that are predominantly theoretical demonstrate a lower degree of digital literacy integration, leading to an increasingly pronounced disparity in digital literacy levels across different specialties.

Drawing upon the findings from Topic 3, the prevailing challenges in advancing digital literacy encompass constraints related to resources and infrastructure, difficulties associated with environmental adaptation and equity, cognitive and acceptance limitations, curricular system inadequacies, and insufficient teacher competencies. The fundamental causes of these challenges may be attributed to several factors. Primarily, economic development disparities serve as the principal cause of resource constraints. In particular, developing nations and rural regions experience limited financial investment, which hampers the development of digital infrastructure and the updating of equipment, thereby creating disparities in the accessibility of digital technologies [91]. Secondly, policy-making in the digital health sector has not kept pace with technological advancements. The majority of countries have yet to establish a comprehensive policy framework encompassing “training - certification - application,” resulting in a lack of systematic planning for the development of digital literacy [91]. As a nascent field, digital literacy has not been fully integrated into educational curricula. The pace of curriculum design and updates to teaching methodologies lags behind the rapid evolution of digital technology. Furthermore, the current teaching workforce predominantly comes from traditional medical education backgrounds and lacks formal training in digital technology. Some senior educators exhibit resistance to digital education due to entrenched professional habits and the perceived costs of learning new technologies [99]. Although younger educators may possess certain digital skills, they often lack the methodological support necessary for effective digital teaching, which impedes the enhancement of digital literacy.

In addressing the aforementioned challenges, the literature pertaining to Topic 4 offers pertinent theoretical recommendations. Specifically, to counter the absence of a unified framework, it advocates for the development of a comprehensive digital literacy framework encompassing “basic digital skills, advanced application abilities, and ethical and security literacy” to establish standardized training objectives [32, 92, 99, 115, 118]. Concerning the issue of “insufficient integration of the curriculum system,” the literature recommends the incorporation of digital literacy content into the professional curriculum, the innovation of teaching methodologies, an emphasis on practical application, and the stimulation of learner initiative [45–47, 122, 124]. To tackle the problem of inadequate resource support, it suggests enhancing resource and support mechanisms to overcome the bottlenecks in digital literacy development and to establish a robust foundation for implementation [40, 51, 59, 62, 83, 86, 92]. Furthermore, to address the challenges of “weak teacher capabilities”, it proposes the enhancement of digital capability training for educators [33, 78, 116, 117]. In response to the challenge of “insufficient institutional and policy support”, it proposes “formulating standardized training goals and assessment benchmarks” [62, 86, 124, 139].

Recent studies have undertaken practical investigations into methods for enhancing the digital literacy of medical professionals. The findings indicate that current intervention strategies for improving digital literacy are varied and have achieved preliminary success. Empirical research has shown that interventions such as course-based approaches (including graded elective courses, integrated courses, and MOOCs), specialized training (such as blended training and short-term skills workshops), and digital tool empowerment (including electronic health record simulation systems and S4BE evidence-based tools) can effectively enhance digital literacy across different groups [26, 127, 129, 131, 132, 134]. Furthermore, empirical studies generally identify fundamental digital skills, advanced application thinking, and ethical security literacy as core training objectives. Nonetheless, current empirical research still faces several challenges. Firstly, the empirical studies included in this analysis are predominantly single-center, small-sample, exploratory investigations with short-term follow-up. They lack multi-center, large-sample, long-term tracking research, which results in limited generalizability and stability of the findings. The sample populations are primarily composed of undergraduate students and professionals from urban medical institutions, with insufficient representation of the elderly, rural residents, and niche specialties such as public health and rehabilitation. This limitation hinders the ability to accurately reflect the diverse needs of different demographic groups. Secondly, the evaluation of intervention effects is not comprehensive. Existing studies predominantly focus on knowledge acquisition and skill enhancement, while insufficient attention is given to changes in attitudes, behavioral modifications, and the long-term practical application of interventions. Furthermore, the assessment tools employed lack standardization, with many studies utilizing self-developed scales or a single assessment method, thereby complicating the ability to conduct cross-study comparisons of intervention outcomes. Simultaneously, there is an absence of comprehensive cost-benefit analyses regarding the intervention, which hinders the ability to provide a reference framework for promoting such interventions in regions with limited resources. Additionally, the majority of empirical studies are characterized by their short-term nature, lacking mechanisms for long-term operation and iteration, thereby rendering the sustainability of intervention effects challenging. Furthermore, certain intervention measures are excessively dependent on substantial resource investment, such as high-end virtual reality equipment and professional simulation systems, which exhibit limited adaptability to developing countries and resource-constrained institutions, posing challenges for scalability.

## Suggestion

In light of the prevailing challenges, we propose the following recommendations.

1. To develop a comprehensive digital literacy training system that enhances digital skills

By integrating the four-dimensional framework of “knowledge, skills, attitude, and collaboration” from Topic 1 with the “weak foundation and insufficient high-level skills” challenge identified in Topic 2, we propose a three-dimensional integrated system, “level-scenario-ability,” for digital literacy enhancement.

Level dimension: Covering the full educational continuum (primary-medical-continuing education), and a three-tiered training mechanism (foundation - improvement - refinement) is established. At the primary education level, we introduce “medical digital literacy enlightenment courses” (e.g., health data awareness, introduction to digital ethics). During the medical education phase, the focus shifts to enhancing “professionally-adapted digital skills” (e.g., clinical digital tools, scientific research data analysis). In the continuing education stage, emphasis is placed on “frontier technology application” (e.g., AI-assisted diagnosis, digital health management), facilitating a progressive enhancement of competencies.

Scenario dimension: The application scenarios are divided into four main areas: teaching, clinical, research, and public health. Each area has specific training content tailored to its needs. Teaching focuses on innovative digital teaching tools, clinical on optimizing diagnosis and treatment digitally, research on digital methods like big data and AI, and public health on health data monitoring and early warning.

Ability Dimension: The ability dimension highlights core skills such as using digital tools, evaluating health information, protecting data privacy, and cross-domain collaboration. Differentiated skills are tailored to specific professions and roles, like surgery, nursing, and public health, or positions such as student, teacher, and clinical physician.

2. Enhance the system for scientific assessment and dynamic optimization to improve digital literacy training quality

To tackle the issue of inconsistent assessment systems and the recommendation for standardized assessment, it’s crucial to enhance the scientific assessment and dynamic optimization system and improve digital literacy training quality. Develop a comprehensive multi-dimensional assessment framework covering knowledge, skills, attitude, practical application, and ethical compliance. Knowledge should be assessed through standardized tests and AI evaluations. Skills should be evaluated via scenario-based operations and digital tools, such as electronic medical record efficiency and AI diagnosis accuracy. Attitude should be measured through surveys and observations. Practical application should be assessed using clinical case analysis and job performance correlation. Finally, Ethical compliance should be evaluated through “scenario simulations and ethical defenses.” Additionally, a “digital assessment platform” is essential for automating assessments and visualizing results. This approach ensures a standardized system, improving digital literacy training quality.

Following the guidelines in Topic 4, a “Medical Digital Literacy Graded Certification” system is proposed, consisting of three levels: primary (basic skills), intermediate (professional application), and advanced (leadership and ethics). These certifications should be tied to students’ graduation, educators’ professional growth, and physicians’ career development. To ensure relevance, standards and assessments must be updated continuously to align with digital health advancements like AI and blockchain. Establish a strong feedback-optimization system and a Quality Monitoring Database for Training to track digital literacy and practical skills of students, educators, and medical professionals. Conduct regular surveys to gather stakeholder feedback on training content adaptability and teaching tool practicality. Form an Expert Consultation Committee to use monitoring data and feedback to dynamically adjust training programs, curriculum, and resources.

3. Differentiated response strategies for various stakeholders

The preceding discussion has outlined fundamental challenges and research directions in digital literacy for medical education. Given that students, educators, practitioners, administrators, and policymakers have distinct needs, targeted strategies are essential for comprehensive improvement.

## Recommendations for enhancing digital literacy among students

Students require “skill adaptation to professional development and flexible learning.” To address “adequate basic skills but deficiencies in advanced skills” (Topic 2), we propose:

### Undergraduate

Integrate “basic digital skills with ethical literacy” into core curricula (e.g., 3D anatomy models, pharmacology database retrieval, and digital privacy case discussions in medical ethics). Provide “fragmented learning resource packages” (mobile micro-lessons, tool guides) for self-directed study.

### Postgraduate/residents 

Emphasize “advanced application thinking and professional scenario adaptation.” Strengthen clinical digital tools (EHR optimization, AI-assisted diagnostics) and cross-professional collaboration (virtual consultations, multidisciplinary data sharing). Customize by specialty: surgery (AR/VR simulation), nursing (electronic documentation, remote care), public health (digital epidemiology, health data monitoring).

### Disadvantaged students

Offer “one-on-one digital mentorship,” portable training equipment, and offline resources to improve accessibility.

## Recommendations for enhancing digital literacy among medical educators

Educators face “insufficient digital skills, outdated methods, and low reform motivation” (Topic 3). Drawing on “teacher training with resource support” (Topic 4):

### Stratified training

Educators with limited digital skills should attend workshops on basic digital teaching tools, like online platforms and virtual courseware design, to improve their digital literacy. Those with basic digital knowledge should participate in innovative courses on blended teaching and simulation case development. Experts with advanced skills should engage in frontier technology training on digital health, focusing on AI teaching tools and metaverse scenario design, to become leaders in digital teaching.

### Parallel mechanisms for enhancing motivation and alleviating burdens

Integrate digital teaching into evaluations; establish a “Digital Teaching Innovation Award”. Allocate dedicated funds and paid training time. Create a “Digital Teaching Resource Sharing Platform” to reduce redundancy. Provide instructional designers and technical support.

### Cross-institutional and interdisciplinary collaboration

Form a “Digital Literacy Teaching Collaboration Community” across institutions to share courses, cases, and assessment tools through regular exchanges, addressing “single-person operation” isolation.

## Recommendations for enhancing digital literacy among medical practitioners

Practitioners need “practice-aligned skills and fragmented learning formats”. To address “positive attitudes but insufficient application” (Topic 2):

### Clinical scenario-based training

Develop “clinical digital skills modules” covering efficient EHR use, remote consultation, health information evaluation, and data privacy. Use case-based methods integrating real scenarios (AI-assisted diagnosis, e-prescription error prevention).

### Provision of lifelong learning resources

Establish a mobile platform with short videos, tool manuals, and ethical cases. Regularly update content on emerging technologies (generative AI for documentation, blockchain security) and regulatory changes.

### Job adaptability certification

To address “inconsistent assessment” and support “standardized assessment and certification,” a digital literacy grading system for medical practitioners is proposed. This system would have three levels: basic (competence in everyday clinical digital tools), intermediate (proficiency in using digital tools for diagnostics and treatment), and expert (leadership in digital health innovation). Certification outcomes could be linked to annual evaluations, career advancement, and continuing education credits, encouraging continuous learning and development.

## Administrative and policy recommendations

Education departments and policymakers are crucial in enhancing digital literacy. They must tackle issues like system gaps and resource disparities and incorporate insights on framework building and policy support. Recommendations include:

### Standardized national framework

Create a unified national framework and competency standards for digital literacy in medical education, detailing training goals and core requirements for different stages and groups. Develop a guide for integrating digital literacy into medical education, covering curriculum integration, teacher training, resource allocation, and assessment. Establish a mechanism for regular evaluations and feedback to improve digital literacy education policies continuously.

### Equitable resource allocation

Increase investment in digital infrastructure in rural and central/western institutions. Establish a “5G + Remote Training Platform” to share quality resources. Utilize government service procurement and school-enterprise partnerships to offer affordable digital teaching tools and open-source resources to under-resourced institutions. Propose a “Special Fund for Digital Literacy Development” to support curriculum development, teacher training, and aid for disadvantaged groups.

### Integrated policy coordination

Strengthen interdepartmental collaboration in education, health, and science to create a cohesive system for policy-making, resource allocation, implementation, and feedback. Align digital literacy with healthcare digital transformation by incorporating it into medical institution rankings and clinical pathway evaluations, promoting synergy between educational goals and industry needs.

## Future research directions

Future research should focus on several key areas to complement the limitations of current studies. First and foremost, more studies are needed in middle- and low-income countries, rural areas, and niche medical fields. Specifically, comparative research should explore digital literacy disparities across different regions and professions, with particular attention to vulnerable or understudied groups like elderly medical practitioners and students with disabilities, which will provide empirical support for developing targeted intervention strategies. Second, large-scale, multi-center, long-term randomized controlled trials (RCTs) are essential to systematically evaluate the effectiveness and cost-efficiency of various intervention methods, such as integrated courses versus specialized training, and high-cost VR training versus low-cost MOOCs. Concurrently, a unified digital literacy assessment framework with standardized evaluation tools is crucial, as it will enable consistent comparison and synthesis of findings across different studies. Third, research should explore how new technologies (e.g., AI, the metaverse) transform the connotation and requirements of digital literacy in medical education, which necessitates the development of tailored training programs and assessment tools adapted to these technological advancements. Additionally, a collaborative training model that combines technology empowerment with ethical norms is vital to ensure balanced development. Furthermore, in-depth research on the ethical implications of new technologies, such as data privacy protection in AI-driven educational tools and ethical norms in doctor-patient interactions within the metaverse, is needed to provide clear ethical guidance for digital literacy cultivation. Finally, promoting interdisciplinary and cross-sector collaboration is essential for advancing digital literacy in global medical education. By fostering research partnerships among universities, healthcare institutions, technology companies, and policy-making bodies, stakeholders can jointly develop high-quality training tools and quickly translate research findings into practical applications. Moreover, international collaboration is also key to comparing digital literacy training models across different cultural and policy contexts, which will enable the extraction of universal insights and the creation of localized adaptation strategies suitable for diverse regions.

## Limitations

While this study systematically reviews the past decade’s research on digital literacy in medical education, highlighting current challenges and offering recommendations, it has notable limitations, the first of which is limited literature coverage. Specifically, there is a lack of representation from low- and middle-income countries and rural areas, which may skew the analysis of digital literacy needs in these regions. Furthermore, most research focuses on fields like dentistry, clinical medicine, and nursing, with few studies on specialties such as public health and rehabilitation medicine; this oversight limits a comprehensive understanding of digital literacy characteristics and requirements across all medical disciplines. Beyond literature coverage issues, the body of reviewed research mainly consists of descriptive studies and theoretical ideas, rather than robust empirical evidence such as RCTs and long-term follow-up studies. This gap directly limits the ability to understand the long-term effectiveness and applicability of digital literacy intervention strategies. Additionally, many existing studies suffer from small sample sizes and inconsistent assessment methods, which further undermines the reliability of their findings. Finally, current research disproportionately focuses on emerging technologies like AI, the metaverse, and blockchain, without deeply exploring the expansion of digital literacy connotations, innovations in training methodologies, or the ethical issues posed by these technologies. This shortfall hampers the study’s ability to address the evolving needs of rapidly advancing digital health technologies.

## Conclusion

This study systematically reviewed literature from the past decade, focusing on four main themes: framework and definition, research status, challenges, and solutions in digital literacy within medical education. It summarized the current development and core issues, offering targeted recommendations. The study identified key challenges like standardization, competency, application, and resources, providing guidance for future research and practical strategies for improving digital literacy training in medical education. This aids in the digital transformation of medical education and the development of interdisciplinary medical professionals. 

## Supplementary Information

Supplementary Material 1.

Supplementary Material 2.

Supplementary Material 3.

## Data Availability

The data that support the findings of this study are available from the corresponding author upon reasonable request.

## Abbreviations

EHR: Electronic health records
AI: Artificial intelligence
MI: Medical informatics
DHL: Digital health literacy
ICT: Information and communication technology
HIT: Health information technology
IMIA: International Medical Informatics Association
NI: Nursing informatics
IT: Information technology
