# Supplementary material for: Digital health literacy in medical education: a scoping review of current challenges and development strategies
Source: BMC Med Educ. 2026 Mar 2;26:567. doi: 10.1186/s12909-026-08903-7 (PMC13059213; doi:10.1186/s12909-026-08903-7)
Supplement: Supplementary file 1 — Supplementary Material 1. [file 12909_2026_8903_MOESM1_ESM.docx]

All the search strategies of the databases

Medine via PubMed

#1 “Health Literacy” [MeSH Terms]

#2 “Health Literacy” [Title/Abstract]

#3 “Digital Technology” [MeSH Terms]

#4 “Digital Technology”[Title/Abstract] OR “Digital Technologies”[Title/Abstract] OR “Technologies, Digital”[Title/Abstract] OR “Technology, Digital”[Title/Abstract] OR “Digital Electronics”[Title/Abstract] OR “Electronics, Digital”[Title/Abstract] OR “Information Technology”[MeSH Terms] OR “Information Technology”[Title/Abstract] OR “Digital Health Literacy”[Title/Abstract] OR “eHealth Literacy”[Title/Abstract] OR “Digital Health Competence”[Title/Abstract] OR “Health Information Technology Literacy”[Title/Abstract] OR “Digital Health Skills”[Title/Abstract] OR “Digital Health Literacy Skills”[Title/Abstract]

#5 #1 OR #2 OR #3 OR #4

#6 “education, medical” [MeSH Terms] OR “education, medical, undergraduate” [MeSH Terms] OR “education, medical, graduate” [MeSH Terms] OR “clinical clerkship” [MeSH Terms] OR “internship and residency” [MeSH Terms] OR “curriculum” [MeSH Terms] OR “competency - based education” [MeSH Terms] OR “problem - based learning” [MeSH Terms] OR “students, medical” [MeSH Terms] OR “faculty, medical” [MeSH Terms] OR “teaching” [MeSH Terms] OR “clinical competence” [MeSH Terms]

#7 “education, medical” [Title/Abstract] OR “education, medical, undergraduate” [Title/Abstract] OR “education, medical, graduate” [Title/Abstract] OR “clinical clerkship” [Title/Abstract] OR “internship and residency” [Title/Abstract] OR “curriculum” [Title/Abstract] OR “competency - based education” [Title/Abstract] OR “problem - based learning” [Title/Abstract] OR “students, medical” [Title/Abstract] OR “faculty, medical” [Title/Abstract] OR “teaching” [Title/Abstract] OR “clinical competence”[Title/Abstract]

#8 #6 OR #7

#9 #5 AND #8

#10 #9 AND (English[Language]) AND (2005:2025[pdat])

Embase

#1 'Health Literacy'/exp

#2 (‘Health Literacy’):ti:ab

#3 'Digital Technology'/exp

#4 ('Digital Technology' OR 'Digital Technologies' OR 'Technologies, Digital' OR 'Technology, Digital' OR 'Digital Electronics' OR 'Electronics, Digital' OR 'Information Technology' OR 'Information Technology' OR 'Digital Health Literacy' OR 'eHealth Literacy' OR 'Digital Health Competence' OR 'Health Information Technology Literacy' OR 'Digital Health Skills' OR 'Digital Health Literacy Skills'):ti:ab

#5 #1 OR #2 OR #3 OR #4

#6 'education, medical'/exp OR 'education, medical, undergraduate'/exp OR 'education, medical, graduate'/exp OR 'clinical clerkship'/exp OR 'internship and residency'/exp OR 'curriculum'/exp OR 'competency - based education'/exp OR 'problem - based learning'/exp OR 'students, medical'/exp OR 'faculty, medical'/exp OR 'teaching'/exp OR 'clinical competence'/exp

#7 ('education, medical' OR 'education, medical, undergraduate' OR 'education, medical, graduate' OR 'clinical clerkship' OR 'internship and residency' OR 'curriculum' OR 'competency - based education' OR 'problem - based learning' OR 'students, medical' OR 'faculty, medical' OR 'teaching' OR 'clinical competence'):ti:ab

#8 #6 OR #7

#9 #5 AND #8

#10 #5 AND #8 AND [2005-2025]/py AND [humans]/lim AND [english]/lim

EBSCOhost（CHNAL, APA, ERIC）

S1 MH“Health Literacy”

S2 TI(“Health Literacy”)

S3 MH“Digital Technology”

S4 TI(“Digital Technology” OR “Digital Technologies” OR “Technologies, Digital” OR “Technology, Digital” OR “Digital Electronics” OR “Electronics, Digital” OR “Information Technology”[MeSH Terms] OR “Information Technology” OR “Digital Health Literacy” OR “eHealth Literacy” OR “Digital Health Competence” OR “Health Information Technology Literacy” OR “Digital Health Skills” OR “Digital Health Literacy Skills”)

S5 S1 OR S2 OR S3 OR S4

S6 MH“education, medical” OR MH“education, medical, undergraduate” OR MH“education, medical, graduate” OR MH“clinical clerkship” OR MH“internship and residency” OR MH“curriculum” OR MH“competency - based education” OR MH“problem - based learning” OR MH“students, medical” OR MH“faculty, medical” OR MH“teaching” OR MH“clinical competence”

S7 TI(“education, medical” OR “education, medical, undergraduate” OR “education, medical, graduate” OR “clinical clerkship” OR “internship and residency” OR “curriculum” OR “competency - based education” OR “problem - based learning” OR “students, medical” OR “faculty, medical” OR “teaching” OR “clinical competence”)

S8 S6 OR S7

S9 S5 AND S8

S10 SU human

S11 S9 AND S9

S12 Publication Date: 20000101-20251231
