# Supplementary material for: Digital health literacy in medical education: a scoping review of current challenges and development strategies
Source: BMC Med Educ. 2026 Mar 2;26:567. doi: 10.1186/s12909-026-08903-7 (PMC13059213; doi:10.1186/s12909-026-08903-7)
Supplement: Supplementary file 2 — Supplementary Material 2. [file 12909_2026_8903_MOESM2_ESM.docx]

Table 1 Characteristics of included studies in this review

| ID | **Country or region** | **Study type** | **Participants** | **Objective** | **Main Results** | **Conclusion** |
| --- | --- | --- | --- | --- | --- | --- |
| Abou Hashish 2024 [1] | Saudi Arabia | Descriptive correlational design | Nursing students | To assess the perceived knowledge, attitudes, and skills of nursing students regarding digital transformation, as well as their DHL and attitudes toward AI. | Nursing students exhibited good knowledge of and positive attitudes toward digital transformation services. They possessed strong digital skills, and their DHL and positive attitude toward AI were commendable. Overall, the findings indicated significant positive correlations between knowledge of digital transformation services and all the digital variables measured. Senior students reported greater digital knowledge and a positive attitude toward AI. | The study recommends an innovative undergraduate curriculum that integrates opportunities for hands-on experience with digital healthcare technologies to enhance their digital literacy and skills. |
| Afra 2024[2] | Iran | Cross-section survey design | Nursing students | To assess the informatics competency of nursing faculty members and its application in educating nursing students at Abadan Nursing Faculty. | The mean score of faculty members’ nursing informatics competency at the Nursing Faculty was 110 out of a maximum of 150, placing it within the proficient range. The highest score for nursing informatics competency was related to the informatics literacy dimension (47.65), while the lowest was related to the information management skills dimension (19.54). Information management skills (r = 0.881, *P* < 0.001), computer literacy (r = 0.871, *P* < 0.001), and informatics literacy (r = 0.976, *P* < 0.001) were significantly correlated with nursing informatics competency. Faculty members reported the highest use of multimedia content development (mean score = 4.19) and the lowest use of robotic surgery (mean score = 1.71) | Faculty members’ informatics competency was reported to be at a proficient level. However, due to advancements in technology and transformations in nursing care, it seems necessary to improve nursing faculty members’ abilities in all aspects, particularly in the information management skills dimension. It is recommended to hold faculty development programs regarding the integration of technology into the curriculum to elevate the quality of education. |
| Ahmad 2025[3] | Saudi | A comparative study | Medical students | To investigate the impact of MI training on Saudi medical students’ knowledge and attitude towards using technology in future medical practice. | The mean scores of knowledge and attitude towards MI were significantly higher among students who had undertaken the MI course compared to those who had not (*P* < 0.001). The results highlighted the effectiveness of MI training in enhancing students’ understanding of various MI concepts and applications, including electronic medical records, patient confidentiality, clinical decision support systems, telemedicine, e-prescriptions, evidence-based practice, and legal/ethical considerations in clinical informatics. | This result of this study highlights the significance of MI course in shaping students’ perceptions and understanding of various aspects of healthcare, particularly in the context of EMRs, patient confidentiality, and the role of technology in healthcare. The findings provide valuable insights into the transformative impact of MI training on students’ knowledge and attitude towards utilizing informatics in their future medical careers. |
| Alexander 2025[4] | Ireland | Qualitative study | Physician assistant/associate (PA) | To assess assistant/associate (PA) faculty perspectives on the impact of artificial intelligence (AI) | While knowledge of AI technology was measured as average to good by 60.9% of faculty, confidence in using it was low. Group differences in self-reported knowledge of AI was only significant by participants’ primary role in in their institution(X^2^(10) = 53.6, *P* ≤ 0.001). Participation in formal or inform a continuing education programs focused on AI appears to increase confidence levels among participants, although only 35% of respondents indicated participating in these activities. Most respondents (64.9%) expressed a positive view of AI technology in PA education, but did express concerns, particularly on student's development of critical thinking skills and academic integrity. The paper concludes by recommending training and support for faculty to harness AI’s potential and enhance PA education, ensuring that AI complements rather than replaces the critical human elements in teaching and learning. | The paper concludes by recommending training and support for faculty to harness AI's potential and enhance PA education, ensuring that AI complements rather than replaces the critical human elements in teaching and learning. |
| Alfallaj 2022[5] | Saudi Arabia | Cross-sectional survey | Chairpersons of prosthetic sciences departments | To investigate the status of digital dental technology (DDT) adoption in Saudi Arabian undergraduate dental education. A secondary objective was to explore the impact of dental schools’ funding sources to incorporate digital technologies. | Of the 27 dental schools (18 public and 8 private), 26 responded to the questionnaire (response rate: 96.3%). The geographic distribution of the respondent schools was as follows:12 schools in the central region, 6 in the western region, and 8 in other regions. Seventeen schools secure and preserve patients’ records using electronic software, whereas nine schools use paper charts. Seventeen schools (64,4%) implemented DDT in their curricula. The schools that did not incorporate DDT into their undergraduate curricula were due to not being included in the curriculum (78%), lack of expertise (66%), untrained faculty and staff (44%), and cost (33%). | This national study showed that digital components still need to be integrated into Saudi Arabian dental schools’ curricula and patient care treatment. Additionally, there was no association between funding sources and the DDT implementation into the current curricula. Consequently, Saudi dental schools must emphasize the implementation and utilization of DDT to align with Saudi Vision 2030 for healthcare digitization and to graduate competent dentists in digital dental care. |
| Alghasani 2023[6] | Oman | Cross sectional survey | Medical sciences students | To record the perception and attitude of medical sciences students studying in Oman towards the use of medical and health information on the internet | The internet was used as source for medical literature and medical information along with other activities in 71.1%% of students and PubMed and Google were used as trusted search engines. The majority of students (96.9%) tend to check the accuracy and reliability of internet medical and health information through searching for particular information in two or more websites. 72.1% of the students believe that the internet offers great potential to meet academic needs and promote learning. The statistical analysis showed a significant statistical correlation (*P* = 0.0001) in certain aspects of the perception and attitude of the participating students towards the utilization of medical and health information on the internet, | Although majority of students were trained on utilizing medical and health information during their university education, it seems this exposure varies between the four academic majors. Educational institutions in Oman are encouraged to design their e-health literacy program based on the individual requirements of medical sciences curricula. |
| Alhur 2024[7] | Saudi Arabia | A document analysis methodology | Medical students | To analyze the extent to which digital health and health informatics are integrated into the study plans of medical colleges across Saudi Arabia, in alignment with the national goals outlined in Vision 2030. | The analysis revealed that only a few medical colleges in Saudi Arabia have integrated dedicated courses on digital health and health informatics into their curricula. For instance, Dar Al Uloom University, Princess Nourah bint Abdulrahman University, and University of Hail offer specific courses on medical informatics. However, the majority of the institutions either lack such courses or offer them in a limited capacity. The extent and depth of coverage of these courses vary widely among the institutions that offer them. | The study highlights the need for a more standardized and comprehensive approach to integrating digital health and health informatics into the medical curricula of Saudi Arabian universities. Recommendations include expanding course offerings, developing standardized curricula, investing in faculty development, and utilizing advanced technologies. Addressing these gaps will better prepare medical graduates for the modern, technology-driven healthcare environment and align with the national objectives of Vision 2030. |
| Alipour 2022[8] | Iran | A descriptive and analytical cross-sectional study | Healthcare workers of teaching | To measure and evaluate the level of DHL among healthcare workers in teaching hospitals. | Healthcare workers gained in protecting privacy (89.7%), operational skill (83.9%), navigation skill (81.7%), and information searching (80.6%) of the total score, and were evaluated as very desirable in terms of these skills. Besides, they obtained in adding content (78.1%), determining data relevance (68.2%) and evaluating data reliability (64.8%) of the total score, which was indicative of a desirable level. In addition, there was a significant relationship between the level of DHL of healthcare workers and their level of education and job category (*P* < 0.05). | The overall DHL level of healthcare workers was assessed as desirable; therefore, the studied healthcare workers had an appropriate ability to help patients with self-care. Nevertheless, proper policymaking and planning for educating healthcare workers on how to evaluate the quality of health information in digital media and improving their skills of identifying relevant information among the vast amount of available health information by choosing the right search strategy can help promote healthcare workers’ literacy to an optimal level. |
| Alowais 2024a[9] | United Kingdom and Saudi Arabia | A mixed methods study | Pharmacy students | To explore the current and planned inclusion of digital literacy education in the undergraduate curricula of UK pharmacy schools. | Data from 14 pharmacy schools’ curricula were included in the analysis, with 10 reporting digital literacy education. Key themes identified from the analysed documents included understanding of health informatics, applied informatics, information technology skills, and the emerging digital health technology. Nineteen respondents from 16 schools participated in the survey; digital literacy inclusion was reported by 18 participants. There was variable alignment of digital literacy competencies with the Health Education England framework. Digital literacy was mainly integrated into existing teaching sessions, predominantly through self-learning (n = 12). Electronic Health Records and remote counselling were the main focus areas within the curricula. Challenges in implementing digital literacy include a lack of expertise (n = 13), and time constraints (n = 10). | The trend towards embedding digital literacy in UK pharmacy curricula is clear, but disparities suggest the need for a more unified strategy. Recommendations include establishing a specific digital literacy framework aligned with professional needs, improving accessibility and transparency in curricula documents, and investing in faculty development. |
| Alowais 2024b[10] | United Kingdom and Saudi Arabia | Scoping review | Pharmacy students | To identify the approaches used to integrate digital literacy into undergraduate pharmacy programs across different countries, focusing on methods for education, training, and assessment. | Out of 624 articles, 57 were included in this review. Educational and training approaches for digital literacy in undergraduate pharmacy programs encompassed a theoretical understanding of health informatics, familiarization with diverse digital technologies, and applied informatics in 2 domains: patient-centric care through digital technologies, and the utilization of digital technologies in interprofessional collaboration. Blended pedagogical strategies were commonly employed. Assessment approaches included patient plan development requiring digital information retrieval, critical appraisal of digital tools, live evaluations of telehealth skills, and quizzes and exams on health informatics concepts. External engagement with system developers, suppliers, and other institutes supported successful digital literacy education. | This scoping review identifies various learning objectives, teaching, and assessment strategies to incorporate digital literacy in undergraduate pharmacy curricula. Recommendations include acknowledging the evolving digital health landscape, ensuring constructive alignment between learning objectives, teaching approach and assessments, co-development of digital literacy courses with stakeholders, and using standardized guidelines for reporting educational interventions. This study provides practical suggestions for enhancing digital literacy education in undergraduate pharmacy programs. |
| Amare 2024[11] | Ethiopia | Survey study | Health professional educators | To evaluate the digital competency of health professional educators in Ethiopian medical education. | This study involved 498 health professional educators from 16 institutions. Most were male and taught clinical science. Over half worked in research institutions and held academic positions as assistant professors or above. About 10% were beginners in digital skills, with over half at an explorer level. Only a small percentage were experts. The majority had low digital skill competency, with only 7.2% showing high competency. There was no significant association between digital skill competency and factors like the type of institutions, program, course type or academic rank | There is a notable lack of digital skill competency among faculty members, with the majority lacking expertise in this area. Educators require guidance, collaborative encouragement, and knowledge exchange to enhance their digital competencies and effectively harness technology in their teaching. |
| Arienti 2021[12] | Italy | Observational pretest and post-test study. | Physiotherapy students | To evaluate the effectiveness of an Evidence- based practice (EBP) laboratory, using Students 4 Best Evidence (S4BE) as an educational tool, to teach EBP competencies to undergraduate physiotherapy students. | The students showed a significant improvement in all domains (*P* < 0.001), except in the sympathy domain, where the percentage score decreased from 71% to 60%. The best improvements were reached in terminology (54% to 65%) and in practice (41% to 55%) domains. | This study proposed an effective educational protocol, based on a DPBL approach, using S4BE as a digital technology tool. Further research is needed to test the effectiveness of this educational protocol compared with traditional learning methods for physiotherapy students. |
| Arthanareeswaran 2023[13] | India | Cross-sectional study | Medical professionals | To evaluate the challenges and acceptance of E-teaching by medical professionals during the COVID-19 pandemic. | Most of the participants (88%) agreed that the technological skills of giving online courses increase the educational value and experience of teaching medical professionals. The highest barrier to E-teaching was unsteady internet connectivity (56%), inadequate internet data (48%), lack of computers/ laptops (16.5%), and technical problems (73%%). The rate of participant agreement on perceived usefulness, perceived ease of use, and acceptance of E-teaching was (83.1%,81.4%, and 88.6% respectively). | In our current study, most of the participants strongly agreed with the perceived usefulness, perceived ease of use, and acceptance of E-teaching. It is evident that online teaching will persist, and education will increasingly adopt a hybrid model. |
| Asal 2025[14] | Saudi Arabia and Egypt | Cross-sectional study | Nurse educators | To investigate the relationships between digital competence, AI readiness and pedagogical innovation among nurse educators, with a specific focus on the moderating role of AI readiness. | Significant positive correlations were found between pedagogical innovation, digital competence (r = 0.546, *P* < 0.01) and AI readiness (r = 0.530, *P* < 0.01). Digital competence (*B* = 0.558, *P* < 0.001) and AI readiness (*B* = 0.580, *P* < 0.001) significantly predicted pedagogical innovation. AI readiness moderated this relationship (*B* = 0.199, *P* < 0.001, ΔR² = 0.0057), amplifying the effect at higher levels of AI readiness (*B* = 0.66, *P* < 0.001). | Digital competence and AI readiness play critical roles in promoting pedagogical innovation. Strengthening AI readiness through targeted training can enhance digital tools adoption in nursing education. It is crucial to revise academic standards for curricula and nurse educators to include AI competence, ensuring effective integration of AI and digital tools in nursing education through targeted training and infrastructure improvements. |
| Aydınlar 2024[15] | Türkiye | A mixed-methods study | School of Medicine, Nutrition and Dietetics, Nursing, Physiotherapy and Rehabilitation, Psychology, Biomedical Engineering, Molecular Biology, and Genetics | To determine the digital literacy level and awareness of students receiving health-based education in university and to pave the way for supporting the current curriculum with courses on digital literacy when necessary. | The survey was completed by 476 students. Female students had less computer knowledge and previous coding education. Spearman correlation test showed that there were weak positive correlations between the years and the “software and multimedia,” “ethics,” “interest and knowledge” domains, and the average score. The students from Nursing scored lowest in the query after those from the Nutrition and Dietetics department. The highest scores were obtained by Biomedical Engineering students, followed by the School of Medicine. Participants scored the highest in “network” and “AI.” and lowest in “interest-knowledge” domains. | It is necessary to define the level of computer skills who start health-based education and shape the curriculum by determining which domains are weak. Creating an educational environment that fosters females’ digital knowledge is recommended. Elective courses across faculties may be offered to enable students to progress and discuss various digital literacy topics. The extent to which students benefit from the digital literacy-supported curriculum may be evaluated. Thus, health-based university students are encouraged to acquire the computer skills required by today’s clinical settings. |
| Bleijenbergh 2023[16] | Belgium, The Netherlands, and UK | A modified explorative e-Delphi study | Healthcare professionals | To establish items of the digital adaptability competency for healthcare professionals. | In round 1, eleven items were included to the final document. In round 2, ten items were included. In round 3, the panel unanimously agreed to add six items, one item was modified into two separate items. In total, 29 items were included in the final document. | The rather abstract concept of digital adaptability is now transformed into a more pragmatic concept of 29 items, reflecting the practical competencies of healthcare professionals necessary to be digital adaptable. |
| Bloomfield 2023[17] | Australia | A quantitative study employing a cross-sectional survey | Registered nurses’ | To explore the association between registered nurses’ attitudes to e-learning and self-regulated online learning skills on their attitudes towards the use of Communication Technology (ICT) in healthcare. | Participant’s levels of online self-regulated learning were positively correlated with attitudes to e-learning (r = 0.663, *P* < 0.001). Attitudes to e-learning (70.4, SD 11.5) were also positively predictive of ITASH (R^2^ = 0.306, *P* < 0.001), but online self-regulated learning was not contributory to the prediction of attitudes to ICT in healthcare. | It is recommended that educators involved in online learning focus on strategies aimed at promoting positive attitudes to e-learning and ICT prior to employing those aimed at developing online self-regulation skills. Further research exploring online learning and ICT needs in the workplace are required. |
| Bosch 2022[18] | Germany | Cross-sectional study | Physicians | To investigate if a structured blended-learning training for practicing physicians in Germany enhances both physicians’ knowledge about central aspects of the digital transformation (including awareness of personal possibilities to act) and their attitudes towards a more digitally empowered mindset. | Participants reported an increase in every knowledge domain, representing large effects (Hedges’g 1.06 to 2.82). Attitudes were partly shifted towards a more empowered mindset with decreased insecurity towards technological, legal, and ethical aspects of the digital transformation (Hedges’g −0.82 to −1.40). However, preparedness for the digital transformation remained low. | Generally, the hypotheses were confirmed. The presented on-the-job training had the desired effects on practicing physicians’ knowledge and attitudes. Nevertheless, additional empowerment and support are essential. |
| Brown Wilson 2020[19] | United Kingdom of Great Britain and Northern Ireland, and Australia | Scoping review of the literature | Health care students | To explore how undergraduate health care students use digital technology to deliver patient care during their clinical placements. | Seven studies involving medical or nursing/midwifery students were included in the review. Three studies evaluated the use of mobile learning devices in patient care with four studies evaluating the use of digital systems in practice. Due to the heterogeneity of studies, which used differing digital systems and instruments, the researchers decided the most suitable method of analysis was a narrative review. The results are explained using four key themes: student learning needs when using technology in practice; access to technology in placements; perceptions of using technology in placements; and impact of technology on patient care. | The use of digital systems in clinical settings creates challenges and benefits to student learning in delivering patient care. When students are prepared and facilitated to use digital systems, a sense of confidence and belonging to the team is fostered. Lack of availability and access to these systems, however, may impede students’ ability to be involved in all aspects of patient care. Limitations of the current review included the relatively low quality of the educational research being conducted in this field of research. Further quality research is needed to explore how students in the health care professions are supported in digital environments and how higher education institutions are adapting their curricula to meet the digital learning needs of health care students. |
| Buchgraber-Schnalzer 2025[20] | Austria | Mixed method study | Healthcare professionals | to share initial experiences in the didactic implementation of an online course in health professions education that is designed to strengthen the healthcare community in acquiring AI competences and enable ethical and legal discussion regarding AI. | The key factors for creating the online course were identified and recommendations and strategies to enhance AI literacy are given. | Online training courses offer the possibility to integrate AI literacy aspects into healthcare professionals’ education. Future work should focus on further evaluation. |
| Car 2025[21] | Multiple countries and regions | Qualitative study | Medical education experts | To develop an evidence-informed, consensus-guided, adaptable digital health competencies framework for the design and development of digital health curricula in medical institutions globally. | The DECODE framework uses 3 main terminologies: domain, competency, and learning outcome. Competencies were grouped into 4 domains: professionalism in digital health, patient and population digital health, health information systems, and health data science. Each competency is accompanied by a set of learning outcomes that are either mandatory or discretionary. The final framework comprises 4 domains, 19 competencies, and 33 mandatory and 145 discretionary learning outcomes, with descriptions for each domain and competency. Six highlighted areas of considerations for medical educators are the variations in nomenclature, the distinctiveness of digital health, the concept of digital health literacy, curriculum space and implementation, the inclusion of discretionary learning outcomes, and socioeconomic inequities in digital health education. | This evidence-informed and consensus-guided framework will play an important role in enabling medical institutions to better prepare future physicians for the ongoing digital transformation in health care. Medical schools are encouraged to adopt and adapt this framework to align with their needs, resources, and circumstances. |
| Chung 2017[22] | USA and South Korea | A quantitative research design with supportive qualitative research | Nursing students' and nursing faculty's | To identify the use of academic electronic health record systems in nurse education and to determine student and faculty perceptions of academic electronic health record systems in nurse education. | Quantitative and qualitative findings revealed that academic electronic health record systems regarding nursing documentation could help prepare students for the future of health information technology. | Meaningful adoption of academic electronic health record systems will help in building the undergraduate nursing students' competence in nursing documentation with electronic health record systems. |
| Curran 2019[23] | Canada | A mixed-methods case study | Health and human services professionals | To explore the adoption and use of mobile learning as a continuing professional development (CPD) activity | Respondents reported using a smartphone (53.8%), tablets (50.4%), YouTube (43.0%), and mobile apps (35.8%) for CPD. The highest-rated benefits of mobile learning included improved access to information (M = 3.51); potential for enhanced knowledge acquisition (M = 3.45); staying up to date (M = 3.44); and verifying information (M = 3.40). The greatest barriers included cost of some apps and resources (M = 3.07); websites/programs not functional on mobile devices (M = 2.84); workplace barriers preventing access to digital resources (M = 2.82); and social media use linked to negative perceptions of professionalism (M = 2.65). Interview respondents described the flexibility and convenience of mobile learning, the level of autonomy it offered, and the advantages of learning on their own time. Technical issues, particularly for rural and remote practitioners, and digital professionalism also emerged as potential barriers. | A systems model organizes the factors influencing the adoption and use of mobile devices and resources to support “just-in-time” learning. Addressing policies, practices, and regulations that enable or inhibit adoption of mobile learning for CPD may foster enhanced use to support better clinical decision-making, improved accuracy, and greater patient safety. |
| Davies 2022[24] | UK | Systematic literature review | Medical workers | To describe Manchester’s involvement in the development of digital competency frameworks and our digital transformation education programmes that we have created, including a Massive Online Open Course and a professional development course for England’s Topol Digital Fellows. | The University of Manchester has collaborated with Health Ed-cation England to develop competency frameworks to embed digital competencies to underpin the education of healthcare professionals. We have shown that digital education can be de-livered through a diverse range of educational interventions including free and open access courses delivered to thousands of learners through to more bespoke specialist skills delivered to digital champions using immersive teaching environments such as Jupyter Notebooks. | Our challenge for the future will be to carefully map the education to the competency frameworks developed to ensure the educational intervention is appropriately pitched to address the appropriate level of competency for a particular job role or profile. We are aware of the need to discern expert and more generalist competencies and also to align with specific personas and roles in healthcare. This refinement will help to further hone our educational interventions for the future. |
| Edirippulige 2022[25] | Australia, Sri Lanka, and Denmark | A qualitative study | Medical students | To explore medical students’ perceptions and expectations of digital health education and training (ET). | Most participants had no formal ET in digital health. Most participants (n = 43; 68%) expressed a willingness to learn about digital health as part of their medical programme. | Primarily, knowledge- and practice-related factors have motivated students to learn about digital health. The analysis of focus group data identified two superordinate themes: (a) drivers of digital health ET and (b) expectations relating to digital health ET. Students agreed that digital health is a relevant field for their future practice that should be taught as part of their regular curriculum. |
| El Kheir 2023[26] | Saudi Arabia | A cross-sectional study | Medical students | To assess the perception of undergraduate medical students on telemedicine training. | About 73% students think that the use of telemedicine for patient care will increase in the future, and 59.3% think that the medical students should be trained in telemedicine. Majority of the students (78%) opined that telemedicine training should be optional and 58% said it should be taught during the clinical phase of the undergraduate curriculum. The best telemedicine training course learning objectives medical students opined to be included were: telemedicine practical skills (70.2%), legal aspects of telemedicine practice (68.3%), and potential positive and/or negative use of telemedicine tools and methods (67.6%). Telemedicine skills students would like to learn how to effectively engage patients, knowledge about telemedicine regulations and the consequences of breaching them. | Medical students are aware of the importance of incorporating telemedicine training into the medical curriculum. Training these students is vital to ensure their competence as physicians in their future clinical careers, that is make them a digitally health-literate future workforce. |
| Erfani 2025[27] | UK and Finland | A cross-sectional study | Healthcare professionals | To identify the key characteristics influencing healthcare professionals’ digital health competence. | Higher levels of education, working in a hospital, more professional experience, and increased use of digital solutions at work or during free time were associated with higher digital health competence. Higher qualifications were linked to greater digital health competence across several factors, with postgraduate degrees increasing health-related counselling competence and undergraduate degrees enhancing work-related attitudes and evaluating digital solutions. Professionals with university qualifications and those working in hospital set- tings showed higher ICT competence, while more years of professional experience increased ethical competence in using digital solutions. | Digital health competence development should prioritise healthcare professionals with lower educational levels. Additionally, such initiatives should include supporting those who work in non-hospital settings, have less professional experience, and use digital solutions less frequently at work or in their free time. Professional experience increases ethical competence in using digital solutions. Continuous professional development interventions and organisational policies should consider these factors to improve healthcare professionals’ digital health competences. |
| Ersoy 2024[28] | Türkiye, Latvia, Portugal, Finland, and Sweden | Descriptive study | Faculty members in health sciences | To determine the digital competence level of faculty members who teach in the health sciences, empirically considering possible contextual aspects. | The results revealed that a majority of the faculty members have intermediate (integrator or expert) level of digital competence, which is described as being aware of the potential use of digital technology in teaching and having a personal repertoire for its use under various circumstances. Age, digital teaching experience, perception of work environment, and previous teaching experience in fully- or partially-online courses were identified as influencing factors for digital competence. Faculty members in health sciences were able to integrate digital technologies in their teaching practices. | Health education institutions may facilitate the use of digital technologies in teaching and learning environments. Moreover, institutions or stakeholders should consider that digital competence requires practice and experience in meaningfully-designed digital environments and tools. |
| Faihs 2022[29] | Germany | A cross-sectional online survey | Medical students | To investigate the views of medical students on digital healthcare | Male students more frequently reported keeping themselves informed about digital medicine outside of their studies across all clinical years of study. While female students self-assessed their knowledge in different fields of digital medicine as worse than their male peers in the first clinical years of study, no more gender differences could be found towards the final year. | Students of both genders showed a strong desire for further education on the topic of digital medicine. |
| Farooq 2024[30] | Pakistan | Cross-sectional analytical study | Medical sciences students | To assess digital health literacy among medical students. | Eight hundred ninety-one medical students, from first year to final year, participated in the study. The overall mean score for DHL was 63.5 (SD=8.82). Medical students achieved a score of 83.2% of total score in their operational abilities, and 82.3% proficiency in privacy protection, which were deemed highly desirable. Furthermore, they achieved a satisfactory level in navigation skill (76.0%), information searching (73.1%), adding content (71.0%), determining the significance of data (70.1%), and assessing data reliability (68.7%), based on the overall score. A significant relationship was observed between the performance level of DHL domains and gender with higher scores in males in all domains except protecting privacy, which was higher in females and clinical years students (p-value < 0.05) | The assessment of the DHL of medical students was deemed desirable. But certain obstacles were encountered in few domains of DHL i.e., data reliability, relevance determination, and content augmentation. It is imperative to elevate the level of DHL of medical students to harness the potential of digital technologies in enhancing healthcare. |
| Ghaedi 2024[31] | Iran | A correlational survey | Medical students | To investigate the perceived and performed eHealth literacy of medical sciences students, as well as the relationships between those literacies. | In general, over 70% of students perceived their skills of access and appraisal as good/very good, correlating with their estimated performance. Students perceived they were less confident in some appraisal skills (e.g., using information from the Internet to make health decisions) than others. Performed skills in information generation were mostly poor or very good; performed application skills generally good/very good. | The eHEALS score scales with actual skills (access and appraisal). Support is necessary for students in particular types of appraisal skills. |
| Gillissen 2022[32] | Germany | Mixed-methods study | Medical students | To explore attitudes of 2020 medical students’ generation towards various aspects of eHealth technologies with the focus on AI using an exploratory sequential mixed-method analysis. | Most students expressed positive attitudes towards digital applications in medicine. Students with a problem-based curriculum (PBC) in contrast to those with a science-based curriculum (SBC) and male undergraduate students think that AI solutions result in better diagnosis than those from physicians (*P* < 0.001). Male undergraduate students had the most positive view of AI (*P* < 0.002). Around 38% of the students felt ill-prepared and could not answer AI-related questions because digitization in medicine and AI are not a formal part of the medical curriculum. AI rating regarding the usefulness in diagnostics differed significantly between groups. | Higher emphasis in medical curriculum of digital solutions in patient care is postulated. |
| Hah 2019[33] | USA | Qualitative and Quantitative Study | Care providers | To explore care providers’ familiarity with using technology in different settings and their perceptions of telehealth-driven care performance to lay a foundation for the design of an effective telehealth education program. | A total of 109 advanced practice registered nurse students responded to the online survey and open-ended questionnaire. Most indicated that using telehealth technology enhances care performance, helps make their care tasks more effective, improves the quality of performing care tasks, and decreases error in communicating and sharing information with others. In addition, our qualitative analyses revealed that the students used the electronic health records technology primarily at work, combined with clinical decision support tools for medication and treatment management. Outside work, they primarily used video-text communication tools and were exposed to some telehealth technology in their education setting. Further, they believe that use of non-health technology helps them use health information technology to access health information, confirm their diagnoses, and ensure patient safety. | This research highlights the importance of identifying care providers’ existing experience of using technology to better design a telehealth technology education program. By focusing explicitly on the characteristics of care providers’ existing technology use in work, nonwork, and educational settings, we found a potential consistency between practice and education programs in care providers’ requirements for technology use, as well as areas of focus to complement their frequent use of nonhealth technologies that resemble telehealth technology. Health policymakers and practitioners need to provide compatible telehealth education programs tailored to the level of care providers’ technological familiarity in both their work and nonwork environments. |
| Hailegebreal 2022[34] | Ethiopia | A cross sectional study | Health science students | To assess utilization ICT and its associated factors among Arba Minch University College Medicine and Health Science students. | A total of 355 participants enrolled in the study, with a response rate of 98.34%. The percentage of students who used ICT was 55.77%. Regarding of field of study, health informatics students (84%) used the most ICT, while midwifery students (52%) used the least. Urban resident, ICT knowledge, having formal training of ICT, having IT in current course study, and had good IT skill revealed a significant and positive correlation with the use of ICT. | In the current study previous residence, ICT knowledge, having formal training, having IT in current courses, and IT skill were significantly associated with student ICT utilization. Therefore, the university should continue to invest in professional development in order to improve teaching and student performance, as well as provide the college with student-centered ICT computer labs to encourage students to use technology. |
| Hare 2022[35] | USA | Case report | Medical students | Share lessons and recommendations for medical schools and health systems looking to implement similar opportunities. | Developed and implemented a 5-tiered clinical informatics curriculum at the undergraduate medical education level: (1) a practical orientation to HIT for rising clerkship students; (2) an elective for junior students; (3) an elective for senior students; (4) a longitudinal area of concentration; and (5) a yearlong predoctoral fellowship in operational informatics at the health system level. | Most students found these offerings valuable for their training and professional development. |
| Hariyati 2024[36] | Indonesia and Taiwan | Cross-sectional study | Nursing Manager and Staff | To identify the digital literacy of nursing staff and nurse managers. | Participants showed an acceptable level of computer literacy, although their scores were below 80%. Their scores were unrelated to workplace, duration and type of technology used, or gender. Nursing informatics competencies correlated significantly with age and work experience (*P* = 0.001). Age correlated negatively with technical computer skills but positively with competence in the implementation of healthcare. There was a positive link with a nurse’s position and education level, indicating that nurses with more education acquire higher levels of informatics competencies than those with less education. Additionally, those in higher positions tended to possess more advanced digital competence. | Nurse managers play a significant role in training nursing staff members to enhance their informatics competencies. This study’s findings underscore the need to raise staff members’ informatics competencies by providing additional training. |
| Hassan 2022[37] | Pakistan | Survey study | Medical teachers | To ascertain medical instructors' usage of computers and the internet, as well as the factors that influence their use. | Of the total teachers participating, there were 90 (71.4%) male and 36 (28.6%) females. Participants were distributed based on their age as follows: 78 (61.9%) <35 years, 13 (8.1%) 36-50 years, and 35 (27.8%) >50 years. The incidence of traditional teaching, computerized projections, teaching through slides, and combination of traditional methods with slides were 19 (15.1%), 107 (84.9%), 68 (54%), and 32 (25.4%) respectively. Based on pattern of internet usage for teaching, the occurrence of collection of teaching materials without internet, collection of teaching materials using internet, access from desktop, and access from smartphones were 28 (22.2%), 88 (69.8%), 66 (52.4%), and 16 (12.7%) respectively. A significant statistical correlation exists between computer and Internet use among teachers of younger ages, with more frequent usage among teachers of younger ages. | The present study concluded that medical education has become increasingly computer-based and Internet-based. Furthermore, its use is more common among younger professors. However, the most noteworthy conclusion was that the majority students still choose chalkboard lecturing for better knowledge of the material. |
| Hautz 2020[38] | Switzerland | Overview | Medical deans | To gain an overview of the current training in digital competencies at Swiss universities. | All of the dean’s offices that were contacted participated in the survey. The topics on digitalization were all rated as relevant or very relevant. Our survey shows a heterogeneous picture in terms of implementing Principal Relevant Objectives and Framework for Integrative Learning and Education in Switzerland (PROFILES) and National Competency-based Catalogue of Learning Objective in Undergraduate Medicine (NKLM) content. A few universities have well-established educational approaches or even implemented curricula, but often these are still in development. Participants also mentioned factors that are necessary for successfully setting up and implementing curricula dealing with digitalization and factors that can impede such efforts. | The importance of acquiring digital competencies during medical study is known and recognized by all Swiss medical schools. Curricular integration varies in its progress and represents major challenges for the medical faculties. It is precisely the inclusion of students in such efforts that may be a potential response to this challenge. |
| Høium 2024[39] | Norway | A systematic review of reviews | Health students in higher education | To investigate what characterizes the use of digital technology in bachelor-level practice placements in health programs. | The analysis revealed three main topics that elucidate what characterises the use of digital technology in placements: Leveraging digital solutions for enhanced practical learning; Empowering student learning and confidence in practice placement; and the value of interactive collaboration. | The findings show that digital technology used in a structured pedagogical framework may support and enhance students learning in practice placement. However, further work needs to be done to keep up with the rapid development of digital technology in practice placement in health work environments. |
| Huang 2019[40] | China | A cross-sectional study | Health sciences postgraduates | To examine health sciences postgraduates’ attitudes and practices regarding curriculum for ICT use in global health research and training in China. | A total of 1065 participants successfully completed the questionnaires. More than 90% of the students have not had any training about ICT, three quarters have not taken an online course, and 31% of the students do not use ICT in their current research. More than 65% thought that, in an ICT research training curriculum, it was important to learn: ICT utilization related knowledge, ICT research methods/resources, knowledge of databases, ways of data use and acquisition, and informatics search methods (ICT users compared to non-users were more likely to agree to these learning components (all *P* < 0.05)). Many of the respondents used or planned to use mobile phones (80%), Internet (59%), use computer and WeChat (> 40%), and QQ (a popular chat tool in China) (30%) as ICT tools in research activities. ICT users compared to non-users were more likely to consider using ICT and/or biomedical informatics methods in decision-support or support for information seeking, healthcare delivering, academic research, data gathering, and facilitating collaboration (all *P* < 0.05). | The findings of this study showed that ICT utilization was very important to health sciences postgraduates for their research activities in China, but they lacked ICT-related training. The results suggested the need for specialized curriculum related to ICT use in global health research for health sciences postgraduates in China. |
| Hübner 2016[41] | USA | Survey study | Nursing students | Towards an International Framework for Recommendations of Core Competencies in Nursing and Inter-Professional Informatics: The TIGER Competency Synthesis Project | The TIGER Initiative deployed an international survey, with participation from 21 countries, to evaluate and prioritise a broad list of core competencies for nurses in five domains: 1) nursing management, 2) information technology (IT) management in nursing, 3) interprofessional coordination of care, 4) quality management, and 5) clinical nursing. Informatics core competencies were found highly important for all domains. In addition, this project compiled eight national cases studies from Austria, Finland, Germany, Ireland, New Zealand, the Philippines, Portugal, and Switzerland that reflected the country specific perspective. | These findings will lead us to an international framework of informatics recommendations. |
| Izumi 2016[42] | Japan | A post-workshop evaluation | Nursing professionals | to examine the effectiveness of behavior modification to resolve workplace issues identified by nursing professionals | After participating in these workshops, all nursing professionals demonstrated an increased ability to use nursing information and effectively resolve issues through behavior modification | The results suggest that the workshops provided head nurses with the opportunity to clarify their own role issues, and through proactive participation and discussion, share their experiences of improvements in the work process and activities that resolved workplace problems. As a result, they were able to make observations and generate measures that otherwise may not have been possible. Furthermore, establishing a second workshop to report on results seems to have given rise to behavior modification, as participants set their own goals to be achieved for the measures they planned at the first workshop and needed to achieve by the time of the second workshop. |
| Jacobs 2017[43] | USA | Survey study | Osteopathic medical students | To understand osteopathic medical students’ knowledge, attitudes, and behaviors regarding HIT and to identify factors that may be related to their readiness to use HIT. | Six hundred four students responded to at least 70% of the survey and were included in the analysis. Multivariate modeling successfully explained the 26% of variance in predicting students’ readiness to use HIT (F8,506=22.6, *P* < 0.001, R2=0.263). Greater self-efficacy, openness to change (in academic/work settings), favorable attitudes toward HIT use, mobile technology use, younger age, being male, and prior exposure to technology were associated with readiness to use HIT. | Understanding students’ level of HIT readiness may help guide medical education intervention efforts to better prepare future osteopathic physicians for HIT engagement and use. Innovative approaches to HIT education in medical school curricula that include biomedical informatics may be necessary. |
| Jarva 2024[44] | Finland and Sweden | Cross- sectional study | Healthcare professionals | To identify healthcare professionals' digital health competence profiles and explore associated factors to digital health competence in healthcare settings. | Analysis revealed three digital health competence profiles: A–high competence (n = 336), B–intermediate competence (n = 352) and C – low competence (n = 129). Between the profiles, digital health competence showed significant differences (*P* < 0.001). Recent graduation year, working in outpatient environments and leader or specialist position were associated with higher digital health competence. Organizational practices and the influence from colleagues improved competence in human-centred remote counselling, digital solutions as part of work, competence in utilizing and evaluating digital solutions and ethical competence. Support from management improved digital solutions as part of work and ethical competence. | Nursing and allied health professionals working in other than outpatient environments should be specifically acknowledged when digital health competence development initiatives are designed and targeted. The positive influence from colleagues could be harnessed by enhancing their involvement in digital health competence development methods such as orientation, mentoring or coaching. Additionally, managers should take a stronger role in supporting different areas of digital health competence. |
| Jidkov 2019[45] | UK | A mixed methods study | Postgraduate medical education | To assess health informatics (HI) training in UK postgraduate medical education, across all specialties, against international standards in the context of UK digital health initiatives (eg, Health Data Research UK, National Health Service Digital Academy and Global Digital Exemplars). | A framework of 50 HI competency domains was developed using 21 curricula from a scoping review, curricular content analysis and expert consultation. All 71 UK postgraduate medical curricula documents were mapped across 29 of 50 framework domains; that is, 21 domains were unrepresented. Curricula mapped between 0 (child and adolescent psychiatry and core surgical training) and 16 (chemical pathology and paediatric and perinatal pathology) of the 50 domains (median=7). Expert consultation found that HI competencies should be universal and integrated with existing competencies for UK clinicians and were under-represented in current curricula. Additional universal HI competencies were identified, including information governance and security and secondary use of data. | Postgraduate medical education in the UK neglects HI competencies set out by international standards. Key HI competencies need to be urgently integrated into training curricula to prepare doctors for work in increasingly digitised healthcare environments. |
| Jimenez 2020[46] | Singapore | Review | Healthcare professionals | To examine the broad literature on digital health competencies (DHCs) as it applies to Primary Care (PC)settings. | A total of 28 articles were included, most of them (54 %) published before 2005, These articles were primarily aimed at PC physicians or general practitioners, and focused on improving knowledge about information technologies and medical informatics, basic computer and information literacy, and optimal use of electronic medical records. We identified 17 DHC domains, and important knowledge gaps related to digital health education and curriculum integration, the need for evidence of the impact of services, and the importance of wider support for digital health. | Literature explicitly linking DHCs to PC was mostly published over a decade ago. There is a need for an updated and current set of DHCs for PC professionals to more consistently reap the benefits of digital technologies. This review identified key DHC domains and statements that may be used to guide on the development of a set of DHC for PC, and critical knowledge gaps and needs to be considered, such a DHC set may be used for curricula development and for ensuring that the essential DHC for PC are met at a clinical or organizational level and eventually improve health outcomes. |
| Jouparinejad 2020[47] | Iran | Interventional study | Critical care nurses | To evaluate the impact of a training program on Nursing informatics (NI) competency of critical care nurses. | All 60 participants completed the educational program and returned the completed questionnaire. Majority of participants in the intervention and control groups were female (83.30%), married nurses (70.90, 73.30%) aged 30–40 years (51.6, 35.5%). In the pretest stage, both intervention and control groups were competent in terms of the NI competency and its dimensions, and no significant difference was observed between them (*P* =0.65). However, in the posttest, the NI competency and its dimensions in the intervention group significantly increased with a large effect size compared with the control group (*P* = 0.001). This difference showed that the intervention group was proficient in the posttest stage. The highest mean difference in the intervention group was associated with the informatics literacy dimension and the lowest mean difference was associated with the informatics management skills dimension. | The improved scores of NI competency and its dimensions after using the training program implied the effectiveness of this method in enhancing the NI competency of nurses working in the critical care units. The application of the training program in diverse domains of nursing practice shows its high efficiency. The project is fundamental for improving nurses’ NI competency through continuous educational programs in Iran, other cultures and contexts. |
| kahouei 2015[48] | Iran | Descriptive study | Physicians, medical residents, and students | To investigate whether these interventions increased the use of evidence-based health information resources among physicians, medical residents and students. | A total 52.9% of physicians and 79.5% of medical residents and students always used patient data. 81.3% of physicians and 67.1% of medical residents and students reported using their own experiences, 26.5% of physicians and 16.9% of medical residents and students always used databases such as PubMed and MEDLINE for patient care. | Our results revealed that in spite of providing educational and technical infrastructures for accomplishment of research utilization in medical education, the study subjects often identified and used what they regarded as reliable and relevant information from sources that do not truly represent the best evidence that is available. |
| Kaihlanen 2024[49] | Finland | Mapping study | Healthcare professionals | To map and describe the existing continuing education in digital skills for healthcare professionals (HCPs) in European Union (EU) Member States. | The results show variations between countries in policy strategies, training organisation, and funding mechanisms. Educational institutions, employers, third parties, and national/regional authorities were found to be the main organisers of the digital skills training. Comprehensive accreditation systems seemed to be scarce, and practices also varied between countries. | The study highlights the importance of adopting a systematic approach to enhancing continuous professional development in digital skills, which would ensure that professionals have equitable access to education, resulting in consistent, quality patient care across countries and regions. The findings offer valuable insights for policymakers, educators, healthcare institutions, and professionals. |
| Keep 2021[50] | Australia | Review | Health, nursing, and medical university curricula | To understand how eHealth is taught at a major Australian university and the challenges and suggestions for integrating eHealth into allied health, nursing, and medical university curricula. | There was no evidence of a standardized approach to eHealth teaching across any of the health degrees at the university. Where eHealth content existed, it tended to focus on clinical applications rather than systems and policies, data analysis and knowledge creation, or system and technology implementation. Despite identifying numerous challenges to embedding eHealth in their subjects, unit coordinators expressed enthusiasm for eHealth teaching and were keen to adjust content and learning activities. | Explicit strategies are required to address how eHealth capabilities can be embedded across clinical health degrees. Unit coordinators require support, including access to relevant information, teaching resources, and curriculum mapping, which clearly articulates eHealth capabilities for students across their degrees. Degree-wide conversations and collaboration are required between professional bodes, clinical practice, and universities to overcome the practical and perceived challenges of integrating eHealth in health curricula. |
| Khamis 2018[51] | Saudi Arabia | A cross-sectional study | Undergraduate medical students | To compare IT skills, uses and preferences for education between traditional and PBL medical students’ | Most of the responding 176 students prefer mobile devices and moderate amount of IT in education. Fourth and fifth year students perceived high academic value of Google, YouTube and PubMed. More 4th year than 5th year students rated themselves as skilled in learning management system and Smartboard use. Most students rated faculty IT skills as effective. Students agreed that technology helps working faster (95.5%) and make learning creative (85.9%). | More integration of information literacy and IT training in medical curricula is needed to enhance better utilization of full features of IT resources available for learning and problem solving. National multi-institutional studies are recommended. |
| Kinny 2024[52] | Germany, Pakistan, and Singapore | Interventional study | Pharmacy students | To explore the feasibility of such an elective practical course on digital topics for student engagement and future implications. | Final-year pharmacy students' subjective assessments before and after the course depicted the increased knowledge and competence regarding analysing wearables data. | The increased availability and usage of wearables require pharmacy students to be prepared for related patient needs. The elective practical course on wearables and the health data they generate will enable future pharmacists to provide wearable counselling and guidance on wearables, such as CGM systems and blood pressure monitors, including their operation and functionality. Moreover, the interpretation of health data and subsequent recommendations for action will also enhance clinical skills and experience. Based upon our pilot project, a digital health course with practical elements appears to be feasible, provided that sufficient resources are made available. We also assume that an implemented course with a larger student cohort would be well received. Therefore, future educational research with larger study cohorts will be required to integrate digital health into pharmacy education. |
| Kröplin 2025[53] | Germany | A comparative study before and after the intervention | Medical students | To evaluate the impact of the “Digital Health” curriculum at our university on the perceptions of medical students regarding the relevance of digital health topics for their future professions and their self-assessed competence in these areas. | A total of 20 students participated, with 13 (65%) being women. In particular, data protection and information security were considered the most relevant topics both before and after the curriculum. Significant increases in perceived importance were observed for messenger apps (mean increase of 0.8 [SD 1.2]; *P* < 0.01). Regarding self-assessed competence significant development was observed on almost all topics, The greatest development was observed in robotics (mean increase of 1.8 [SD 1.2]: *P* < 0.001), open educational resources (mean increase of 1.7 [SD 1.5]: *P* < 0.001), and simulation-training (mean increase of 1.6 [SD 1.3]: *P* < 0.001). The gamification-based, robot-related teaching was predominantly rated suitable and very enioyable for the students | The results highlight the potential to integrate more innovative teaching techniques, such as gamification, augmented reality, virtual reality, and simulation training, into a technologically advanced health care environment. Finally, the overarching importance of artificial intelligence and digital health applications signals the need to further integrate them, given their potential in remote and personalized medicine. |
| Kuhn 2020[54] | Germany | A qualitative study | Medical students | To collect the students' perspective in order to incorporate the results into the iterative and agile development of the course concept, but also into the current national reform processes. Research was guided by the questions of the students' subjective learning success and the acceptance of the course concept. | The evaluation of the teaching events “Medicine in the digital age” comprises the deductively formed main categories of procedure, content, methods, learning success, learning experience and conclusion (see figure 2). Inductively developed subcategories were assigned to the deductive main categories during the evaluation process. With the help of these subcategories, the individual main categories can now be unlocked and defined in more detail. | The development of a digitisation strategy and its didactic is a relevant component of future planning for the curricular development of medical school, but also for continuous medical education. In the future, this will no longer be possible with a compulsory elective course but requires a comprehensive implementation in the curriculum. In this context, it must be critically reflected whether and how the course can be scaled. We are convinced that the practical and reflective parts must be represented in the form of internships for a maximum of 15 students. When developing these curricula, the high speed of the change process should also be taken into account and curricular adaptation in the sense of “agility by design” should be made possible right from the conception stage |
| Kühnel 2023[55] | Germany | A mixed methods study | Medical Students and Radiology Technician Trainees | To evaluate the self-assessment of ehealth literacy in terms of finding, using and critically evaluating health information and theoretical and practical hygiene awareness on a voluntary participation basis at the Jena University Hospital in 2022. | For the eHL, the respondents tended to have a positive self-assessment of finding, using and critically evaluating health information. The voluntary participants of the practical hand disinfection who had received self-training were able to achieve significantly better results (p = 0.0047), resulting in fewer wetting gaps in a subsequent performance with Visirub than those who had not received digital self-training. | Healthcare-related participants belonging to the “digital native” generation have above-average knowledge on HH and profit by digitally guided self-training. |
| Lan 2020[56] | Vietnam | Cross-sectional study | Medical students | To measure eHealth literacy of medical students at a university in central Vietnam and to examine factors influencing their skills. | The study found that the general mean score for eHealth literacy among participants is 27.03 (SD3.54). Factors influencing eHealth literacy are genders (p=0.001), training program (p-0.013), computer skills (p-0.031) and purpose of seeking and using medical information (p<0.001). | The eHealth literacy of medical students in the study setting is still limited. In order to improve these skills of students, the educators should have relevant teaching strategies that promote the interest and skills of students to locate and evaluate eHealth resources. |
| Lawrence 2024[57] | USA | A mixed methods study | Health professions trainees | Offer a model based on our growing understanding of “digital determinants of health”-the novel technological contexts and constructs that mediate an individual or community’s interactions with the health care system—and their intersections with care delivery, innovations, education, and equity. | Developed a “digital determinants of health” (DDoH) framework for understanding the intersections of health outcomes, technology, and training. | There is growing need to develop unified digital health education and training competencies for health professions students. Efforts to cultivate a workforce adept in digital health tools must prioritize understanding and mitigating the digital determinants of health that shape individuals’ interactions with health care technology. Using a DDoH framework in medical education—including not only didactic training but also hands-on skill building, as well as continuing education opportunities—can help guide robust educational programming and evaluation tools aimed at developing health professionals who understand and can competently use digital health tools to deliver care for diverse patients. |
| Le 2023[58] | Vietnam | Cross-sectional study | Medical students | To evaluates the reliability and validity of Vietnamese version of eHEALS and analyzed some factors affecting on eHEALS score among Hanoi Medical University students in Vietnam. | The total score of our subjects in the eHEALS was 30.34 ± 4.57. The results from Bartlett’s test, Kaiser- Meyer-Olkin (KMO) test, calculated Cronbach’s alpha coefficient and test–retest reliability were high. Poisson regression identified that eHEALS scores of participants was significantly associated with device, ongoing medical condition and trustworthiness of health information source (p < 0.05). | The Vietnamese version of eHEALS is a reliable and valid measure. Device, medical condition and trustworthiness of health information source are factors affecting on eHEALS score of students in Hanoi Medical University. |
| Lee 2024[59] | Korea | Review | Medical and nursing education | This review presents the current status of biomedical and health informatics education domestically and internationally and proposes recommendations for future development. | This review presents international recommendations for establishing education in biomedical and health informatics, as well as global examples at the undergraduate and graduate levels in medical and nursing education. It provides a thorough examination of the best practices, strategies, and competencies in informatics education. The review also assesses the current state of medical informatics and nursing informatics education in Korea. We highlight the challenges faced by academic institutions and conclude with a call to action for educators to enhance the preparation of professionals to effectively utilize technology in any healthcare setting. | To adapt to the digitalization of healthcare, systematic and continuous workforce development is essential. Future education should prioritize curriculum innovations and the establishment of integrated education programs, focusing not only on students but also on educators and all healthcare personnel in the field. Addressing these challenges requires collaboration among educational institutions, academic societies, government agencies, and international bodies dedicated to systematic and continuous workforce development. |
| Lee 2025[60] | Korea | Cross- sectional survey | Medical students and doctors | To assess the statistical literacy of medical students and doctors in South Korea by evaluating their comprehension of four statistical concepts: (a) single- event probability, (b) relative risk reduction, (c) positive predictive value and (d) 5- year survival rate. | The correct answer rates for basic numeracy questions were close to 100%. Regarding statistical literacy, 95.5% and 83.2% of the participants accurately understood single-event probability and relative risk reduction, respectively. However, only 49.3% and 49.2%of the participants accurately understood the positive predictive value and 5-year survival rate, respectively. The correct answer rates for the question about the5-year survival rate differed significantly between students (40.9%) and doctors (57.7%) (*P* < 0.001). There were no statistically significant differences in the correct answer rates for other questions, regardless of the student's grade level or the doctor's specialty. | Medical students and doctors have weaker statistical literacy than their basic numeracy. Therefore, it is essential to implement medical education and professional development programmes that focus on improving their statistical literacy. These programmes should specifically address measures of medical test accuracy and the distinction between a 5- year survival rate and mortality. |
| Lei 2016[61] | China and USA | Review | Medical informatics Course | A better understanding of the differences between medical informatics research and education in China and the discipline that emerged abroad will better inform Chinese scholars to develop right strategies to advance the field in China and help identify an appropriate means to collaborate more closely with medical informatics scholars globally. | Analyze the main problems that exist in the current disciplinary development in China related to medical informatics research and education and offer suggestions for future improvement | The evolution of medical informatics shows a strong and traditional concentration on medical library/bibliographic information rather than medical (hospital information or patient information) information, Misdirected-concentration, a lack of formal medical informatics trained teaching staff and mistakenly positioning medical informatics as an undergraduate discipline are some of the problems inhibiting the development of medical informatics in China. These lessons should be shared and learned for the global community. |
| Lekalakala-Mokgele 2023[62] | South Africa | Cross-sectional study | Nursing students | To assess eHealth literacy, pre-existing knowledge and the nursing students’ perceptions and attitudes towards eHealth. Aquantitative, descriptive, cross-sectional survey was used in this study. | Students in level four exhibit high scores of knowledge in the use of eLearning technology as compared to first-entry university nursing students. Nursing students used the internet frequently, especially to access social media and search for health and medical information for their study. Attitudes towards eHealth and technology were also found to be positive. | Digital literacy should be enhanced in the nursing education curriculum in other to further strengthen the knowledge and skills towards the use of eHealth and health technology among nursing students. |
| Li 2025[63] | China and Philippines | Cross-sectional study | Academic nurse educators | To explore the current status and associated factors of digital literacy among academic nurse educators. | The average digital literacy score was 125.27+ 11 .41, with the average scores for five dimensions from high to low by rank as follows: digital application (46.73 + 5.38), digital social responsibility (27.22+ 3.94), digital awareness (20.28 + 3.17), professional development (19.88 + 2.76), and digital technology knowledge and skills (11.16+ 2.03). Multiple linear regression analysis revealed that age, years of teaching experience, awareness of digital advancements, and use of digital technologies (e.g. ChatGPT) (all p<0.001) were significantly associated with the level of digital literacy among academic nurse educators | The findings suggest a need to design tailored digital education programs that address different age groups. For younger academic nurse educators, the focus should be on how to incorporate digital technology into their teaching practices to enhance educational diversity. For older academic nurse educators, training should prioritize building confidence in using digital tools and developing foundational digital skills to ensure they can effectively integrate technology into their instructional approaches. Therefore, supporting their needs and enhancing teaching competence towards sustainable nursing digital literacy. |
| Li 2024[64] | China | Observational study | Medical university teachers | To examine the level of the accumulation of digital literacy in medical university of China. | The results show that the accumulation of medical university teachers’ digital Literacy includes 4 types: linear accumulation, multi drive accumulation, parallel accumulation, and leading accumulation, of which multi drive accumulation and leading accumulation are the most conducive to the formation of medical university teachers’ digital literacy. In addition, our findings reveal that subjective initiative plays an important role in the accumulation of medical university teachers’ digital literacy. The accumulation of digital literacy is a dynamic and systematic process of the accumulation of individual life events of medical university teachers. | The guidance and encouragement of policies cannot meet the needs of medical university teachers for digital literacy improvement. The medical universities urgently need a long term and stable mechanism to accumulate digital literacy. It stimulates the occurrence of positive life events and the accumulation of advantages in the life course of medical university teachers, and promotes the transformation from the accumulated disadvantages of medical university teachers’ digital literacy to the accumulated advantages. |
| Lilly 2015[65] | USA | Cross-sectional study | Doctor of nursing practice curricula | To identify barriers to integration of IT content in the curriculum in doctor of nursing practice (DNP) programs, perceived IT competencies taught, and DNP faculty perception of competencies. | Barriers measured included lack of qualified faculty, faculty's limited knowledge or skills in IT, lack of interest, age, lack of time to learn IT, lack of time to use IT, too many work demands, lack of administrative vision, unclear expectations of faculty, lack of technical support to faculty, or lack of resources. Leading barriers to IT implementation were lack of time of faculty, too many other work demands of faculty, lack of resources dedicated to IT, and lack of qualified faculty to teach IT. Further research is necessary on doctorate-prepared faculty and on interventions to overcome these barriers is needed. | As primary users of IT in health care, nurses at all levels need to be well versed in informatics. The need for technology in all levels of the nursing curriculum is not new to the professional role of nursing. Nurses are required to collect, store, and retrieve data and information to guide nursing care. The need for nurses with these technological skills will contribute to improved quality and transformation of health care (IOM). The integration of IT competencies in nursing education is crucial to nursing education and the future of health care. Faculty support that fosters innovations and new models of practice will beneficial to promote and embrace IT in nursing education. Nursing administrators must be cognizant of the fact that faculty will need adequate time and resources to continue to promote these ever-changing IT skills. Further study in DNP students' perceptions of IT competencies, DNP faculty perceptions of IT competencies, and barriers of IT implementation in a DNP curriculum is necessary. |
| Liu 2024[66] | China | Cross-sectional study | Nursing students | To provide nursing educators with a refined evaluation model and targeted improvement strategies tailored to enhance undergraduate students’ informatics competencies. | According to the weighted results, "skill (C_2_)"is an important dimension with the highest weight ranking. The corresponding highest-ranking criteria for each dimension are "Knowing how to explain the information management strategies to ensure patient safety (C_12_), "Applying information technology tools to support patient safe management (wristband scanning to identify patients, patients' electronic orders, etc.) (C_21_)" and "Paying attention to the importance of information technology in clinical decision-making and preventing errors or facilitating patientcare coordination (C_32_)." In the case of the undergraduate nursing students' performance assessment, Student Ewas the best overall performer from the perspective of overall utility value. The remaining students ranked as follows. Student C > Student D > Student F > Student A > Student B. | This study model remedies the shortcomings of previous studies on evaluating undergraduate students’ informatics competency dimensions, provides a reference for nursing colleges to develop nursing informatics-related curriculum content, and helps train nursing instructors to assess and train specific students. The results indicate that information skills are an important factor in the development of nursing students’ informatics competencies; hence, nursing educators should prioritize the development of nursing students’ informatics competencies, followed by information knowledge and attitudes. |
| Livesay 2024[67] | Australia | Qualitative study | Clinical nurses | To identify gaps in the National Nursing and Midwifery Digital Health Capability Framework, based on the perspectives of clinical nurses, and in nurse educators’ confidence and knowledge to teach. | The results were categorized by and presented from the perspectives of nurse clinicians, nurse graduates, and nurse educators. Findings were listed against each of the framework capabilities, and omissions from the framework were identified. A series of statements and questions were formulated from the gap analysis to direct a future co-design process with nursing stakeholders to develop a digital health capability curriculum for nurse educators. | Further work to evaluate nursing digital health opportunities for nurse educators is indicated by the gaps identified in this study. |
| Lokmic-Tomkins 2022[68] | Australia | Prospective cohort study | Nursing students | To determine first-year pre-registration nursing students' perceived baseline digital literacy before their first clinical placement. | Participants engaged with digital technology early in life, with 49.75% students using some form of digital technology before ten years of age. Students reported the highest daily use of technology to search the internet for information (92%), online social networking (68.3%) and watching videos (67%). Most students expressed the least confidence in identifying different types of portable storage devices (24.1% Master's students; 41.7% Bachelor's students), describing the advantages of a digital camera (39.3% Master's students; 48.3% Bachelor's students), and totaling numbers in spreadsheets (22.8% Masters students; 48.3% Bachelor's students). No statistical differences were observed between the two universities or the two cohorts in terms of perceived confidence in using technology and software applications to support their learning. Interestingly, 24.7% of participants expressed high confidence in using electronic medical records without prior training, which may reflect positive attitude towards engaging with unknown digital technologies. | Nursing students are frequent internet and social media users. However, despite positive attitudes to digital technology and widespread presence of digital technology in students' lives, deficits in students' confidence in using digital technology and software required for learning persist. Targeted digital literacy education interventions are needed as part of foundational nursing studies to improve nursing students' baseline digital literacy before commencing clinical placement. These should be scaffolded across the program to ensure an effective transition to nursing practice in evolving digitally-driven healthcare environments. |
| Lokmic-Tomkins 2024[69] | Australia | Qualitative study | Health informatics curricula | To report findings from an international participatory workshop exploring preregistration informatics implementation experiences. | Fourteen participants represented seven countries and a range of educational experiences. Four themes emerged: 1) Design: scaffolding digital health and technology capabilities; 2) Development: interprofessional experience of and engagement with digital health technology capabilities; 3) implementation strategies; and 4) Evaluation: multifaceted, multi-stakeholder evaluation of curricula. These themes were used to propose an implementation framework. | Workshop findings emphasise global challenges in integrating health informatics into curricula. While course development approaches may appear linear, the learner-centred implementation framework based on workshop findings, advocates for a more cyclical approach. Iterative evaluation involving stakeholders, such as health services, will ensure that health professional education is progressive and innovative. |
| Lungeanu 2022[70] | Romania | Retrospective study | Medical students | Appraised the existing biomedical informatics (BMI) and biostatistics courses taught to students enrolled in a six-year medical program. | Appreciation of the EM course was high, with a median (IQR) score of 9 (7–10) on a scale from 1 to 10. The overall scores for the BMI and biostatistics were 7 (5–9) and 8 (5–9), respectively. These latter scores were strongly correlated (Spearman correlation coefficient R = 0.869, *P* < 0.001). We found no correlation between measured and self-assessed knowledge of data science (R = 0.107, *P* = 0.246), but the latter was fairly and significantly correlated with the perceived usefulness of the courses. | The keystone of this different perception of EM versus data science was the courses’ apparent value to the medical profession. The following conclusions could be drawn: (a) objective assessments of residual knowledge of the basics of data science do not necessarily correlate with the students’ subjective appraisal and opinion of the field or courses; (b)medical students need to see the explicit connection between interdisciplinary or complementary courses and the medical profession; and (c) courses on information technology and data science would better suit a distributed approach across the medical curriculum. |
| Machleid 2020[71] | Belgium, Lithuania, Spain and Germany | Survey study | Medical students | To assess European medical students’ perceived knowledge and opinions toward digital health, the status of digital health implementation in medical education, and the students’ most pressing needs. | The survey received a total of 451 responses from 39 European countries, and there were respondents for every year of medical studies. The majority of respondents saw advantages in the use of digital health. While 40.6% (183/451) felt prepared to work in a digitized health care system, more than half (240/451, 53.2%) evaluated their eHealth skills as poor or very poor. Medical students considered lack of education to be the reason for this, with 84.9% (383/451) agreeing or strongly agreeing that more digital health education should be implemented in the medical curriculum. Students demanded introductory and specific eHealth courses covering data management, ethical aspects, legal frameworks, research and entrepreneurial opportunities, role in public health and health systems, communication skills, and practical training. The emphasis lay on tailoring learning to future job requirements and interprofessional education. | This study shows a lack of digital health-related formats in medical education and a perceived lack of digital health literacy among European medical students. Our findings indicate a gap between the willingness of medical students to take an active role by becoming key players in the digital transformation of health care and the education that they receive through their faculties. |
| Mannevaara 2024[72] | Finland | Scoping review | Teachers Students Education planners Study advisors Executive teams Different teams related to the teaching area | to explore how the teaching of education in HI has been arranged | The results highlight three key competencies: documentation and communication, management, and understanding of health information technology. It underlines a blended teaching method to improve the competencies of healthcare professionals, graduates, undergraduates, and suggests adding active interactions, multi-professional interactions, and hands-on skills. | This study highlights the importance of adapting to changes in healthcare, improving HI competencies in healthcare, and fostering positive digital experiences. It underlined the need for practical training, in theory and hands-on sessions, including key competencies in documentation and communication, management and health information systems. |
| Marsilio 2024[73] | Italy | Cross-sectional study | Medical students and residents | To identify gaps in current medical education programs by examining two primary aspects: (1) technical readiness (encompassing general and health-related digital competencies) and (2) behavioural readiness, which includes prior experiences and future intentions related to telemedicine education and implementation among medical students and residents. | The most commonly owned technologies were laptops and smartphones, with smartphones perceived as the easiest to use, while desktop computers presented more challenges. Approximately 38% of respondents expressed apprehension about applying digital health information in decision-making processes. There was a significant lack of both personal and academic experience, with only 16% of students and residents having used telemedicine in a university setting. Despite this, 83% of participants expressed a desire for training in telemedicine, and 81% were open to experimenting with it during their academic journey. Moreover, 76% of respondents expressed interest in incorporating telemedicine into their future clinical practice. | This study highlights the need for medical students and residents to receive specific education in digital health and telemedicine. Introducing curricula and courses in this domain is critical to addressing the challenges of digital healthcare. |
| Martinez-Ulloa 2024[74] | Chil and Spain | Cross-sectional study | Curricula of medical and rehabilitation science courses | To analyze the degree of association between university education models and the development of health literacy for health and rehabilitation students. | The results reveal a significant difference between students who participated in an electronic health skills training pro-gram compared to those who were not part of such a program in their university education. | The implementation of digital health literacy programs in higher education increases the ability to search for and evaluate the quality of health information online. |
| Mather 2022[75] | Australia | Cross-sectional study | Undergraduate health profession students | To explore the eHealth literacy of undergraduate health profession students to inform undergraduate curriculum development to promote work-readiness. | Students generally had good knowledge of health (Scale 2); however, they had concerns over the security of online health data (Scale 4). There were also significant differences in age and ownership of digital devices. Students who were younger reported higher scores across all seven eHLQ scales than older students. | This research provided an understanding of eHealth literacy of health profession students and revealed sub-groups that have lower eHealth literacy, suggesting that digital health skills should be integrated into university curriculums, especially related to practice-based digital applications with special focus to address privacy and security concerns. Preparation of health profession students so they can efficiently address their own needs, and the needs of others, is recommended to minimise the digital divide within health and social care environments. |
| Mesko 2015[76] | Hungary | Interventional study | Medical curriculum | To design a new e-learning-based curriculum and test it with medical students. | Over a 3-year period, 932 students completed the course. The course did not increase the number of hours spent online but aimed at making that time more efficient and useful. Based on the responses of students, they found the information provided by the curriculum useful for their studies and future practices. | A well-designed course, improved by constant evaluation-based feedback, can be suitable for preparing students for the massive use of the Internet, social media platforms, and digital technologies. New approaches must be applied in modern medical education in order to teach students new skills. Such curriculums that put emphasis on reaching students on the online channels they use in their studies and everyday lives introduce them to the world of empowered patients and prepare them to deal with the digital world. |
| Nault 2025[77] | USA | Observational study | Graduate medical | To assess feasibility of using eHL and FEBM-IH assessments in graduate coursework and revalidate the revised FEBM to integrative health. | Outcome completion rates suggest the FEBM-IH and eHL assessment tools are feasible to include in online courses, with 68.9% (102/148) eligible participants joining and 76.5% (78/102) completing all questions in all measures. The FEBM-IH demonstrated excellent assessor agreement (kappa = 0.97, *P* < 0.001), high internal consistency (a = 0.799), and acceptable item discrimination (0.26–0.68). Median self-perceived eHL scores increased from 30/40 to 33/40 points by course’s end, suggesting some increase in eHL. | Tools were feasible to integrate; FEBM-IH maintains acceptable validity; and further exploration of the relationship between EBM and eHL is warranted. |
| Nazeha 2020[78] | Singapore, Netherlands, and UK | Scoping review of the literature | Health care workers | To identify and study existing digital health competency frameworks for health care workers and provide recommendations for future digital health training initiatives and framework development. | In total, 30 frameworks were included in this review, a majority of which aimed at nurses, originated from high-income countries, were published since 2016, and were developed via literature reviews, followed by expert consultations. The thematic analysis uncovered 28 digital health competency domains across the included frameworks. The most prevalent domains pertained to basic information technology literacy, health information management, digital communication, ethical, legal, or regulatory requirements, and data privacy and security. The Health Information Technology Competencies framework was found to be the most comprehensive framework, as it presented 21 out of the 28 identified domains, had the highest number of competencies, and targeted a wide variety of health care workers. | Digital health training initiatives should focus on competencies relevant to a particular health care worker group, role, level of seniority, and setting. The findings from this review can inform and guide digital health training initiatives. The most prevalent competency domains identified represent essential interprofessional competencies to be incorporated into health care workers’ training. Digital health frameworks should be regularly updated with novel digital health technologies, be applicable to low- and middle-income countries, and include overlooked health care worker groups such as allied health professionals. |
| Nguyen 2022[79] | Vietnam, USA, and Singapore | Cross-sectional study | Medical students and health care professionals. | To investigate perceptions and practices regarding eHealth and their associated factors among medical students and health care professionals. | In total. 61.6%% (322/523) of participants used eHealth tools in clinical practices, with moderate levels of eHealth literacy. The score for the perceived benefits of eHealth tools was low. The most common barrier for eHealth utilization was human resources for IT (240/523, 45.9%), followed by security and risk control capacity (226/523, 43.2%) and no training in eHealth application (223/523, 42.6%). Age, eHealth literacy, and the use of the internet for updating medical knowledge were positively associated with using eHealth tools in clinical practices. | eHealth tools were moderately used in clinical practices, and the benefits of eHealth were underestimated among health care professionals and medical students in Vietnam. Renovating the current medical education curriculum to integrate eHealth principles should be required to equip health care professionals and medical students with essential skills for rapid digital transformation. |
| O'Brien 2023[80] | UK, Zimbabwe, Luxembourg, Ghana, Cameroon, Italy, Ethiopia, Netherlands, Kenya, South Africa, and USA | Qualitative study | Primary care professionals | To capture the multidisciplinary experiences of primary care professionals using DHTs to explore the strengths and weaknesses, as well as opportunities and threats, regarding the implementation and use of DHTs in SSA primary care settings. | A total of 33 participants participated in the study (n=13 and n=23 in the interviews and in focus groups, respectively; n=3 participants participated in both). The strengths of using DHTs ranged from improving access to care, supporting the continuity of care, and increasing care satisfaction and trust to greater collaboration, enabling safer decision-making, and hastening progress toward universal health coverage. Weaknesses included poor digital literacy, health inequalities, lack of human resources, inadequate training, lack of basic infrastructure and equipment, and poor coordination when implementing DHTs. DHTs were perceived as an opportunity to improve patient digital literacy, increase equity, promote more patient-centric design in upcoming DHTs, streamline expenditure, and provide a means to learn international best practices. Threats identified include the lack of buy-in from both patients and providers, insufficient human resources and local capacity, inadequate governmental support, overly restrictive regulations, and a lack of focus on cybersecurity and data protection. | The research highlights the complex challenges of implementing DHTs in the SSA context as a fast-moving health delivery modality, as well as the need for multistakeholder involvement. Future research should explore the nuances of these findings across different technologies and settings in the SSA region and implications on health and health care equity, capitalizing on mixed-methods research, including the use of real-world quantitative data to understand patient health needs. The promise of digital health will only be realized when informed by studies that incorporate patient perspective at every stage of the research cycle. |
| Ødegaard 2024[81] | Norway | Qualitative in-depth interviews | Physiotherapy teachers | To investigate physiotherapy teachers’ attitudes toward and experiences with digital education and what the teachers’ considered prerequisites to a digital transformation of teaching and learning in physiotherapy. | The findings illuminate teachers’ attitudes toward and experiences with digital education and their views on prerequisites to a digital transformation of teaching and learning in physiotherapy education, presented as four themes: 1) skepticism toward digital education; 2) digital technology as a tool to support the established teaching practice; 3) longing for teacher collaboration; and 4) calling for time to plan and learn, and significant academic leadership. | This study shows how physiotherapy teachers are skeptical about digital education, primarily viewing it as a threat to established teaching practices. Taken together, the findings demonstrate a potential for digital transformation in physiotherapy education, which can be released by informing the current teaching practices with evidence from research showing how use of digital technology can improve teaching and learning in physiotherapy education. |
| Ogundiya 2024[82] | UK | Narrative review | Medical students, physicians in training or continuing professional development, nurses, paramedics, and patients | To outline and discuss the developments that have taken place in digital medical education across the defined time frame. In addition, evidence for potential opportunities and challenges facing digital medical education in the near future was collated for analysis. | Evidence of the significant steps in the development of digital medical education in the past 25 years was presented and analyzed in terms of application, impact, and implications for the future. The results were grouped into the following themes for discussion: learning management systems; telemedicine (in digital medical education); mobile health; big data analytics; the metaverse, augmented reality, and virtual reality; the COVID-19 pandemic; artificial intelligence; and ethics and cybersecurity. | Major changes and developments in digital medical education have occurred from around the start of the new millennium. Key steps in this journey include technical developments in teleconferencing and learning management systems, along with a marked increase in mobile device use for accessing learning over this time. While the pace of evolution in digital medical education accelerated during the COVID-19 pandemic, further rapid progress has continued since the resolution of the pandemic. Many of these changes are currently being widely used in health education and other fields, such as augmented reality, virtual reality, and artificial intelligence, providing significant future potential. The opportunities these technologies offer must be balanced against the associated challenges in areas such as cybersecurity, the integrity of web-based assessments, ethics, and issues of digital privacy to ensure that digital medical education continues to thrive in the future. |
| Oo 2021[83] | Myanmar | Cross-sectional study | Health professionals | To assess the ICT literacy, knowledge, and readiness for EMRs adoption among health professionals in a tertiary hospital, Myanmar. | The prevalence of high ICT literacy and knowledge on EMRs among health professionals were 20.3% and 24.6% respectively. The factors associated with ICT literacy were professional, education, duration of service, and reported English language skills. Duration of service was associated with knowledge on EMRs. The overall readiness was 54.2% (core readiness 59.3% and engagement readiness 61.9%), and postgraduate [Adjusted Odds Ratio (AOR): 7.32, 95% Confidence Interval (CI): 2.26–23.68] and knowledge on EMRs (AOR: 1.27, 95% CI: 1.13–1.43) were the factors associated with overall readiness for EMRs adoption. | Expanding infrastructure and provision of ICT development training are crucial for the improvement of ICT literacy. EMRs training program enabling hands-on experience should be implemented for improvement of knowledge on EMRs. In general, the overall readiness for EMRs adoption was found to be moderate. Enhancing the establishment of comprehensive on-the-job training and contextualization of curriculum in EMRs training pro- gram are recommended to improve the health professionals’ readiness for EMRs adoption. |
| Pajari 2022[84] | Finland | Cross-sectional Study | Health sciences educators | To assess the appearance of digital competence in the work of Finnish health sciences educators and to determine whether educators' background factors are related to the areas of digital competence appearance. | Health sciences educators had participated in continuing education to develop their expertise and used a variety of digital methods and materials. Educators need more competence to improve healthcare students' ability to use digital technology. In the area of Teaching and Learning, educators younger than 40 years rated the appearance of digital competence as better than did those between the ages of 40 and 49 years. | In the future, health sciences educators' basic and continuing education could take into account the competence requirements for digital competence, and educators' expertise must be increased in areas where digital competence does not appear strong. |
| Panhwar 2021[85] | Pakistan | Cross-sectional Study | Medical and dental undergraduate students | To assess the usage of Information Technology (IT) and other types of software by medical and dental students. | Results: Out of 100 students, 62 were females and 38 males. Mean age was 21.749+1.54 years (range 19-24). 0verall result showed that 65%students spent limited hours in a day for internet access. Social networking was used by 40%students,53% mostly used Facebook sites and60% used YouTube medical videos for medical information. | Internet was used by most of the medical and dental students, but they were not fully utilizing the E resources towards the learning process. Medical students reported low level of internet use, this due to the limited availability of the internet access facilities and provision of technical skill at their university. |
| Park 2020[86] | Canada | Mixed method study | Pharmacy students | To explore the current state of pharmacy students’ self-rated digital health literacy in British Columbia, Canada, and seeks to identify future opportunities for technology training in pharmacy education and in practice. | A total of 30 pharmacy students completed the eHEALs survey and 5 completed interviews. Most participants were 2yeastudents (50%, were 25 years and younger (80%, and female (87%). Ranking of digital health literacy was lower than expected with participants stating they know what (87%), where (87%) and how to find (77%) health resources on the internet. Even less students (77%) rated that they have the skills to evaluate the health resources that they find on the Internet and only 53% fet confident in using information from the internet to make health decisions. Most students mentioned that they had imited technology related training at school and would like more training opportunities throughout their program and connect what they have learned at school to their practice. | These results expose significant and surprising gaps in student understanding of technology despite modifications seen in the entry-to-practice PharmD curriculum. Regional differences and digital health literacy of practicing pharmacists are areas that require better understanding and hold significant impact as practice evolves. |
| Park 2019[87] | Korea | Review | Medical students | To provide a succinct summary of the current state of AI from a medical viewpoint and suggest what medical students should do to prepare for the era of AI in medicine. | Artificial intelligence (AI) is expected to affect various fields of medicine substantially and has the potential to improve many aspects of healthcare. However, AI has been creating much hype, too. In applying AI technology to patients, medical professionals should be able to resolve any anxiety, confusion, and questions that patients and the public may have. Also, they are responsible for ensuring that AI be- comes a technology beneficial for patient care. These make the acquisition of sound knowledge and experience about AI a task of high importance for medical students. Preparing for AI does not merely mean learning information technology such as computer program- ming. One should acquire sufficient knowledge of basic and clinical medicines, data science, biostatistics, and evidence-based medicine. As a medical student, one should not passively accept stories related to AI in medicine in the media and on the Internet. Medical students should try to develop abilities to distinguish correct information from hype and spin and even capabilities to create thoroughly validated, trustworthy information for patients and the public. | AI is expected to affect various fields of medicine substantially and, if properly designed and used, has the potential to reinforce many weaknesses in current medical practice and improve many aspects of healthcare. Healthcare professionals are responsible for ensuring that AI becomes a technology beneficial for patient care. Medical students should develop abilities to distinguish correct information about AI from hype and spin and even capabilities to create thoroughly validated, trustworthy information for patients and the public to prepare for the era of AI in medicine. |
| Pokharel 2016[88] | Australia | Cross-sectional study | Medical and Dental Interns | To measure the eHealth literacy skills among the medical and dental interns of the B P Koirala Institute of Health Sciences (BPKIHS), Nepal. | A quarter of interns are not sure about the usefulness of internet resources for health. The interns have limited knowledge about right resources in the internet. They are not equipped to tell useful resources from the not-useful ones in the internet. | There is a need to pay attention to the eHealth Literacy needs of the interns. Further research may be needed to generate evidence on what interventions may be specific to meet the ehealth literacy needs of the medical and dental interns. |
| Poncette 2020[89] | Germany | Mixed methods study | Medical students | To introduce digital health as a curricular module at a German medical school and to identify undergraduate medical competencies in digital health and their suitable teaching methods. | The module received overall positive feedback from both students and lecturers who expressed the need for further digital health education and stated that the field is very important for clinical care and is underrepresented in the current medical curriculum. We extracted a detailed overview of digital health competencies, skills, and knowledge to teach the students from the expert interviews. They also contained suggestions for teaching methods and statements supporting the urgency of the implementation of digital health education in the mandatory curriculum. | An elective class seems to be a suitable format for the timely introduction of digital health education. However, a longitudinal implementation in the mandatory curriculum should be the goal. Beyond training future physicians in digital skills and teaching them digital health’s ethical, legal, and social implications, the experience-based development of a critical digital health mindset with openness to innovation and the ability to assess ever-changing health technologies through a broad transdisciplinary approach to translate research into clinical routine seem more important. Therefore, the teaching of digital health should be as practice-based as possible and involve the educational cooperation of different institutions and academic disciplines. |
| Potter 2025[90] | UK | Umbrella Review and Modified Delphi Method Study | Health professions education | To define the gaps in the evidence for the efficacy of digital education and to identify priority areas where future research has the potential to contribute to our understanding and use of digital education. | A total of 8857 potentially relevant papers were identified. Using the PRISMA (Prefered Reporting items for Systematic Reviews and Meta-Analyses) methodology, we included 217 papers for full review. All papers were either systematic reviews or meta-analyses. A total of 151 research recommendations were extracted from the 217 papers. These were analyzed, recategorized and consolidated to create a final list of 63 questions. From these, a modified Delphi process with 42 experts was used to produce the top-five rated research priorities: (1) How do we measure the learning transfer from digital education into the clinical setting?(2) How can we optimize the use of artificial intelligence, machine learning, and deep learning to facilitate education and training'(3) What are the methodological requirements for high-quality rigorous studies assessing the outcomes of digital health education'(4) How does the design of digital education interventions (eg, format and modality) in health professionals' education and training curriculum affect learning outcomes? and (5) How should learning outcomes in the field of health professions’ digital education be defined and standardized? | This review provides a prioritized list of research gaps in digital education in health care, which will be of use to researchers, educators, education providers, and funding agencies. Additional proposals are discussed regarding the next steps needed to advance this agenda, aiming to promote meaningful and practical research on the use of digital technologies and drive excellence in health care education. |
| Quek 2025[91] | UK | Review | Undergraduate Surgical students | To elucidate the current utilisation of digital technologies in undergraduate surgical education by addressing the research question: 'How are digital technologies currently being utilised in undergraduate surgical education to meet surgical learning outcomes'. | First, the use of digital tools in surgical education has been steadily increasing over the past few decades, with the COVID-19 pandemic accelerating the integration of technology into surgical education. Second, this review also highlighted the key role of anatomy within surgical education, with most included studies reporting the use of digital technologies to enhance anatomy teaching. Finally, this review provided an overview of various digital tools used in surgical education and their associated user experiences. Overall, most studies indicated that digital technologies are well-received by students, with many advocating for their continued use in supplementing surgical education even beyond the pandemic. | This review provides a crucial foundation for understanding the evolving role of digital innovations in shaping undergraduate surgical education. To enhance undergraduate surgical education, integrating appropriate digital learning tools can provide more learner-centred and personalised learning experiences. Educators must recognise that there is no ‘one-size-fits-all’ approach, and a flexible multimodal strategy is necessary to meet diverse learning needs. As technology continues to evolve and its role in education grows, this review offers valuable insights into the current use of digital tools in surgical education, highlighting opportunities for improvement and innovation to further enhance undergraduate surgical experience. |
| Raghunathan 2023[92] | Australia | Cross-sectional survey | Nursing students | To explore undergraduate nursing students’ self-perceptions of informatics competence, set within a larger research project. | Participants’ perceived overall mean informatics competency was at the level of somewhat competent, with only 40.84% (n = 58) at the level of competent. The highest mean value was in foundational information and communication skills and the lowest in information and knowledge management. Formal informatics education within curriculum was limited and lacked uniformity, as was prior exposure to important simulated informatics tools in preparation for practice. Factors including academic year level, computer experience and previous experience using clinical systems had a significant impact on participants’ perceived informatics competency. | Even though informatics competence is vital for clinical practice, with technology becoming pervasive within healthcare, nursing students’ preparedness for digital health was sub-optimal. There were gaps in students’ critical informatics practice knowledge with implications for work readiness of future graduates and nurse education practice. |
| Rathnayake 2019[93] | Sri Lanka | Cross-sectional survey | Nursing students | To assess eHealth literacy skills and associated factors among nursing students. | The sample consisted of 440 nursing students (420 females and 20 males). The mean eHealth literacy score was 28.02 (SD + 4.60). Nearly half of the respondents (49.4%) reported inadequate eHealth literacy skills, The respondents reported comparatively poor skills in differentiating high-quality health resources from low-quality health resources on the internet and the ability to use information from the internet to make health decisions. The majority viewed that including information technology (IT) as a subject into the nursing curriculum was very (50.7%) or absolutely (33.6%) important. The influencing factors of eHealth literacy skills of nursing students were self-rated internet skills (*P* =< 0.001), perception towards using the internet in health decision making (*P* = 0.009) and using the internet to access health resources (*P* = 0.001). | Half of the nursing students have inadequate eHealth literacy skills, particularly skills in identifying trusted health resources and using this information in health decision making indicating the need for improving eHealth literacy skills among nursing students. A positive attitude towards the internet has a significant role in developing eHealth literacy skills. Improving competencies in eHealth literacy skills of nursing students is essential. introducing these concepts into curricula, planning target interventions, and enhancing IT facilities within the educational environment are essential. |
| Ren 2025[94] | China | Cross-sectional survey | Pediatric students and pediatric healthcare workers | To investigate digital perceptions and competencies among medical students in pediatrics and pediatric healthcare workers in China. | The study included 518 valid questionnaires: 199 medical students in pediatrics and 319 pediatric healthcare workers all five themes, pediatric healthcare workers and pediatric students had a mean score higher than 3.0, and the former scored higher. There was no significant difference in the digital competency in pediaricians (3.91) compared to pediatric students'3.82) (*P* > 0.05). Multivariable analysis revealed that gender and education level were related with digital competency, with male medical students (3.94 vs. 3.75, *P* = 0.021) and those with higher levels of education (3.99 vs. 3.75, *P* = 0.030) having better performance. In addition, medical students in pediatric surgery scored higher than those who majored in internal pediatrics (4.11 vs.3.76. P=0.017). | Pediatric students and pediatric healthcare workers had a good perception on information of digital technology in the medical field, but had limited ability in digital use and collaboration, digital capacity development, and digital content creation. Better medical education and training strategies should be developed for potential challenges in the coming artificial intelligence era. |
| Rigas 2025[95] | Greece | Cross-sectional survey | Researchers and educators | Outline the profiles and the expertise of the workshop’s participants, the main gaps in knowledge and competencies, the governance and legislation issues and the need for certification in the digital health domain. | To assess computer literacy (CL) needs of medical sciences students. | The workshop results reveal a diverse participant profile, primarily comprising researchers and educators, with a strong representation from Europe and extensive experience in healthcare. Despite being familiar with digital health technologies, participants highlighted significant gaps in healthcare education, especially regarding systematic training in digital health, legal and ethical considerations, and governance of data. The challenges faced in implementing digital health technologies, such as legislative barriers, limited datasets, resistance, and slow adoption of standards, underscore systemic issues that hinder progress. Furthermore, difficulties with terminology, multilingualism, and data transformation emphasize the need for harmonization and practical solutions in the field. Notably, the lack of engagement in certification programs, both as trainers and trainees, highlights an opportunity for improvement in fostering structured learning pathways and professional development in digital health. This discussion underscores the pressing need for enhanced educational frameworks and collaborative efforts to overcome barriers in digital health adoption. |
| Robabi 2015[96] | Iran | Cross-sectional survey | Medicine, dentistry, paramedics, health, rehabilitation, nursing and midwifery | To assess computer literacy (CL) needs of medical sciences students. | The results showed that the 77.1% had personal computer. The total mean of students’ computer literacy around six domains was 141.9+49.5 out of 240. The most familiarity with computers was the ability to it in internet (29.0+11.4) and the lowest was familiarity and using ability of hard ware (17.5+10.6). There was a significant relationship between passing the computer lesson (P-0.001), passing Computer course (*P* = 0.05) and having personal computer (*P* = 0.001) with the mean of computer literacy. | In sum, the medical sciences students’ familiarity with computer literacy was not satisfactory and they had not appropriate familiarity with computer literacy skills. The researchers suggest the officials and in-charges to plan educational program for improving computer literacy skills in medical sciences students. Keywords: computer literacy, medical sciences, students |
| Rohan 2024[97] | USA | Qualitative research | Graduate Medical students | To assess the inclusion of CI topics for all medical residency specialties to assess the relative levels of CI knowledge expected by graduates. | All specialties’ requirements contained at least five different keywords, with the total count ranging from 25 to 42 (mean: 32.00; standard deviation: 5.09). Pathology contained the highest counts with 42, followed by internal medicine and family medicine with 41 each. Pathology included the most distinct keywords (11). The most common keywords were “leadership” (62%) and “electronic health record” (10%). There were no specific mentions of several keywords—including “analytics,” “artificial intelligence,” and “machine learning”—within any program requirements. Although the ACGME Residency Program Requirements state that residents must demonstrate competence in using information technology to optimize learning, the extent is not fully specified; only 10 programs mention the keyword “information technology” within their specialty guidelines. | The integration of Clinical informatics (Cl) education varies across specialties and may be even more variable across programs. Our study highlights potential opportunities for further standardization and integration of CI into resident curriculum requirements in order to better prepare future physician workforces for a changing medical landscape. We encourage educators, residency review committees, and national specialty organizations to consider further exploring the incorporation of CI content into residency training program requirements. |
| Rowland 2025[98] | USA | Qualitative research | Graduate Medical students | to inform U.S. Graduate Medical Education (GME) program leaders of the necessary requirements for implementation of a similar program at their institution. | The curriculum has yielded 13 graduates from both internal medicine (11, 85%) and pediatrics (2, 15%) whose projects have spanned acute and ambulatory care and multiple specialties. Projects have included clinical decision support tools, of which some will be leveraged as substrate in applications seeking extramural funding. Graduates have gone on to CI board certification and fellowship, as well as several other specialties, creating a distributed network of clinicians with specialized experience in applied CI. | An informatics curriculum at the GME level may increase matriculation to CI fellowship and more broadly increase development of the CI workforce through building a cadre of physicians with health information technology expertise across specialties without formal CI board certification. We offer an example of a longitudinal pathway, which is rooted in aLHS principles. The pathway requires a dedicated multidisciplinary team and departmental and information technology leadership support. |
| Saaiq 2024[99] | Pakistan | Qualitative study | Medical and dental teachers | To explore the core competencies needed on part of the medical and dental teachers to carry out effective digital teaching for their students. | The data yielded 47 selective codes with 15 sub-themes and five themes. The emergent themes included general digital competencies, specific digital teaching competencies, mastery of the subject matter, mastery of pedagogical strategies and proficiency in using innovative digital technologies for teaching. The themes 1, 2 and 5 relate to digital competencies only whereas the themes 3 and 4 are generic competencies which apply to both digital and non-digital teaching. These generic competencies form the basis of all kinds of teaching, hence equally important for digital teaching. | Medical teachers should possess diverse digital competencies. The competency frame-work that emerged in the current research encompasses the essential attributes that should be included in any future training program aiming at the digital capacity building of the teachers. This will keep them primed for effective digital teaching. Given its crucial importance, the digital teaching competency should be considered as a cross-cutting competency that applies to almost all of the famous eight roles of medical teacher. |
| Saha 2024[100] | India | Cross‐sectional survey | Medical students | To explore the relationship between digital literacy and academic performance among medical students, identifying key areas of digital literacy that impact learning outcomes. | The findings indicated a significant positive correlation between high levels of digital literacy and superior academic performance. Students with advanced skills in navigating online resources and data analysis demonstrated particularly strong academic outcomes. The odds ratios for good academic performance were substantially higher among students with higher digital literacy levels, with key areas showing notable influence. The study underscores the importance of digital literacy in medical education, suggesting that significant enhancements in learning outcomes can be achieved by integrating targeted digital skills training. | The results advocate for the inclusion of comprehensive digital literacy programs in medical curricula to align educational strategies with the evolving demands of the healthcare sector. |
| Sahan 2024[101] | Germany | Proof-of-concept study | Medical students | To address the gap in medical school electives on digital health literacy by introducing and evaluating an elective scoping study on the systematic development of different health app concepts designed by students to cultivate essential skills for future health care professionals (ie, mobile health [mHealth] competencies). | In total, 60 students completed the elective and developed 25 health app concepts, most commonly targeting stress management and depression. In addition, disease management and prevention apps were designed for various somatic conditions such as diabetes and chronic pain. The results indicated high overall satisfaction across the 6 courses according to the evaluation questionnaire, with lower scores indicating higher satisfaction on a scale ranging from 1 to 6 (mean 1.70, SD 0.68). Students particularly valued the content, flexibility, support, and structure. While improvements in group work, submissions, and information transfer were suggested, the results underscore the usefulness of the web-based elective. | This quality improvement project provides insights into relevant features for the successful user-centered and creative integration of mHealth competencies into medical education. Key factors for the satisfaction of students involved the participatory mindset, focus on competencies, discussions with app providers, and flexibility. Future efforts should define important learning objectives for digital health literacy and provide recommendations for integration rather than debating the need for digital health integration |
| Salehi 2021[102] | Iran | Cross-sectional study | Medical sciences students | To determine E-health literacy and correlates among medical sciences students in Karaj, Iran 2017. | The mean age of the subjects was 25.5 + 5.6 years, of which 65.3% were male. The medical students constituted the most number of participants (28.6%). Mean eHEALS score was 26.11 + 6.6 years. There was a significant difference between female and male regarding E-health literacy (P= 0.04). Internet was the most commonly used sources for health information (67%%), the majority of the students were members of one of the social networks (77.7%) that telegram and Instagram were among the most popular networks. | Improving search skills and assessing of health resources in students, especially in medical and Para-medical sciences, is recommended. Social networks can be used as channels for the transmission of the health messages due to high usage among students. |
| Saxena 2018[103] | India | Cross-sectional study | Dental students | To assess the digital literacy and smartphone usage amongst the 260 Central Indian dental students including their perspicacity about smartphone/internet usage for learning purposes. The students’ attitude for implementation of digital technology in study programs/education system was also evaluated. | Out of 260 students, 250 were internet users, out of which 56% had internet access all time. 94.23% students owned a smartphone. 46.53% (114/245) students had some app related to the dentistry in their smartphone device. The commonest site for surfing related to knowledge seeking was google scholar (72%) followed by Pubmed and others. Nearly 80% dental students believed that social media helps them in their professional course studies. Post graduate students showed statistitically significant difference from undergraduates and interns in terms of knowledge of keywords, dental apps and reading research journals. 89.23% students were keen for implementation of e-learning in their curriculum. | This study reflects willingness of dental students to adopt digital revolution in dental education which in turn may present an opportunity for educators and policy makers to modify educational methods and thereby advance student’s current learning approaches. |
| Scarbecz 2018[104] | USA | Cross-sectional study | Dental students | To retrospectively analyze survey data regarding IT use and knowledge collected from first-year dental students at one U.S. dental school in 2009, 2012, and 2017 | All students participated in the survey each year, for a response rate of 100%. Annual numbers of participants were 80 (2009), 90 (2012), and 97 (2017), for a total 267 respondents. The students reported that they frequently accessed Internet resources multiple times per day and that frequency of use had increased over time, principally from access via smartphones. These students reported feeling comfortable with higher education IT applications such as learning management systems and computerized testing. However, despite their frequent IT use, the students reported low levels of knowledge about IT security, and the three years of survey data showed that IT security knowledge had declined over time. | Dental students may not be digital natives, but this study found that these students were clearly immersed in IT and utilized IT multiple times each day. Device usage trends showed that smart phones were increasingly being used for tasks formerly done on laptops or even tablets. The study also found significant gaps in the students’ IT knowledge, particularly as it applies to IT security that may place students’ personal data, as well as the data of their patients, at significant risk. Given the importance of protecting personal information, it may behoove dental schools to make comprehensive IT training an integral part of their curricula, for students’ protection as well as that of the patients they treat during the course of their clinical training. Future directions for research might involve the development, implementation, and assessment of IT instruction in dental schools and its impact on both students and faculty. |
| Schuitmaker 2025[105] | Netherlands | Systematic review | Physicians | To address considerations regarding physicians’ competencies in light of the patient- physician relationship in AI-assisted clinical settings and elaborate on normative aspects of AI in clinical settings affecting the competence-related dynamics at the bedside when AI is used in clinical settings. | Our findings emphasize the importance of physicians’ critical human skills, alongside the growing demand for technical and digital competencies. Concrete guidance on physicians’ required competencies in AI-assisted clinical settings remains ambiguous and requires further clarification and specification. Dissensus remains over whether physicians are adequately equipped to use and monitor AI in clinical settings in terms of competencies, skills and expertise, issues of ownership regarding normative guidance, and training of physicians’ skills. | Future research should clearly outline (i) how physicians must be(come) competent in working with AI in clinical settings, (ii) who or what should take ownership of embedding these competencies in a normative and regulatory framework, (iii) investigate conditions for achieving a reasonable amount of trust in AI, and (iv) assess the connection between trust and efficiency in patient care. |
| Scott 2023[106] | Australia | Qualitative research | Physicians | To propose a set of requisite digital health competencies and recommend that the acquisition and evaluation of these competencies become embedded in physician training curricula and continuing professional development programmes. | We propose a set of requisite digital health competencies and recommend that the acquisition and evaluation of these competencies become embedded in physician training curricula and continuing professional development programmes. | If physicians are to practise effectively and equitably within increasingly digitised but resource-limited health care systems, competence in using digital health technologies that will increasingly transform delivery of patient-centred care is required, |
| Sharma 2019[107] | Nepal | Cross-sectional study | Nursing students | To measure eHealth-literacy skills among nursing students of Kathmandu Medical College Teaching Hospital, Nepal. | A total of 152 nursing students with mean age of 19.84+1.62 years participated in the study. While 44.7% perceived that they had average Internet skills, 65.1% found the Internet useful in helping them make decisions about their health, Nursing students had a moderate self-perceived level of eHealth literacy (median 3.69, OR 0.87). Related factors included students’ Internet skills, frequency of using the Internet for health related purposes and self-perception of the usefulness and importance of the Internet. | This study represents a baseline reference for eHealth literacy among nursing students. Students have some basic necessary skills, while other skills still need to be improved. There is a need to pay attention to eHealth-literacy needs of nursing students. |
| Shen 2024[108] | China | Before-and-after intervention comparative study | Nursing students | To evaluate the impact of an online Digital Health and Informatics Course in China on the knowledge and comprehension of key digital health and informatics topics, self-assessment of nursing informatics competencies, and satisfaction among undergraduate nursing students. | A total of 24 undergraduate nursing students were enrolled in the course. All students completed all sessions of this course, resulting in an attendance rate of 100%. Additionally, all students completed both pre- and post- assessments. In terms of the knowledge and comprehension of key digital health and informatics topics, scores of the quiz on knowledge assessment improved from the pre-test [mean pretest score: 78.33 (SD 6.005)] to the post- test [mean post-test score: 83.17 (SD 4.86)] upon completion of the course (*P* < 0.001). Also, students acknowledged that the course enhanced their knowledge and comprehension of informatics and digital health, the benefits of (nursing) informatics in clinical practice, and the role of health care professionals in informatics and digital health. In terms of self-assessment of nursing informatics competencies, scores on nursing informatics attitudes demonstrated significant improvement (*P* < 0.001). Furthermore, students reported high satisfaction with various aspects of this course, including the opportunity to explore broad horizons in informatics for future careers, engaging in group discussions, and analyzing case studies on the use of informatics and digital health in clinical practice. | This Online Digital Health and Informatics education effectively improved undergraduate nursing students’ knowledge and comprehension of the key digital health and informatics topics, nursing informatics attitudes in the self-assessment of nursing informatics competency with high levels of satisfaction. In order to ensure that future education in digital health and informatics for nursing students is in line with the technological advancements in clinical settings, it is necessary to foster collaboration between medical school training and clinical practice. This collaboration should involve the use of clinical examples to illustrate advanced digital health applications and the inclusion of practical exercises on the use of digital health technology in clinical settings. |
| Sinan 2023[109] | Turkey | Descriptive and correlational study | Nursing students | To determine the eHealth literacy levels of nursing students and to predict the influencing factors of eHealth literacy. | The mean age of the students was 21.14 ± 1.62 years and 86.2% were female. The mean eHealth literacy score of the students was 29.28 ± 4.73. Fourth year students had higher eHealth literacy scores than those in any other year of study (*P* < 0.001). Students who use the Internet frequently and always, those who search for health-related information on the Internet, those who find accessing health information on the Internet important, and those who find the Internet useful when making health-decisions had significantly high eHealth literacy scores (*P* < 0.05). | The present study revealed that the majority of the nursing students displayed a moderate eHealth literacy level. Academic level, frequency of Internet use, and search for health-related information on the Internet impacted the eHealth literacy of the students. Therefore, eHealth literacy concepts should be integrated into nursing curricula to improve nursing students’ skills in using information technology and to increase their health literacy level. |
| Tanaka 2020[110] | Japan | Cross-sectional study | Nursing students | To describe undergraduate nursing students' perceived eHealth literacy and learning experiences of eHealth literacy in Japan and to clarify the relationship between these factors. | Of the 353 participants in this study, 69.4% did not know “where to find helpful health resources on the Internet,” 80.2% of those lacked the skills “to evaluate health resources,” and 68.9% could not “differentiate the quality of health resources on the Internet”; few of the participants perceived themselves as having any experience in learning the six domains of eHealth literacy. Very few reported learning about health (43.3%) and scientific (21.8%) literacy. | The low perceived eHealth literacy among participants might reflect lack of knowledge and confidence in eHealth literacy as well as their own low level of health-promoting behaviors; this might influence the quality of health education of clients and their families. Nursing educators should address the lack of eHealth literacy among undergraduate nursing students. |
| Tanasombatkul 2021[111] | Thailand | Cross-sectional study | Medical students | To assess the patterns of internet use, eHL level, and learning outcomes with eHL among medical students at Chiang Mai University. | The mean eHEALS score was 33.45. There was a lower degree of agreement on questions regarding internet usage, having skills to evaluate the resources, and confidence in using health information to make health decisions. The eHEALS score had no statistically significant association with most variables and case report scores, but with the longer time of internet use (p-value = 0.014). Although medical students perceived that they have high eHL levels, they report lower confidence in using the information. Including critical thinking skills for electronic health information in the medical curriculum could be useful. | eHealth literacy levels of medical students in Thailand were relatively high, but some domains of electronic health literacy can be improved, particularly in enhancing their skills and confidence to use eHealth information. Introducing concepts of eHealth literacy early in medical school may be necessary along with training workshops to enhance evidence-based searching and appraising techniques could be useful. Even though our study found no association between eHL and learning outcome, we have added to a growing literature about using eHL skills in the medical education field. Further studies on interventions that will help develop eHealth literacy in developing countries are still needed as this should likely lead to better learning and integration of this 21st-century skill in their practice. |
| Tegegne 2023[112] | Ethiopia | Cross-sectional study | Health professionals | To assess health professionals’ digital literacy level and associated factors in Northwest Ethiopia. | Out of 411 participants, 51.8%(95%Cl,46.9-56.6%) of health professional shad adequate digital literacy. Holding a master's degree (Adjusted OR=2.13, 95%CI:1.18-3.85), access to digital technology (AOR=1.89, 95% CI: 1.12-3.17), having training in digital technology (AOR=1.65,95%CI: 1.05-2.59), and having a positive attitude towards digital health technology (AOR=1.64, 95% CI: 1.02-2.68) were found to be significant factors associated with health professionals digital literacy level of health professionals. | Low level of digital literacy among health professionals was observed, with nearly half (48.2%) of them having poor digital literacy levels. Access to digital technology, training on digital technology, and attitude toward digital health technology were significant factors associated with digital literacy. It is suggested to increase computer accessibility, provide a training program on digital health technology, and promote a positive attitude toward this technology to improve the deployment of health information systems. |
| Tesfa 2022[113] | Ethiopia | Cross-sectional study | Health professionals | To assess the eHealth literacy level and its associated factors among health professionals working in Amhara regional state teaching hospitals, Ethiopia. | A total of 383 participants completed and returned the questionnaire with a response rate of 90.5%. Health professionals demonstrated a moderate level of eHealth literacy (mean 29.21). Most of the professionals were aware of the available health resources located on the internet, and know how to search and locate these resources. However, they lack the ability to distinguish high-quality health resources from low-quality resources. Factors that were significantly associated with eHealth literacy were computer access, computer knowledge, perceived ease of use, and perceived usefulness of eHealth information resources. | It is crucial to provide training and support to health care workers on how to find, interpret, and, most importantly, evaluate the quality of health information found on the internet to improve their eHealth literacy level. Further research is needed to explore the role of eHealth literacy in mitigating pandemics in developing countries. |
| Tong 2025[114] | China | Descriptive qualitative study. | Nursing students | To explore the digital learning experiences of undergraduate nursing students in Chinese higher education institutions, with the aim of informing future improvements in digital teaching and learning. | Nursing students’ digital learning experiences were categorized into three themes: positive digital learning experience, negative digital learning experience, and barriers to digital learning perception of students. Conclusions: With the help of advanced digital technology, students can access rich and diversified learning re- sources anytime and anywhere according to their personal learning pace and interests, which greatly enhances their autonomy and interest in learning. Open online courses have effectively compensated for regional differences in teachers’ qualifications, enabling students in remote areas to enjoy high-quality teaching from top educational institutions and giving a solid impetus for improving nursing education in China. However, with the popularity of digital teaching, its design and innovation shortcomings have gradually emerged: students feel lonely and alienated when learning online, and long for more of the interaction and care found in traditional teaching. At the same time, the lack of regulation and assessment standards has created inequities in learning and affected student motivation. | Digital learning brings unprecedented convenience to students, not only as an innovation in learning styles but also as an essential way to personalize the learning experience. With the help of advanced digital technology, students can access a vast array of learning resources anytime, anywhere, according to their own learning pace and interests, and this flexibility greatly enhances their initiative and interest in learning. Open online courses have significantly reduced the differences in the level of teachers in different regions so that even students in remote areas can receive high-quality teaching from top educational institutions, which has undoubtedly given a solid impetus for improving the level of nursing education in China. However, with the popularity of digital teaching methods, some problems have gradually emerged. Many digital teaching methods need more design and innovation, so students often feel lonely and alienated when learning online and long for the kind of face-to-face interaction and teacher attention they get in traditional teaching. Digital learning also faces the challenge of regulation and assessment standards. Due to the lack of a unified regulatory mechanism and evaluation standards, it is not easy to achieve a fair and objective evaluation of students’ learning outcomes, which, to some extent, leads to inequity in learning and, in turn, affects students motivation to learn, Therefore, the future of digital learning requires more efforts in design and innovation, as well as the strengthening of regulatory and evaluation standards, to ensure that students can enjoy the convenience of technology while having a richer and more positive learning experience. |
| Tubaishat 2016[115] | Jordan | Cross-sectional study | Nursing students | To assess the level of eHealth literacy among Jordanian nursing students in two universities, one public and one private, and to determine the factors that have a contribution to eHealth literacy. | Nursing students should have the skills to locate, use and evaluate online health information. Students have some skills in eHealth literacy, while others need to be improved Nursing educators should incorporate the concepts of eHealth literacy within the curriculum. | Integrating the notion of eHealth literacy into the nursing curriculum will prepare nurses to support patients and their families to correctly access, locate, and evaluate health resources in order to formulate decisions that could affect their health status. This in turn could improve patients' safety and care. These eHealth literacy skills are considered some of the most important nursing informatics competencies. Every nursing student should be effectively prepared to use the internet to locate and evaluate digital health information. |
| Vallo Hult 2025[116] | Sweden | Cross-sectional study | Physicians | To examine resident physicians’ perceptions and experiences of using a digital learning environment as part of their specialist medical training. | The results suggest that (i) sociotechnical aspects and understanding of the context in which the learning takes place contribute to enhancing digital learning for resident physicians; (ii) insights into participants’ perceptions of digital learning emphasize that interactive communication and group discussions are significant for their learning, and (iii) administrative aspects related to course design, lecture management, and instructional support are more important in digital learning environments compared to traditional teaching and learning. | Findings from this study confirm and extend prior studies on digital learning in healthcare, contributing to a better understanding of how digital learning environments, especially virtual lectures and seminars, can be developed and integrated into residency programs and health professions education to increase their usefulness. |
| Wilbanks 2020[117] | UK | Literature review | Healthcare professionals | To summarize the current state-of-the-science on the use of clinical simulations to train healthcare professionals to use electronic health records. | The benefits of using simulation-based training that incorporates an organization's contextual factors include improvement of interdisciplinary team communication, clinical performance, clinician-patient-technology communication skills, and recognition of patient safety issues. | Design considerations for electronic health record training using clinical simulations involve establishing course objectives, identifying outcome measures, establishing content requirements of both the clinical simulation and electronic health record, and providing adequate debriefing. |
| Yarmohammadian 2015[118] | Iran | Review | Master’s degree in medical sciences | To review and develop the evaluation criteria of health information technology course at Master of Science level in Tehran, Shahid Beheshti, Isfahan, Shiraz, and Kashan medical universities in 2012 by using CIPP model. | With studies from various sources, commentary of experts, and based on the CIPP evaluation model, 139 indicators were determined and then evaluated, which were associated with this course based on the three factors of context, input, and process in the areas of human resources professional, academic services, students, directors, faculty, curriculum, budget, facilities, teaching–learning activities, and scientific research activities of students and faculty, and the activities of the library staff. | This study showed that in total, the health information technology course at the Master of Science level is relatively good, but trying to improve and correct it in some areas and continuing the evaluation process seems necessary. |
| Yuan 2023[119] | China | Cross-sectional study | Nurses | To investigate the status of eHealth literacy and knowledge, attitudes, and practice regarding palliative care among nurses, and to examine their relationship. | The median scores of eHEALS and KAP regarding paliative care were 32 (interquartile range [IQR] 29 to 38) and 82 (IQR 54 to 106) points, The results of correlation analysis showed that the KAP regarding palliative care was significantly correlated with eHEALS (rho=0.189, *P* < 0.001). in addition, the results of binary logistic regression analysis demonstrated that the eHEALS score was independently associated with the KAP score regarding palliative care when controlling for sociodemoaraphic factors (OR=2.109: *P* < 0.001). | Nurses who worked in first-class tertiary hospitals have good levels of eHealth literacy, while the overall level of KAP regarding palliative care is moderate. Our findings highlight that the eHEALS score is independently associated with the KAP score regarding palliative care. Therefore, nursing managers should adopt multiple measures to comprehensively improve eHealth literacy among nurses, further enrich their knowledge of palliative care, promote a positive transformation of attitudes towards palliative care, and efficiently implement palliative care practice, in order to promote high-quality development of palliative care. |
| Zahmatkeshan 2024[120] | Iran | Qualitative study | Faculty members of medical schools | To explore the challenges to the development and institutionalization of e-learning content as perceived by faculty members of medical schools in southern Iran. | Three themes and eleven subthemes were extracted based on the qualitative data analysis results. Three main themes included individual challenges, organizational management challenges, and course design challenges. | Based on the present results, the most important challenges explained by the participants of this study for the production and development of electronic content included individual challenges, challenges related to the organizational management, and challenges related to course design. Moreover, we should keep in mind that electronic teaching methods and preparation of e-learning content by professors are different from traditional educational methods. Hence, the senior managers of educational system should make the necessary arrangements and plans for methodological support, technological and technical support, and all kinds of organizational support and create continuous professional development opportunities for professors to minimize the negative effects caused by the rapid changes of electronics era and educational environments on professors and consequently on the quality of educational contents and processes. |
| Zainal 2025[121] | Singapore | Qualitative semistructured interview study | Doctors in executive and organizational leadership roles | To use Singapore as a case study and examine the perspectives of doctors in organizational leadership positions to identify and analyze the barriers to DHC implementation in the undergraduate curriculum of Singapore’s medical schools. It also seeks to apply the Normalization Process Theory (NPT) to address these barriers and bridge the gap between health care systems and digital health education (DHE) training. | A total of 33 doctors participated, 26 of whom are currently in organizational leadership roles and 7 of whom have previously held such positions. A total of 6 barriers were identified: bureaucratic inertia, lack of opportunities to pursue nontraditional career pathways, limited protective mechanisms for experiential learning and experimentation, lack of clear policy guidelines for clinical practice, insufficient integration between medical school education and clinical experience, and poor IT integration within the health care industry. | These barriers are also present in other high-income countries experiencing health care digitalization, highlighting the need for a theoretical framework that broadens the generalizability of existing recommendations. Applying the NPT underscores the importance of addressing these barriers to effectively integrate DHC into the curriculum. The active involvement of multiple stakeholders and the incorporation of continuous feedback mechanisms are essential. Our proposed framework provides concrete, evidence-based, and step-by-step recommendations for implementation practice, supporting the introduction of DHC in undergraduate medical education. |
| Zainal 2023a[122] | Singapore | Qualitative study | Clinical educators and deans or vice deans of education | To explore several considerations that are needed to implement a national curriculum that focuses on training medical students in Singapore with relevant digital competencies for the digital age. | While the schools have introduced some relevant courses, they are not standardized nationally. Moreover, the school’s niche areas have not been leveraged upon for training in digital competencies. Participants across all schools acknowledged that more formal training is needed in digital health, data management, and applying the principles of digital technologies. Participants also noted that the healthcare needs of the population, patient safety, and safe procedures in the utilisation of digital healthcare technologies should be prioritized when determining the competencies needed by students. Additionally, participants high- lighted the need for stronger collaboration among medical schools, and for a stronger link between current curriculum and clinical practice. | The findings highlighted the need for better collaboration among medical schools in the sharing of educational resources and expertise. Furthermore, stronger collaborations with professional bodies and the healthcare system should be established to ensure that the goals and outcomes of medical education and the healthcare system are aligned. |
| Zainal 2023b[123] | Singapore | Case study | Junior doctors | To explore the perspectives of medical trainees on the impartation of digital competencies in Singapore’s medical school curricula. It also considers how the medical school experience can be strengthened in order to bridge potential gaps in the integration of these competencies in the local curricula. | Six key reasons were identified: lack of flexibility and dynamism within the curriculum, dated learning style, limited access to electronic health records, gradual uptake of digital technologies in the healthcare sector, lack of an ecosystem that promotes innovation, and lack of guidance from qualified and available mentors. Equipping medical students with skills relevant to the digital age would benefit from a concerted effort from multiple stakeholders: medical schools, medical educators and innovators, as well as the government. | This study bears important implications for countries that seek to bridge the ‘transformation gap’ brought about by the digital age, which is defined as the sharp divergence between innovations that healthcare providers recognize as important but for which they feel insufficiently prepared. |

References

1. Abou Hashish EA, Alnajjar H: **Digital proficiency: assessing knowledge, attitudes, and skills in digital transformation, health literacy, and artificial intelligence among university nursing students**. *BMC Med Educ* 2024, **24**(1):508.

2. Afra A, Ban M, Bachari SS: **Assessment of Informatics Competency Among Nursing Faculty Members and Its Application in Educating Nursing Students at Abadan Nursing Faculty**. *Journal of Medical Education for Future Demands* 2024, **23**(1):1-9.

3. Ahmad MS, Shaik RA, Alzahrani R, Bhat M, Matbouly G, Ahsan M, Mallick AK, Ahmad RK, Alshehri Ali M, Althbaiti M *et al*: **The effect of medical informatics training on students’ knowledge and attitude to use it in future medical work: a comparative study among Saudi medical students**. *Bangladesh Journal of Medical Science* 2025, **24**:170-177.

4. Alexander LM, Bowser J, Caruthers K, Sekhon L, Statler M, Straker H, Rizzolo D, De La Rosa H, Simeon DT: **Fear Factor: Faculty Perceptions of Artificial Intelligence in Physician Associate Education**. *The journal of physician assistant education : the official journal of the Physician Assistant Education Association* 2025.

5. Alfallaj HA, Afrashtehfar KI, Asiri AK, Almasoud FS, Alnaqa GH, Al-Angari NS: **The Status of Digital Dental Technology Implementation in the Saudi Dental Schools' Curriculum: A National Cross-Sectional Survey for Healthcare Digitization**. *Int J Environ Res Public Health* 2022, **20**(1).

6. Alghasani A, Albarwani M, Alsaadi B, Aljabri A, Al-Harthy N, Abukhader M: **Perception and attitude of medical sciences students towards the use of online Internet information in Oman**. *Research Journal of Pharmacy and Technology* 2023, **16**(11):5462-5469.

7. Alhur A: **Curricular Analysis of Digital Health and Health Informatics in Medical Colleges Across Saudi Arabia**. *Cureus* 2024, **16**(8):e66892.

8. Alipour J, Payandeh A: **Assessing the level of digital health literacy among healthcare workers of teaching hospitals in the southeast of Iran**. *Informatics in Medicine Unlocked* 2022, **29**.

9. Alowais M, Nazar H, Tolley C: **Digital literacy education for UK undergraduate pharmacy students: a mixed-methods study**. *The International journal of pharmacy practice* 2024, **32**(5):413-419.

10. Alowais M, Rudd G, Besa V, Nazar H, Shah T, Tolley C: **Digital literacy in undergraduate pharmacy education: a scoping review**. *Journal of the American Medical Informatics Association : JAMIA* 2024, **31**(3):732-745.

11. Amare EM, Zegeye RT, Wondie AG, Andargie BA: **Surveying the Digital Competencies of Health Profession Educators at Ethiopian Higher Education Institutions**. *Ethiopian journal of health sciences* 2024, **34**(4):281-289.

12. Arienti C, Lazzarini SG, Pollet J, Negrini S: **Students 4 Best Evidence as a digital Problem-Based Learning method to improve Evidence-Based Practice competencies in undergraduate physiotherapy students: an observational study**. *BMJ evidence-based medicine* 2021, **26**(5):251-252.

13. Arthanareeswaran A, Ezhilarasan S: **Challenges and acceptance of e-teaching among medical professionals during the COVID-19 pandemic**. *Research & Development in Medical Education* 2023, **12**(1):1-5.

14. Asal MGR, Alsenany SA, Elzohairy NW, El-Sayed AAI: **The impact of digital competence on pedagogical innovation among nurse educators: The moderating role of artificial intelligence readiness**. *Nurse Education in Practice* 2025, **85**:N.PAG-N.PAG.

15. Aydınlar A, Mavi A, Kütükçü E, Kırımlı EE, Alış D, Akın A, Altıntaş L: **Awareness and level of digital literacy among students receiving health-based education**. *BMC Med Educ* 2024, **24**(1):38.

16. Bleijenbergh R, Mestdagh E, Timmermans O, Van Rompaey B, Kuipers YJ: **Digital adaptability competency for healthcare professionals: a modified explorative e-Delphi study**. *Nurse Education in Practice* 2023, **67**:N.PAG-N.PAG.

17. Bloomfield JG, Fisher M, Davies C, Randall S, Gordon CJ: **Registered nurses' attitudes towards e-learning and technology in healthcare: A cross-sectional survey**. *Nurse education in practice* 2023, **69**:103597.

18. Bosch J, Ludwig C, Fluch-Niebuhr J, Stoevesandt D: **Empowerment for the Digital Transformation: Results of a Structured Blended-Learning On-the-Job Training for Practicing Physicians in Germany**. *Int J Environ Res Public Health* 2022, **19**(20).

19. Brown Wilson C, Slade C, Wong WYA, Peacock A: **Health care students experience of using digital technology in patient care: A scoping review of the literature**. *Nurse Educ Today* 2020, **95**:104580.

20. Buchgraber-Schnalzer B, Tilli M, Beinhauer R, Jelinek-Krickl W, Raab R, Lichtenegger K, Hammer M, Reishofer G, Ritschl H: **cMOOC Recommendations to Enhance AI Literacy Among Healthcare Professionals**. *Studies in health technology and informatics* 2025, **324**:135-140.

21. Car J, Ong QC, Erlikh Fox T, Leightley D, Kemp SJ, Švab I, Tsoi KKF, Sam AH, Kent FM, Hertelendy AJ *et al*: **The Digital Health Competencies in Medical Education Framework: An International Consensus Statement Based on a Delphi Study**. *JAMA Netw Open* 2025, **8**(1):e2453131.

22. Chung J, Cho I: **The need for academic electronic health record systems in nurse education**. *Nurse Educ Today* 2017, **54**:83-88.

23. Curran V, Fleet L, Simmons K, Lannon H, Gustafson DL, Wang C, Garmsiri M, Wetsch L: **Adoption and Use of Mobile Learning in Continuing Professional Development by Health and Human Services Professionals**. *The Journal of continuing education in the health professions* 2019, **39**(2):76-85.

24. Davies AC, Davies A, Abdulhussein H, Hooley F, Eleftheriou I, Hassan L, Bromiley PA, Couch P, Wasiuk C, Brass A: **Educating the Healthcare Workforce to Support Digital Transformation**. *Studies in health technology and informatics* 2022, **290**:934-936.

25. Edirippulige S, Gong S, Hathurusinghe M, Jhetam S, Kirk J, Lao H, Leikvold A, Ruelcke J, Yau NC, Zhang Q *et al*: **Medical students' perceptions and expectations regarding digital health education and training: A qualitative study**. *Journal of telemedicine and telecare* 2022, **28**(4):258-265.

26. El Kheir DYM, AlMasmoom NS, Eskander MK, Alshamrani RA, Alwohaibi RN, AlTheeb FN, Aleid BA: **Perception of Saudi undergraduate medical students on telemedicine training and its implementation**. *Journal of family & community medicine* 2023, **30**(3):231-238.

27. Erfani G, McCready J, Gibson B, Nichol B, Unsworth J, Jarva E, Mikkonen K, Tomietto M: **Factors influencing digital health competence among healthcare professionals: A cross-sectional study**. *Applied nursing research : ANR* 2025, **82**:151922.

28. Ersoy H, Baskici C, Aytar A, Strods R, Ratinika NJ, Fernandes AML, Neves H, Blaževičienė A, Vaškelytė A, Wikström-Grotell C *et al*: **Digital competence of faculty members in health sciences measured via self-reflection: current status and contextual aspects**. *PeerJ* 2024, **12**(11).

29. Faihs V, Figalist C, Bossert E, Weimann K, Berberat PO, Wijnen-Meijer M: **Medical Students and Their Perceptions of Digital Medicine: a Question of Gender?** *Medical science educator* 2022, **32**(5):941-946.

30. Farooq Z, Imran A, Imran N: **Preparing for the future of healthcare: Digital health literacy among medical students in Lahore, Pakistan**. *Pakistan Journal of Medical Sciences* 2024, **40**(1).

31. Ghaedi R, Shekofteh M, Valizadeh‐Haghi S, Baghestani A: **Perceived and performed electronic health literacy of medical sciences students**. *Health Information & Libraries Journal* 2024, **41**(4):386-393.

32. Gillissen A, Kochanek T, Zupanic M, Ehlers J: **Medical Students' Perceptions towards Digitization and Artificial Intelligence: A Mixed-Methods Study**. *Healthcare (Basel, Switzerland)* 2022, **10**(4).

33. Hah H, Goldin D: **Exploring Care Providers' Perceptions and Current Use of Telehealth Technology at Work, in Daily Life, and in Education: Qualitative and Quantitative Study**. *JMIR Med Educ* 2019, **5**(1):e13350.

34. Hailegebreal S, Sedi TT, Belete S, Mengistu K, Getachew A, Bedada D, Molla M, Shibiru T, Mengiste SA: **Utilization of information and communication technology (ICT) among undergraduate health science students: a cross-sectional study**. *BMC Med Educ* 2022, **22**(1):215.

35. Hare AJ, Soegaard Ballester JM, Gabriel PE, Adusumalli S, Hanson CW: **Training digital natives to transform healthcare: a 5-tiered approach for integrating clinical informatics into undergraduate medical education**. *Journal of the American Medical Informatics Association : JAMIA* 2022, **30**(1):139-143.

36. Hariyati RTS, Handiyani H, Wildani AA, Afriani T, Nuraini T, Amiruddin MH: **Disparate Digital Literacy Levels of Nursing Manager and Staff, Specifically in Nursing Informatics Competencies and Their Causes: A Cross-Sectional Study**. *Journal of Healthcare Leadership* 2024, **16**:415-425.

37. Hassan MM, Aamir S, Malik N, Naeem N, Avais MA, Shaikh GM: **Use of the Computer and Internet by Teachers in Medical Education: A Study at Medical Colleges of Pakistan**. *Pakistan Journal of Medical and Health Sciences* 2022, **16**(11):617-619.

38. Hautz SC, Hoffmann M, Exadaktylos AK, Hautz WE, Sauter TC: **Digital competencies in medical education in Switzerland: an overview of the current situation**. *GMS journal for medical education* 2020, **37**(6):Doc62.

39. Høium K, Erichsen T, Johannessen LM, Raaheim A, Torbjørnsen A: **What characterizes the use of digital technology in bachelor-level practice placements in health programs?** *Nurse education in practice* 2024, **75**:103883.

40. Huang K, Abdullah AS, Ma Z, Urmi DS, He H, Quintiliani L, Friedman RH, Yang J, Yang L: **Attitudes of Chinese health sciences postgraduate students' to the use of information and communication technology in global health research**. *BMC Med Educ* 2019, **19**(1):367.

41. Hübner U, Shaw T, Thye J, Egbert N, Marin H, Ball M: **Towards an International Framework for Recommendations of Core Competencies in Nursing and Inter-Professional Informatics: The TIGER Competency Synthesis Project**. *Studies in health technology and informatics* 2016, **228**:655-659.

42. Izumi T, Majima Y: **Education Methods for Improving the Ability to Use Nursing Information, with a Focus on Issues Related to the Role of the Head Nurse: A Post-Workshop Evaluation**. *Studies in health technology and informatics* 2016, **225**:993-994.

43. Jacobs RJ, Iqbal H, Rana AM, Rana Z, Kane MN: **Predictors of Osteopathic Medical Students' Readiness to Use Health Information Technology**. *The Journal of the American Osteopathic Association* 2017, **117**(12):773-781.

44. Jarva E, Oikarinen A, Andersson J, Pramila-Savukoski S, Hammarén M, Mikkonen K: **Healthcare professionals' digital health competence profiles and associated factors: A cross-sectional study**. *Journal of advanced nursing* 2024, **80**(8):3236-3252.

45. Jidkov L, Alexander M, Bark P, Williams JG, Kay J, Taylor P, Hemingway H, Banerjee A: **Health informatics competencies in postgraduate medical education and training in the UK: a mixed methods study**. *BMJ open* 2019, **9**(3):e025460.

46. Jimenez G, Spinazze P, Matchar D, Koh Choon Huat G, van der Kleij RMJJ, Chavannes NH, Car J: **Digital health competencies for primary healthcare professionals: A scoping review**. *International journal of medical informatics* 2020, **143**.

47. Jouparinejad S, Foroughameri G, Khajouei R, Farokhzadian J: **Improving the informatics competency of critical care nurses: results of an interventional study in the southeast of Iran**. *BMC medical informatics and decision making* 2020, **20**(1):220.

48. kahouei M, Alaei S, Shariat Panahi SSG, Zadeh JM: **Strategy of health information seeking among physicians, medical residents, and students after introducing digital library and information technology in teaching hospitals of Iran**. *Journal of Evidence-Based Medicine* 2015, **8**(2):91-97.

49. Kaihlanen AM, Virtanen L, Kainiemi E, Sulosaari V, Heponiemi T: **Continuing Education in Digital Skills for Healthcare Professionals - Mapping of the Current Situation in EU Member States**. *International journal of health policy and management* 2024, **13**:8309.

50. Keep M, Janssen A, McGregor D, Brunner M, Baysari MT, Quinn D, Shaw T: **Mapping eHealth Education: Review of eHealth Content in Health and Medical Degrees at a Metropolitan Tertiary Institute in Australia**. *JMIR Med Educ* 2021, **7**(3):e16440.

51. Khamis N, Aljumaiah R, Alhumaid A, Alraheem H, Alkadi D, Koppel C, Abdulghani HM: **Undergraduate medical students' perspectives of skills, uses and preferences of information technology in medical education: A cross-sectional study in a Saudi Medical College**. *Med Teach* 2018, **40**(sup1):S68-s76.

52. Kinny F, Schlottau S, Ali Sherazi B, Obarcanin E, Läer S: **Digital health in pharmacy education: Elective practical course integrating wearable devices and their generated health data**. *Exploratory Research in Clinical and Social Pharmacy* 2024, **15**.

53. Kröplin J, Maier L, Lenz JH, Romeike B: **Impact of a "Digital Health" Curriculum on Students' Perception About Competence and Relevance of Digital Health Topics for Future Professional Challenges: Prospective Pilot Study**. *JMIR formative research* 2025, **9**:e58940.

54. Kuhn S, Müller N, Kirchgässner E, Ulzheimer L, Deutsch KL: **Digital skills for medical students - qualitative evaluation of the curriculum 4.0 "Medicine in the digital age"**. *GMS journal for medical education* 2020, **37**(6):Doc60.

55. Kühnel C, Salomo S, Pagiatakis H, Hübner J, Seifert P, Freesmeyer M, Gühne F: **Medical Students' and Radiology Technician Trainees' eHealth Literacy and Hygiene Awareness—Asynchronous and Synchronous Digital Hand Hygiene Training in a Single-Center Trial**. *Healthcare (2227-9032)* 2023, **11**(10):1475.

56. Lan NH, Thuy LTB: **E-health literacy of medical students at a university in central Vietnam**. *Indian Journal of Public Health Research and Development* 2020, **11**(2):1299-1304.

57. Lawrence K, Levine DL: **The Digital Determinants of Health: A Guide for Competency Development in Digital Care Delivery for Health Professions Trainees**. *JMIR Med Educ* 2024, **10**:e54173.

58. Le LTT, Tran LT, Dang CS, Nguyen PD, Tran NA, Pham TH, Phan HT, Le XH: **Testing reliability and validity of the Vietnamese version of the eHealth literacy scale (eHEALS) among medical students in Vietnam**. *International journal of medical informatics* 2023, **170**:104962.

59. Lee KH, Kim MG, Lee JH, Lee J, Cho I, Choi M, Han HW, Park M: **Empowering Healthcare through Comprehensive Informatics Education: The Status and Future of Biomedical and Health Informatics Education**. *Healthcare informatics research* 2024, **30**(2):113-126.

60. Lee SY, Kim S, Kim S, Shin Y, Yim JJ, Hwang H, Kwon Y, Kim UN, Do YK: **Assessing statistical literacy in medical students and doctors: a single-centre, cross-sectional survey in South Korea**. *BMJ open* 2025, **15**(4):e095173.

61. Lei J, Meng Q, Li Y, Liang M, Zheng K: **The evolution of medical informatics in China: A retrospective study and lessons learned**. *International journal of medical informatics* 2016, **92**:8-14.

62. Lekalakala-Mokgele E, Lowane MP, Mogale NM: **Knowledge, Perceptions and Attitudes of eHealth and Health Technology among Nursing Students from Gauteng Province, South Africa**. *Healthcare (Basel, Switzerland)* 2023, **11**(12).

63. Li P, Tan R, Yang T, Meng L: **Current status and associated factors of digital literacy among academic nurse educators: a cross-sectional study**. *BMC Med Educ* 2025, **25**(1):16.

64. Li W, Yu S, Wang M, Li X, Ma G, Ju X, Ling C: **How do life course events affect the accumulation of digital literacy?: Based on qualitative research of 16 medical university teachers of China**. *Medicine* 2024, **103**(27):e38755.

65. Lilly K, Fitzpatrick J, Madigan E: **Barriers to integrating information technology content in doctor of nursing practice curricula**. *Journal of professional nursing : official journal of the American Association of Colleges of Nursing* 2015, **31**(3):187-199.

66. Liu C, Hong Y, Hu WL, Feng L, Chuang YC, Wang BL: **Evaluation and improvement of nursing undergraduates' informatics competencies using a hybrid multi-criteria decision-making model**. *BMC Med Educ* 2024, **24**(1):1514.

67. Livesay K, Walter R, Petersen S, Abdolkhani R, Zhao L, Butler-Henderson K: **Challenges and Needs in Digital Health Practice and Nursing Education Curricula: Gap Analysis Study**. *JMIR Med Educ* 2024, **10**:e54105.

68. Lokmic-Tomkins Z, Choo D, Foley P, Dix S, Wong P, Brand G: **Pre-registration nursing students' perceptions of their baseline digital literacy and what it means for education: A prospective COHORT survey study**. *Nurse Educ Today* 2022, **111**:105308.

69. Lokmic-Tomkins Z, Raghunathan K, Almond H, Booth RG, McBride SG, Tietze M, Honey M, Procter P, Peddle M, McKenna L: **Perspectives on the implementation of health informatics curricula frameworks**. *Contemporary nurse* 2024, **60**(2):178-191.

70. Lungeanu D, Petrica A, Lupusoru R, Marza AM, Mederle OA, Timar B: **Beyond the Digital Competencies of Medical Students: Concerns over Integrating Data Science Basics into the Medical Curriculum**. *Int J Environ Res Public Health* 2022, **19**(23).

71. Machleid F, Kaczmarczyk R, Johann D, Baleiùnas J, Atienza-Carbonell B, von Maltzahn F, Mosch L: **Perceptions of digital health education among European Medical Students: Mixed methods survey**. *Journal of Medical Internet Research* 2020, **22**(8).

72. Mannevaara P, Saranto K, Kinnunen UM, Hübner U: **Recommended target audience, course content and learning arrangements for teaching health informatics competencies: A scoping review**. *Health informatics journal* 2024, **30**(3):14604582241260643.

73. Marsilio M, Calcaterra V, Infante G, Pisarra M, Zuccotti G: **The digital readiness of future physicians: nurturing the post-pandemic medical education**. *BMC health services research* 2024, **24**(1):885.

74. Martinez-Ulloa L, Flores A, Maldonado-Aguayo Y, Fuentealba-Urra S, Rubio A, Guerrero J, Ravazzano C: **Incorporation of a digital health literacy model into the curricula of medical and rehabilitation science courses in higher education**. *Salud, Ciencia y Tecnología* 2024, **4**(1):1-9.

75. Mather CA, Cheng C, Douglas T, Elsworth G, Osborne R: **eHealth Literacy of Australian Undergraduate Health Profession Students: A Descriptive Study**. *Int J Environ Res Public Health* 2022, **19**(17).

76. Mesko B, Győrffy Z, Kollár J: **Digital Literacy in the Medical Curriculum: A Course With Social Media Tools and Gamification**. *JMIR Med Educ* 2015, **1**(2):e6.

77. Nault D, Abatemarco A, Missenda M, Cherpak-Castagna C, Moonaz S: **Piloting an Educational Approach to Assess eHealth Literacy and Evidence-Based Medicine in Integrative Health: A Feasibility and Validation Study**. *Journal of integrative and complementary medicine* 2025, **31**(3):294-300.

78. Nazeha N, Pavagadhi D, Kyaw BM, Car J, Jimenez G, Car LT: **A Digitally Competent Health Workforce: Scoping Review of Educational Frameworks**. *Journal of Medical Internet Research* 2020, **22**(11).

79. Nguyen LH, Nguyen LTK, Nguyen TT, Trong Dam VA, Vu TMT, Nguyen HAS, Vu GT, Latkin CA, Ho RCM, Ho CSH: **Practices, Perceived Benefits, and Barriers Among Medical Students and Health Care Professionals Regarding the Adoption of eHealth in Clinical Settings: Cross-sectional Survey Study**. *JMIR Med Educ* 2022, **8**(3):e34905.

80. O'Brien N, Li E, Chaibva CN, Bravo RG, Kovacevic L, Ayisi-Boateng NK, Lounsbury O, Nwabufo NFF, Senkyire EK, Serafini A *et al*: **Strengths, Weaknesses, Opportunities, and Threats Analysis of the Use of Digital Health Technologies in Primary Health Care in the Sub-Saharan African Region: Qualitative Study**. *Journal of Medical Internet Research* 2023, **25**(1).

81. Ødegaard NB, Røe Y, Dahl-Michelsen T: **"Learning is about being active, but the digital is not really active": physiotherapy teachers' attitudes toward and experiences with digital education**. *Physiotherapy theory and practice* 2024, **40**(3):494-504.

82. Ogundiya O, Rahman TJ, Valnarov-Boulter I, Young TM: **Looking Back on Digital Medical Education Over the Last 25 Years and Looking to the Future: Narrative Review**. *J Med Internet Res* 2024, **26**:e60312.

83. Oo HM, Htun YM, Win TT, Han ZM, Zaw T, Tun KM: **Information and communication technology literacy, knowledge and readiness for electronic medical record system adoption among health professionals in a tertiary hospital, Myanmar: A cross-sectional study**. *PLoS One* 2021, **16**(7):e0253691.

84. Pajari J, Sormunen M, Salminen L, Vauhkonen A, Aura S, Koskinen M, Mikkonen K, Kääriäinen M, Saaranen T: **The Appearance of Digital Competence in the Work of Health Sciences Educators: A Cross-sectional Study**. *Computers, informatics, nursing : CIN* 2022, **40**(9):624-632.

85. Panhwar M, Rajpar SP, Talpur N, Baig QA, Kumar K, Banglani MA: **Information technology application and challenges faced by medical and dental undergraduate students**. *Rawal Medical Journal* 2021, **46**(2):438-441.

86. Park JYE, Min J: **Exploring canadian pharmacy students’ e-health literacy: A mixed methods study**. *Pharmacy Practice* 2020, **18**(1).

87. Park SH, Do KH, Kim S, Park JH, Lim YS: **What should medical students know about artificial intelligence in medicine?** *J Educ Eval Health Prof* 2019, **16**:18.

88. Pokharel PK, Budhathoki SS, Pokharel HP: **Electronic Health Literacy Skills among Medical and Dental Interns at B P Koirala Institute of Health Sciences**. *Journal of Nepal Health Research Council* 2016, **14**(34):159-164.

89. Poncette AS, Glauert DL, Mosch L, Braune K, Balzer F, Back D: **Undergraduate Medical Competencies in Digital Health and Curricular Module Development: Mixed Methods Study**. *Journal of Medical Internet Research* 2020, **22**(10).

90. Potter A, Munsch C, Watson E, Hopkins E, Kitromili S, O'Neill IC, Larbie J, Niittymaki E, Ramsay C, Burke J *et al*: **Identifying Research Priorities in Digital Education for Health Care: Umbrella Review and Modified Delphi Method Study**. *J Med Internet Res* 2025, **27**:e66157.

91. Quek FF, Meldrum S, Hislop J: **A Systematic Scoping Review of the Current Applications of Digital Technology in Undergraduate Surgical Education**. *Cureus* 2025, **17**(1):e77278.

92. Raghunathan K, McKenna L, Peddle M: **Baseline evaluation of nursing students' informatics competency for digital health practice: A descriptive exploratory study**. *Digital health* 2023, **9**:20552076231179051.

93. Rathnayake S, Senevirathna A: **Self-reported eHealth literacy skills among nursing students in Sri Lanka: A cross-sectional study**. *Nurse Educ Today* 2019, **78**:50-56.

94. Ren SS, Xu WZ, Chen Z, Chen JJ: **Digital competency among pediatric healthcare workers and students: a questionnaire survey**. *World journal of pediatrics : WJP* 2025, **21**(2):192-198.

95. Rigas E, Stoicu-Tivadar L, Crişan-Vida M, Billis A, Nikolaidou M, Anastasiadis S, Siakopoulou S, Bamidis P, Mantas J: **Insights on Training and Certification in Digital Health Technologies**. *Studies in health technology and informatics* 2025, **327**:1039-1043.

96. Robabi H, Arbabisarjou A: **Computer literacy among students of Zahedan University of Medical Sciences**. *Global journal of health science* 2015, **7**(4):136-142.

97. Rohan TZ, Nayak R, Yang K, Nambudiri VE, Kim E: **Analysis of Informatics Topics in Accreditation Council for Graduate Medical Education Program Requirements**. *Applied clinical informatics* 2024, **15**(5):1140-1144.

98. Rowland B, You J, Stern S, Bundy R, Moses A, Witek L, Obermiller C, Rosenthal G, Dharod A: **A Longitudinal Graduate Medical Education Curriculum in Clinical Informatics: Function, Structure, and Evaluation**. *Applied clinical informatics* 2025, **16**(1):84-89.

99. Saaiq M, Khan RA, Yasmeen R: **Digital teaching: Developing a structured digital teaching competency framework for medical teachers**. *Med Teach* 2024, **46**(10):1362-1368.

100. Saha A, Chunder R, Majumdar S, Gupta AD: **Cross‐Sectional Investigation of Digital Literacy and its Impact on Learning Outcomes among Medical Students**. *Research Journal of Medical Sciences* 2024, **18**(1):357-361.

101. Sahan F, Guthardt L, Panitz K, Siegel-Kianer A, Eichhof I, Schmitt BD, Apolinario-Hagen J: **Enhancing Digital Health Awareness and mHealth Competencies in Medical Education: Proof-of-Concept Study and Summative Process Evaluation of a Quality Improvement Project**. *JMIR Med Educ* 2024, **10**:e59454.

102. Salehi L, Keikavoosi-Arani L: **Investigation E-health literacy and correlates factors among Alborz medical sciences students: A cross sectional study**. *International Journal of Adolescent Medicine and Health* 2021, **33**(6):409-414.

103. Saxena P, Gupta SK, Mehrotra D, Kamthan S, Sabir H, Katiyar P, Sai Prasad SV: **Assessment of digital literacy and use of smart phones among Central Indian dental students**. *Journal of oral biology and craniofacial research* 2018, **8**(1):40-43.

104. Scarbecz M, DeSchepper EJ: **Trends in First-Year Dental Students' Information Technology Knowledge and Use: Results from a U.S. Dental School in 2009, 2012, and 2017**. *Journal of dental education* 2018, **82**(12):1287-1295.

105. Schuitmaker L, Drogt J, Benders M, Jongsma K: **Physicians’ required competencies in AI-assisted clinical settings: a systematic review**. *British Medical Bulletin* 2025, **153**(1).

106. Scott IA, Shaw T, Slade C, Wan TT, Coorey C, Johnson SLJ, Sullivan CM: **Digital health competencies for the next generation of physicians**. *Internal Medicine Journal* 2023, **53**(6):1042-1049.

107. Sharma S, Oli N, Thapa B: **Electronic health-literacy skills among nursing students**. *Advances in medical education and practice* 2019, **10**:527-532.

108. Shen H, Chen C, Yan S, Hallensleben C, van der Kleij R, Li M, Dai H, Chavannes N, Zhou Y: **Online digital health and informatics education for undergraduate nursing students in China: impacts and recommendations**. *BMC Med Educ* 2024, **24**(1):803.

109. Sinan O, Ayaz-Alkaya S, Akca A: **Predictors of eHealth literacy levels among nursing students: A descriptive and correlational study**. *Nurse Education in Practice* 2023, **68**:N.PAG-N.PAG.

110. Tanaka J, Kuroda H, Igawa N, Sakurai T, Ohnishi M: **Perceived eHealth Literacy and Learning Experiences Among Japanese Undergraduate Nursing Students: A Cross-sectional Study**. *Computers, informatics, nursing : CIN* 2020, **38**(4):198-203.

111. Tanasombatkul K, Pinyopornpanish K, Angkurawaranon C, Buawangpong N, Rojanasumapong A, Jiraporncharoen W: **Is Electronic Health Literacy Associated with Learning Outcomes among Medical Students in the First Clinical Year?: A Cross-Sectional Study**. *European journal of investigation in health, psychology and education* 2021, **11**(3):923-932.

112. Tegegne MD, Tilahun B, Mamuye A, Kerie H, Nurhussien F, Zemen E, Mebratu A, Sisay G, Getachew R, Gebeyehu H *et al*: **Digital literacy level and associated factors among health professionals in a referral and teaching hospital: An implication for future digital health systems implementation**. *Frontiers in public health* 2023, **11**:1130894.

113. Tesfa GA, Yehualashet DE, Ewune HA, Zemeskel AG, Kalayou MH, Seboka BT: **eHealth Literacy and its Associated Factors Among Health Professionals During the COVID-19 Pandemic in Resource-Limited Settings: Cross-sectional Study**. *JMIR formative research* 2022, **6**(7):e36206.

114. Tong L, Niu Y, Xuan Z, Jin S, Wang Y, Xiao Q: **Nursing students' experiences of digital learning in Chinese higher education institutions: A descriptive qualitative study**. *Nurse Educ Today* 2025, **144**:106454.

115. Tubaishat A, Habiballah L: **eHealth literacy among undergraduate nursing students**. *Nurse Educ Today* 2016, **42**:47-52.

116. Vallo Hult H, Abovarda A, Master Östlund C, Pålsson P: **Digital learning strategies in residency education**. *Ann Med* 2025, **57**(1):2440630.

117. Wilbanks BA, Aroke EN: **Using Clinical Simulations to Train Healthcare Professionals to Use Electronic Health Records: A Literature Review**. *Computers, informatics, nursing : CIN* 2020, **38**(11):551-561.

118. Yarmohammadian MH, Mohebbi N: **Review evaluation indicators of health information technology course of master's degree in medical sciences universities' based on CIPP Model**. *Journal of education and health promotion* 2015, **4**:28.

119. Yuan N, Lv Z-H, Wen Y-Y, Sun C-R, Tao T-Y, Qian D: **The relationship between eHealth literacy and palliative care knowledge, attitudes, and practice among nurses: a cross-sectional study**. *BMC nursing* 2023, **22**(1):1-8.

120. Zahmatkeshan M, Naderi Z, Sadeghi Roonizi N, Bijani M: **Exploring the challenges to development and institutionalization of E-learning content as perceived by faculty members of medical schools in Iran: A qualitative content analysis study**. *Heliyon* 2024, **10**(19):e38270.

121. Zainal H, Xiao Hui X, Thumboo J, Kok Yong F: **Organizational Leaders' Views on Digital Health Competencies in Medical Education: Qualitative Semistructured Interview Study**. *JMIR Med Educ* 2025, **11**:e64768.

122. Zainal H, Xiaohui X, Thumboo J, Kok Yong F: **Digital competencies for Singapore's national medical school curriculum: a qualitative study**. *Medical education online* 2023, **28**(1):2211820.

123. Zainal H, Xiaohui X, Thumboo J, Yong FK: **Exploring the views of Singapore junior doctors on medical curricula for the digital age: A case study**. *PLoS One* 2023, **18**(3):e0281108.
